# Reconstructing Coherent Functional Landscape From Multi‐Modal Multi‐Slice Spatial Transcriptomics by a Variational Spatial Gaussian Process

**DOI:** 10.1002/advs.202520423

**Published:** 2026-01-11

**Authors:** Zedong Wang, Bowen Fu, Chuanchao Zhang, Xiaoping Liu

**Affiliations:** ^1^ Key Laboratory of Systems Health Science of Zhejiang Province School of Life Science Hangzhou Institute for Advanced Study University of Chinese Academy of Sciences Hangzhou China

**Keywords:** multi‐modal, spatial transcriptomics, variational spatiotemporal gaussian process

## Abstract

Spatial transcriptomics (ST) technologies are revolutionizing our ability to investigate the spatial organization of complex tissues. While ST has significantly advanced our understanding of tissue architecture, most analytical approaches remain restricted to 2D sections, limiting insights into the full 3D spatial context. Here, we introduce stVGP, a variational spatial Gaussian process framework designed to align, integrate, and reconstruct spatial coherent domains from multi‐modal, multi‐slice ST datasets. By integrating spatial Gaussian processes with spatially hierarchical transformers, stVGP enables accurate cross‐slice alignment, robust batch effect correction, and the identification of biologically meaningful spatial domains. Critically, a key innovation of stVGP is its support for virtual tissue slices generation, allowing for continuous 3D reconstruction and interpolation of gene expression in unsampled regions. Comprehensive evaluations across diverse datasets demonstrate that stVGP consistently outperforms state‐of‐the‐art methods in alignment accuracy, domain detection, and gene expression prediction. Furthermore, stVGP enables cross‐modal generation of gene expression from histological images in human breast cancer samples, facilitating virtual transcriptomic reconstruction with high fidelity. Collectively, stVGP offers a unified, scalable framework for modeling 3D landscapes in complex tissues and developmental systems, bridging the gap between discrete 2D sections and continuous 3D biological insights.

## Graphical abstract

This study introduces stVGP, a variational spatial Gaussian process framework for multi‐modal, multi‐slice spatial transcriptomics. By integrating histological and genomic data through hybrid alignment and attention‐based fusion, stVGP reconstructs coherent 3D functional landscapes. It enables virtual slice generation, cross‐modal gene prediction, and niche deciphering, providing a robust computational tool for mapping complex biological architectures.

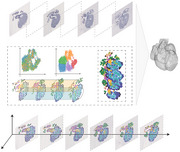

## 1 Introduction

Spatial transcriptomics (ST) technologies enable high‐throughput gene expression profiling while preserving spatial context within tissues in situ [1, 2, 3, 4, 5]. Recent advances have made it possible to collect multiple parallel 2D sections, laying the foundation for reconstructing and analyzing tissue organization in 3D space [6, 7, 8]. Some experimental breakthroughs, such as Open‐ST, have democratized 3D reconstruction by offering cost‐effective, high‐resolution workflows compatible with standard laboratory setups [9]. Simultaneously, large‐scale studies have successfully applied serial‐sectioning strategies to map complex developmental processes, such as the spatiotemporal dynamics of *Drosophila* embryos [10]. Alongside these data generation advances, specialized tools like VT3D have emerged to facilitate the visualization [11]. However, most existing computational approaches are still confined to analyzing individual slices, overlooking the complex spatial relationships across sections [12]. In fact, biological processes inherently span across planes and layers. To bridge this gap, it is essential to develop a comprehensive framework that can seamlessly integrate multiple slices into a coherent 3D model, facilitating an accurate and continuous reconstruction of the biological tissue architecture.

Constructing a 3D atlas from serial ST slices remains a major computational challenge. Precise cross‐slice alignment is complicated by morphological variability, technical inconsistencies in RNA capture, and differences in direction or tissue distortion across slices [13]. However, the efficacy of these sophisticated analyses relies heavily on the quality of the underlying data representation.

In addition, biological tissues are organized into functionally distinct spatial domains [14, 15, 16, 17], which must be consistently identified across slices despite batch effects. Without robust spatial registration and effective domain detection, joint analysis across slices becomes unreliable, hindering downstream interpretation of gene expression dynamics and tissue structure. Therefore, three foundational tasks are essential for 3D spatial modeling: (1) accurate spatial alignment of slices, (2) detection of biologically meaningful spatial domains, and (3) correction of inter‐slice batch effects. Furthermore, the inherent sparsity and partial sampling in spatial patterns in spatial transcriptomics underscore the need for predictive models that can infer expression in unsampled regions to achieve spatial continuity.

Several methods have been proposed to tackle parts of these challenges. For slice alignment, tools such as PASTE [18], PASTE2 [19], STitch3D [20], STAligner [21], and GPSA [22] leverage optimal transport, edge alignment, mutual nearest neighbors, or Gaussian processes in latent space alignment continuous slices. However, these approaches often fail to capture both local and global spatial correspondences simultaneously, and many of the approaches neglect expression‐level batch effects. For domain detection, methods like GraphST [23], SCAN‐IT [24], SpaGT [25], and SpaceFlow [26] employ graph‐based or deep learning strategies to identify tissue structures within individual slices, but most of the methods lack mechanisms for spatial alignment or 3D continuity. BASS [27] and BayesSpace [28] utilize Bayesian modeling frameworks to infer spatial domains by incorporating spatial priors and transcriptomic profiles, significantly improving intra‐slice coherence compared to non‐spatial clustering methods. However, their inferential capabilities are strictly confined to 2D slices, lacking the inherent mechanisms to maintain spatial continuity across consecutive slices. Notably, 3D frameworks like STitch3D attempt to rebuild the whole‐brain structure, but it relies solely on edge geometry and fails to integrate transcriptomic signals, resulting in fragmented reconstructions.

To overcome these limitations, we propose a novel variational spatial Gaussian process framework (stVGP) for integrated alignment and reconstruction of multi‐slice, multi‐modal ST data into coherent 3D spatial tissue models. The stVGP simultaneously resolves key computational bottlenecks in 3D ST analysis. It combines variational inference, spatial Gaussian processes, and spatially hierarchical transformers to (i) perform fine‐grained rigid and non‐rigid slice alignment, (ii) detect biologically coherent spatial domains across slices, and (iii) correct batch effects while preserving biological structure. Importantly, stVGP introduces a variational decoder capable of generating virtual slices by predicting gene expression in unmeasured regions, thereby enabling continuous 3D reconstruction beyond physical sampling resolution.

We extensively validate stVGP across diverse biological systems, including the human dorsolateral prefrontal cortex (DLPFC), the adult mouse brain, the developing human heart, the olfactory bulb, and human breast cancer tissue. In different tissue systems, stVGP outperforms state‐of‐the‐art methods in alignment accuracy, spatial domain fidelity, and gene expression recovery. Furthermore, it supports cross‐modal generation of spatial transcriptomic profiles directly from histological images, facilitating high‐fidelity virtual profiling in clinically relevant samples. Together, stVGP provides a unified, scalable solution for modeling complex tissue architecture in 3D, offering a robust computational foundation for spatial transcriptomic analysis across developmental, neurological, and pathological contexts.

## 2 Results

### 2.1 Overview of stVGP

**FIGURE 1:**
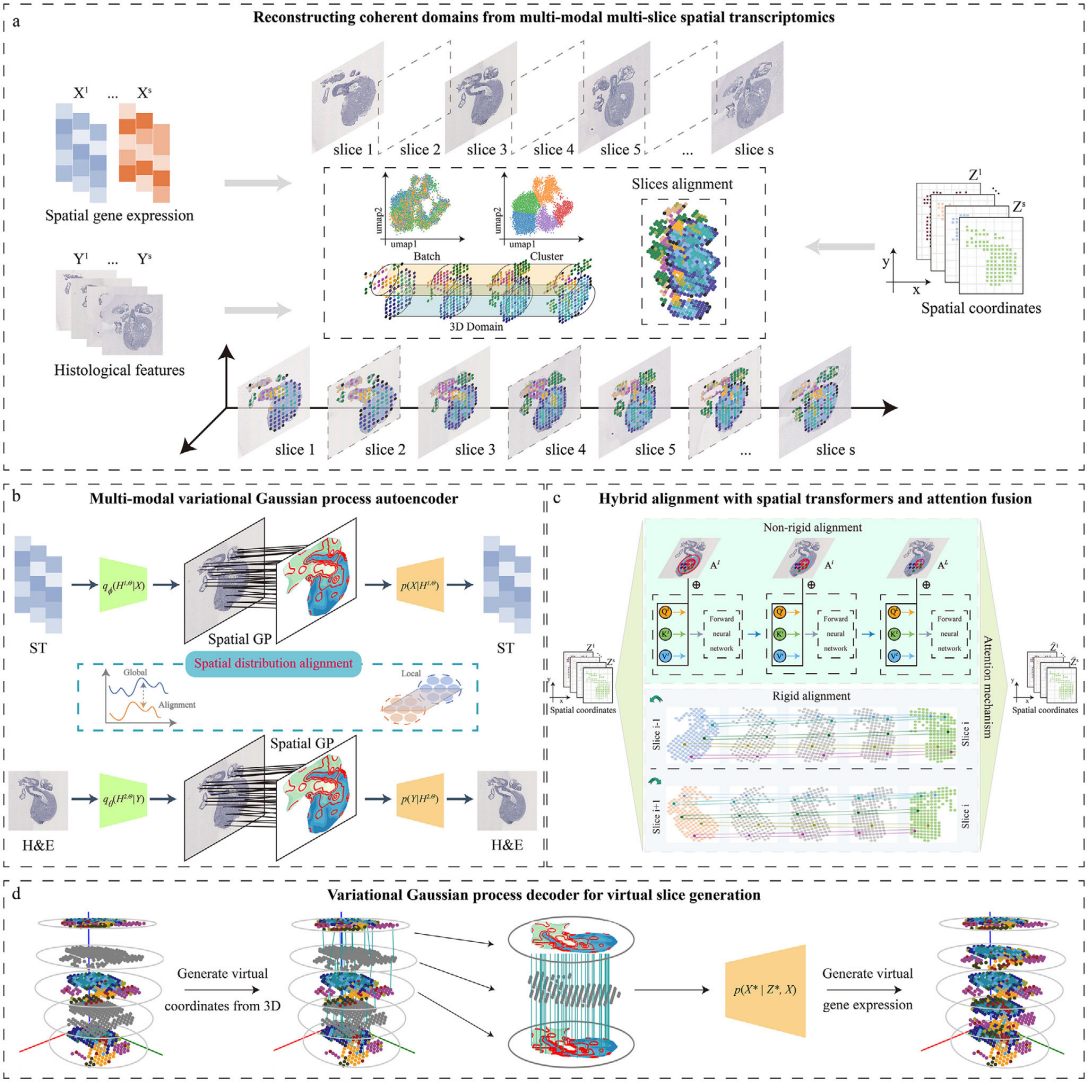
Overview of stVGP. stVGP is a variational spatial Gaussian process framework designed to integrate multi‐modal, multi‐slice spatial transcriptomics (ST) data for coherent 3D tissue reconstruction. It jointly models spatial alignment, domain identification, batch effect correction, and gene expression interpolation across slices. (a) Reconstructing coherent domains from multi‐modal multi‐slice spatial transcriptomics. The inputs to stVGP are a set of 2D ST slices, each consisting of gene expression matrices, histological images and spatial coordinates. During the analysis process, stVGP gradually completes batch correction, domain identification, and 3D alignment. After completing the above analysis, stVGP also performs virtual slice generation and calculates gene expression in unsampled slices. (b) Multi‐modal variational Gaussian process autoencoder. Here, stVGP performs domain identification and batch correction, and aligns spatial gene expression patterns with histological image features. In the latent space, stVGP utilizes Gaussian processes for more precise analysis and enhanced performance. (c) Hybrid alignment with spatial transformers and attention fusion. Here, stVGP performs non‐rigid alignment using a spatial transformer and fuses this with rigid alignment using an attention mechanism. (d) Variational Gaussian process decoder for virtual slice generation. Here, stVGP first registers all slices into a unified 3D coordinate system. Then, stVGP uses the mapping relationship to find the 3D spatial network. Subsequently, stVGP generates a new expression embedding based on the 3D spatial network and Gaussian process. Through decoding, stVGP obtains the final gene expression.

stVGP is a variational spatial Gaussian process framework designed to integrate multi‐modal, multi‐slice spatial transcriptomics (ST) data for coherent 3D tissue reconstruction (Figure 1; Figures S1 and S2). It simultaneously models spatial alignment, domain identification, batch effect correction, and gene expression prediction across slices. The inputs to stVGP are a set of 2D ST slices, each consisting of a gene expression matrix, a histological image and spatial coordinates. The framework comprises three core components: (1) cross‐slice spatial domain detection and batch correction, (2) spatial alignment of tissue slices, and (3) prediction of gene expression in unmeasured regions through virtual slice generation (Figure 1a; Figure S2).

Initially, stVGP produces a spatial representation of a tissue using a variational spatial Gaussian process autoencoder, which models multi‐scale spatial dependencies across slices (Figure 1b; Figure S2). This module enables accurate identification of spatial domains while correcting for slice‐specific batch effects. To enhance biological fidelity, stVGP aligns spatial gene expression patterns with histological image features, ensuring that domain boundaries reflect both transcriptional and morphological coherence. Next, stVGP performs non‐rigid alignment with spatial context constraint by spatial transformers and fuses this with rigid alignment via an attention mechanism (Figure 1c; Figure S2). This hybrid alignment strategy registers all slices into a unified 3D coordinate system, preserving spatial continuity across sections. Finally, to address the discontinuity and sparsity of ST data, stVGP employs a variational spatial Gaussian process decoder to predict gene expression in unsampled spatial locations and generate virtual tissue slices (Figure 1d; Figure S2). This can reconstruct a continuous 3D gene expression and functional landscape and reveal latent transcriptional programs along the spatial axis (Figure 1; Figure S2).

Together, stVGP provides an end‐to‐end solution for integrating multi‐slice, multi‐modal ST data, achieving accurate spatial alignment, biologically coherent domain detection, effective batch correction, and continuous 3D tissue reconstruction.

### 2.2 Accurately Rebuilding Spatial Domains and Predicting Gene Expression Across Human Prefrontal Cortex

**FIGURE 2:**
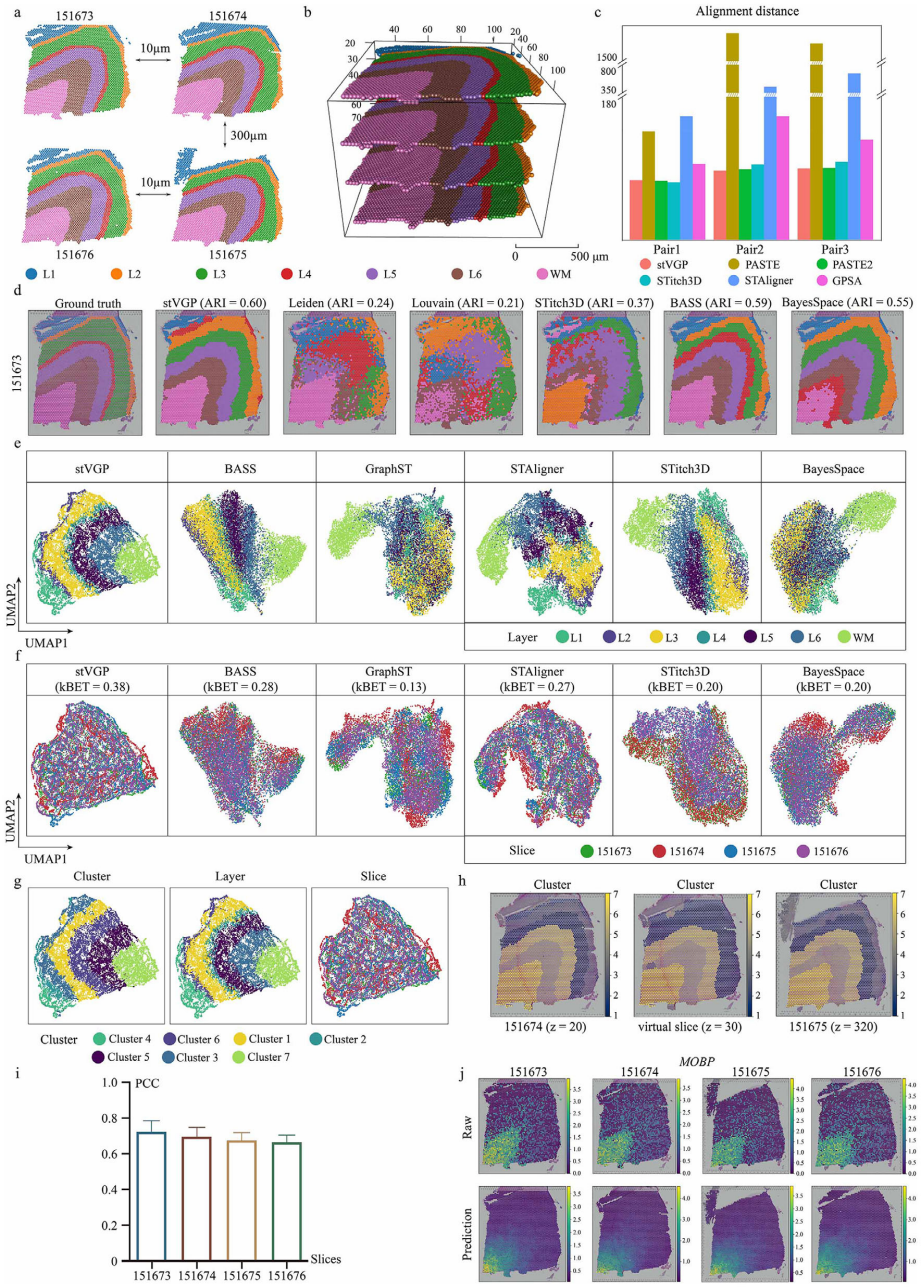
Accurately rebuilding spatial domains and predicting gene expression across human prefrontal cortex. (a) Four adjacent tissue slices from the human dorsolateral prefrontal cortex (DLPFC) dataset (10x Visium). The slices are labeled 151673–151676 (*n* = 3639 spots, 3673 spots, 3592 spots, 3460 spots, respectively), with spacing of 10 µm, 300 µm, and 10 µm, respectively. (b) 3D visualization of manual annotations after aligning the four slices using stVGP. (c) Alignment scores of six computational methods (stVGP, PASTE, PASTE2, STitch3D, STAligner, and GPSA) for three adjacent slice pairs. Higher alignment scores indicate better performance. stVGP achieved the highest scores in the majority of slice pairs. (d) Manual annotation information for slice 151673 and domain identification results from various methods, including stVGP, Leiden, Louvain, STitch3D, STAligner, BASS, and BayesSpace. The ARI values for each method are marked on the methods. (Wilcox signed rank test, *p* < 10^−12^) (e) UMAP visualization of low‐dimensional embeddings, colored by manual annotation labels. (f) UMAP visualization of embeddings colored by slice identity. kBET values (indicating batch mixing performance) are shown for each method. (g) Combined UMAP plots showing embeddings colored by manual annotation, stVGP clustering results, and slice identity, respectively. (h) stVGP domain detection clustering results for two real slices (151674 and 151675) and generated virtual slice. The virtual slice did not exist in the dataset. It was predicted by stVGP from the existing slices to the new spatial locations. The spatial domain of the virtual slice is shown to be consistent with the existing slices. (i) The pearson correlation coefficient of the predicted *MOBP* expression and true expression of *MOBP* in all slices. A higher correlation coefficient indicates a more similar gene expression pattern between prediction and truth. (j) Comparison of *MOBP* gene expression in four slices and stVGP's prediction gene expression.

We evaluated stVGP using a human dorsolateral prefrontal cortex (DLPFC) spatial transcriptomics dataset [7], which contains four adjacent tissue slices (ID: 151673–151676) with varying distances along the DLPFC z‐axis (10 µm, 300 µm, and 10 µm) (Figure 2a). Here, the DLPFC z‐axis denotes the sampling direction along which tissue sections were obtained. These slices exhibit both morphological similarity (151673–151674, 151675–151676) and divergence (151674–151675), and are manually annotated with different cortical layers (labeled as L1‐L6 and white matter (WM) in the original literature) [7].

To evaluate stVGP's spatial alignment performance, we compared stVGP with five representative methods (PASTE [18], PASTE2 [19], STitch3D^20^, STAligner [21], and GPSA [22]). Our results demonstrate that stVGP accurately registered all four slices into a shared 3D space (Figure 2b), including the morphologically distinct pair 151674–151675. Correspondingly, except for PASTE2 and STitch3D, other methods failed to reconstruct the 3D morphology of the tissue (Figure S3). From a quantitative perspective, stVGP consistently outperformed other methods, achieving the highest alignment scores and the lowest alignment distances across most slice pairs. To facilitate evaluation, the four slices were ordered sequentially and divided into three groups of slice pairs (designated as Pair1–3). Specifically, in Pair2 and Pair3, stVGP achieved alignment scores of 0.842 and 0.821, with mean alignment distances of 83.70 and 86.44, respectively. Of the other benchmarked approaches, PASTE2 exhibited the most competitive secondary performance attaining scores of 0.837 and 0.819, with corresponding distances of 85.63 and 86.97 (Figure 2c; Figure S4). Collectively, these results highlight the robustness of stVGP, particularly in handling both small and large inter‐slice variations.

We subsequently evaluated the performance of stVGP in spatial domain detection. On slice 151673, stVGP achieved the highest Adjusted Rand Index (ARI = 0.60) compared to other methods (e.g., BASS (ARI = 0.59) and BayesSpace (ARI = 0.55)), accurately recapitulating known cortical layering (Figure 2d). stVGP also maintained coherent domain segmentation across all slices (Figures S5–S7), as reflected by high ARI and Normalized Mutual Information (NMI) scores (Figures S8–S11). Additionally, we compared with a multi‐modal integration tool, SpaGCN [29], across all slices. Compared to SpaGCN, stVGP achieved more accurate results (ARI = 0.60 in 151673) and a more coherent spatial domain based on manual annotations (Figure S12; Note S1.7).

To evaluate batch effect correction and cross‐slice integration, we visualized the low‐dimensional embeddings of all four slices. stVGP preserved structural boundaries and corrected inter‐slice variance without overcorrection (Figure 2e,f). In contrast, the embeddings generated by other methods exhibited overcorrection, hindering accurate identification of spatial domains (Supplementary Figures S13, S14). Furthermore, other benchmarked methods failed to achieve coherent cross‐slice integration, often resulting in fragmented or misaligned clusters (Figure S15). By contrast, stVGP generated clusters that aligned well with annotated domains, as evidenced by the overlay of original labels on the embedding (Figure 2g). Conversely, competing approaches either failed to effectively merge slices or compromised the structural integrity of the tissue during the integration process.

A central advantage of stVGP is its ability to predict gene expression in unsampled regions via virtual slice generation. We constructed a virtual slice at z  =  30 µm (e.g., DLPFC z‐axis) between slices 151674 and 151675, spanning a 300 µm gap, and found that the predicted slice preserved spatial continuity and accurately interpolated tissue structure (Figure 2h). To quantitatively validate this, we masked each real slice in turn and predicted its gene expression using the remaining slices. Using *MOBP*, a well‐characterized WM marker gene, we found strong correlations between predicted and true expression values across all slices (Figure 2i). Furthermore, the predicted spatial distribution of *MOBP* closely matched biological expectations and showed reduced background noise (Figure 2j). This observation indicates that stVGP concurrently facilitates gene expression denoising and spatial interpolation, yielding a more refined and biologically faithful molecular landscape. In addition, we systematically compared the reconstruction capability of stVGP with other spatial reconstruction methods (STitch3D, GPSA, CellTrek [30], Tangram [31], and spatial linear interpolation). Systematic benchmarking demonstrated that stVGP outperforms existing methods, achieving the highest correlation (>0.68) and lowest RMSE (<0.81) while capturing spot‐level details (Figures S16–S18; Notes S1.8–S1.10). Remarkably, stVGP delivers a superior reconstruction accuracy with exceptional computational efficiency, ranking second only to simple interpolation (Supplementary Figures S16–S18; Notes S1.8–S1.10) [32, 33]. In summary, stVGP not only achieves accurate slice alignment and domain segmentation, but uniquely enables high‐fidelity virtual slice generation, offering a powerful framework for continuous spatial reconstruction from sparsely sampled spatial transcriptomics data.

### 2.3 Decoding the Functional Architecture of the Whole Brain Across the Adult Mouse

We applied stVGP to an extensive and challenging dataset [6] consisting of 35 coronal spatial transcriptomics slices of the adult mouse brain spanning the anterior–posterior (AP; here referred to as AP z‐axis, denoting the sampling direction along which slices were obtained) axis. This complex task necessitates not only the precise alignment of dozens of slices but also the concurrent mitigation of batch effects, the delineation of anatomically coherent 3D spatial domains, and the high‐fidelity reconstruction of a continuous tissue architecture.

**FIGURE 3:**
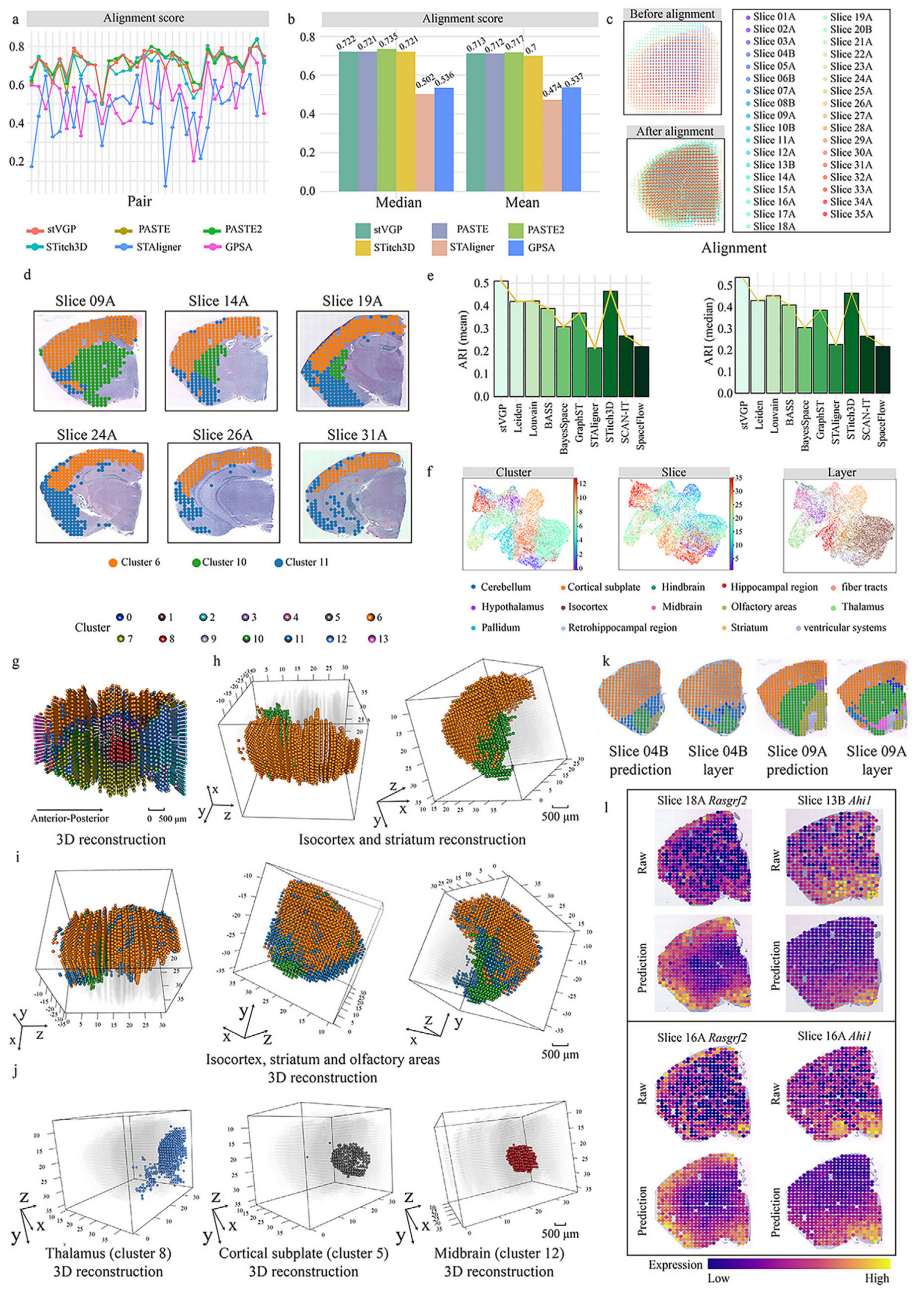
Decoding the functional architecture of the whole brain across adult mouse. (a) Alignment scores of all methods for 34 adjacent slice pairs derived from 35 adult mouse brain slices (*n* = 17018 spots in total). Higher alignment scores indicate better performance. Evaluated methods include stVGP, PASTE, PASTE2, STitch3D, STAligner, and GPSA. (b) Mean and median alignment scores computed across the 34 adjacent slice pairs for each method. (c) 2D visualization of 35 slices before alignment and after stVGP alignment. (d) Visualization of clusters 6, 10, and 11 in stVGP's spatial domain detection results across six representative slices (09A, 14A, 19A, 24A, 26A, 31A). These clusters correspond to the isocortex, striatum, and olfactory areas, respectively. The visualizations highlight dynamic changes in spatial domain distributions across the brain. (e) Median and mean ARI (Adjusted Rand Index) values for clustering results from all domain detection methods, evaluated against manual annotations from the original dataset (Wilcox signed rank test, *p* < 10^−12^). (f) UMAP visualization of embeddings integrated across all slices. Colors indicate: stVGP clustering results, individual slice identities, and manual annotations. stVGP embeddings show strong consistency with manual annotations, while also effectively encoding slice‐level variation in the latent space. (g) 3D reconstruction and visualization of spatial domains identified by stVGP, following alignment of all 35 slices. (h) 3D reconstruction and visualization of isocortex region (cluster 6) and striatum region (cluster 10). (i) 3D reconstruction of the isocortex, striatum, and olfactory areas, presented from three distinct viewing angles, highlighting anatomical structure after spatial alignment. (j) 3D reconstruction of the thalamus (cluster 8), cortical subplate (cluster 5), and midbrain (cluster 12), as repositioned and delineated by stVGP. These regions were accurately localized within the tissue space. (k) Comparison of spatial domains between manual annotations and stVGP‐predicted structures for slices 04B and 09A. (l) Comparison of predicted and actual gene expression levels for *Rasgrf2* (slices 18A, 16A) and *Ahi1* (slices 13B, 16A), showing that stVGP accurately recovered gene‐level spatial patterns.

To evaluate alignment performance, we formed 34 adjacent slice pairs and computed their alignment scores and distances. stVGP exhibited consistently high performance, with the second‐highest mean and median alignment scores among all evaluated methods (Figure 3a,b). Its alignment output produced a more regular, anatomically coherent arrangement from otherwise irregular slices, aligning closely with known brain anatomy (Figure 3c; Figures S19, S20).

The stVGP also successfully identified functionally distinct brain regions across slices, including the isocortex (cluster 6), striatum (cluster 10), and olfactory areas (cluster 11), highlighting its ability to detect 3D spatial domains across the AP z‐axis (Figure 3d). Using the original annotations as ground truth [6], we calculated ARI and NMI, with stVGP achieving the highest ARI and NMI among all methods (Figure 3e), accurately capturing both intra‐slice laminar patterns and inter‐slice tissue transitions. The robustness of these spatial domain delineations was further corroborated by latent space embeddings, where stVGP effectively preserved anatomical organization while removing batch effects (Figure 3f; Figures S21–S23).

Leveraging the aligned slices and consistent domains identified by stVGP, we generated a comprehensive 3D volumetric reconstruction of the adult mouse brain (Figure 3g). Notably, stVGP was able to resolve internal, non‐surface regions that varied gradually along the AP z‐axis, thereby enabling precise localization and structural delineation at the whole‐organ scale (Figure 3g). The resulting model faithfully recapitulated the morphology and spatial continuity of major regions including the isocortex, striatum, and olfactory areas (Figure 3h,i), and internal structures such as the hypothalamus and hippocampus (Figure 3j; Figure S24a–d).

We assessed the generative performance of stVGP through a masking strategy, holding out the fourth and ninth sections to test the model's reconstructive capacity. As a case study, we masked the fourth and ninth slices and evaluated stVGP's reconstruction. The domain structures recovered from these virtual slices closely matched the original annotations, confirming the model's ability to infer tissue organization in unobserved sections (Figure 3k). Furthermore, spatial expression of marker genes—such as those originally highlighted in the dataset—was accurately recovered in reconstructed slices, with enhanced signal clarity and reduced spatial noise (Figure 3l). This underscores stVGP's ability to refine transcriptomic signals while maintaining spatial continuity across the sampling volume.

In summary, stVGP successfully reconstructs the 3D architecture of the adult mouse brain, accurately aligns dozens of slices, identifies biologically consistent spatial domains, corrects batch effects, and predicts gene expression in masked or unsampled regions with high fidelity (Figure 3). These results demonstrate stVGP's capacity to decode complex spatial transcriptomic structures at the whole‐organ scale.

### 2.4 Deciphering the Coherent Domains of the Human Heart Development

To demonstrate the utility of stVGP in developmental biology, we applied it to a spatial transcriptomics dataset of the developing human heart [8], comprising three developmental stages: 4.5–5, 6.5, and 9 post‐conception weeks (PCWs). We concentrated our investigation on nine coronal slices specifically from the 6.5 PCW stage, a period characterized by rapid structural diversification. These slices contain both conserved and heterogeneous features, providing a rigorous testbed for stVGP's capacity to achieve accurate alignment and coherent volumetric modeling in the face of significant morphologicalxbrk variation.

**FIGURE 4:**
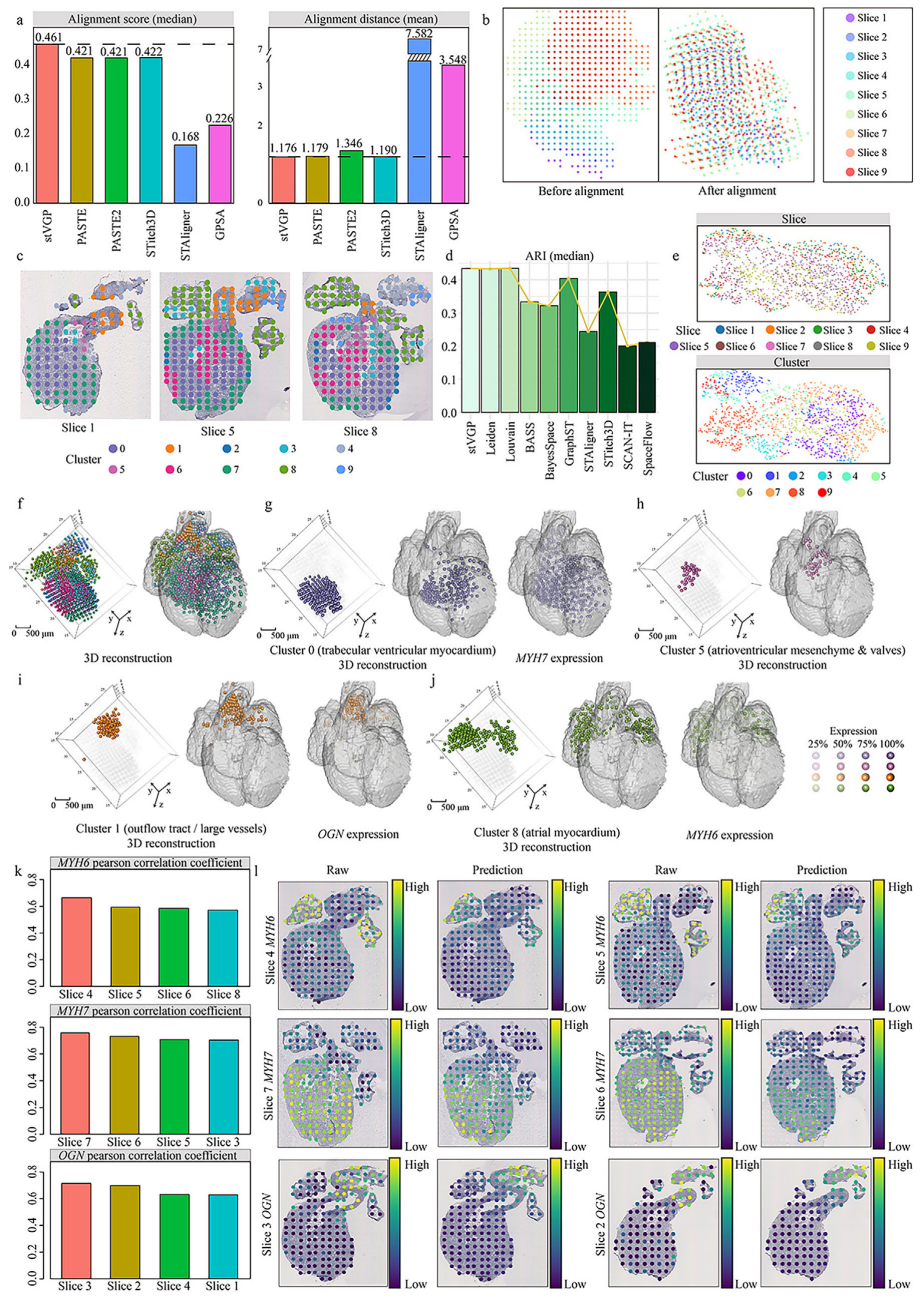
Deciphering the coherent domains of human heart development. (a) Median alignment scores and mean alignment distances of all methods computed across 8 adjacent slice pairs derived from the 9 slices (n = 1480 spots in total). (b) Visualization of all 9 slices before alignment and after alignment using stVGP, showing improved spatial consistency. (c) Spatial domains identified by stVGP in slices 1, 5, and 8. (d) Median Adjusted Rand Index (ARI) scores of various spatial domain detection methods, computed across the nine slices. (e) UMAP plots of low‐dimensional embeddings: colored by slice identity and by stVGP clustering results, demonstrating consistency across developmental stages. (f) Detected spatial domains visualized in the 3D‐aligned coordinates of 6.5‐post‐conception‐week (PCW) slices and mapped into a Carnegie Stage 18 (CS18‐6524) human heart model. (g) Comparison of stVGP localization and reconstruction of cluster 0 (trabecular ventricular myocardium) and its corresponding marker gene *MYH7* in heart model CS18‐6524. (h) Localization and reconstruction of cluster 5 (atrioventricular mesenchyme & valves) by stVGP in heart model CS18‐6524. (i) Comparison of stVGP localization and reconstruction of cluster 1 (outflow tract / large vessels) and its corresponding marker gene *OGN* in heart model of CS18‐6524. (j) Comparison of stVGP localization and reconstruction of cluster 8 (atrial myocardium) and its corresponding tissue marker gene *MYH6* in heart model of CS18‐6524. (k) The pearson correlation coefficient of the predicted *MYH6* expression and true expression of *MYH6* in slices 4, 5, 6, and 8. The pearson correlation coefficient of the predicted *MYH7* expression and true expression of *MYH7* in slices 7, 6, 5, and 3. The pearson correlation coefficient of the predicted *OGN* expression and true expression of *OGN* in slices 3, 2, 4, and 1. A higher correlation coefficient indicates a more similar gene expression pattern between prediction and truth. (l) Comparison between the true expression levels of *MYH7* (slices 4 and 5), *MYH6* (slices 7 and 6), and *OGN* (slices 3 and 2) in tissues and the gene expression levels predicted by stVGP.

We first assessed alignment performance across neighboring slice pairs by computing alignment scores and distances. The stVGP consistently outperformed competing methods, achieving the highest alignment score of 0.461 and the lowest alignment distance of 1.176 (Figure 4a,b). Visual inspection confirmed that stVGP produced the most anatomically coherent results, successfully reconstructing tissue contours from otherwise disorganized slices (Figures S25–S26).

Next, we evaluated stVGP's capability to identify spatially coherent 3D spatial domains. The human heart exhibits key structural features: the left and right atrial myocardium protrusions, the outflow tract and pulmonary vein regions in between, and a ventricular cylindrical body composed of multiple cell types including ventricular cardiomyocytes and epicardial cells [8, 34]. stVGP accurately resolved these spatial domains, capturing both the gross anatomical layout and subtle internal heterogeneity across slices (Figure 4c). Notably, it delineated the atrial and ventricular zones and traced the outflow tract across the anterior–posterior axis, which also corresponded to the slice sampling direction, consistent with known developmental biology [8, 34].

We then compared stVGP's spatial domain detection with other methods (Figure S27). Despite the inherent limitations of unsupervised annotations used as ground truth, stVGP achieved domain segmentation highly concordant with the original study, as shown by ARI comparisons (Figure 4d). Furthermore, low‐dimensional UMAP projections showed that stVGP effectively corrected batch effects and preserved biological variation (Figure 4e; Figure S28). Subsequently, we demonstrated that stVGP can successfully eliminate technical variations (Note S1.11; Figure S29) while retaining essential developmental signals (Note S1.12; Figure S30) from process of batch effects correction.

stVGP also demonstrated its capacity to characterize spatiotemporal features by accurately identifying biologically distinct domains and reconstructing developmental trajectories in the human heart. The analysis linked molecular progression to physical anatomy, revealing a spatial gradient from early trabecular myocardium to more mature states, validated by the specific expression dynamics of key marker genes [8, 34] (Figure S31; Note S1.13).

Having aligned slices and resolved spatial domains, stVGP was next used to perform a 3D reconstruction of cardiac tissue organization. It successfully localized key tissue compartments—including ventricular myocardium, atrial myocardium, outflow tract, and atrioventricular mesenchyme—to anatomically correct positions (Figure 4f–j) [35]. These regions exhibited spatial gene expression patterns consistent with known marker genes (e.g., *MYH6*, *MYH7*, *OGN*) [8], confirming biological fidelity [36, 37] (Figure 4g,i,j). In addition, stVGP captured the layered distribution and gradual morphological changes of the trabecular ventricular myocardium across slices (Figure S32). These findings underscore the framework's superior capacity to resolve subtle, fine‐grained microenvironmental gradients and architectural shifts within complex tissue environments.

Finally, we evaluated stVGP's predictive ability in reconstructing gene expression for unsampled tissue regions. Using a masking strategy, each slice was sequentially masked and we predicted it based on the remaining unmasked slices. To rigorously evaluate the model's spatial fidelity, we selected canonical marker genes representative of distinct cardiac regions: *MYH6* (atrial myocardium), *MYH7* (ventricular myocardium), and *OGN* (outflow tract). These markers serve as established molecular signatures to verify the accuracy of the 3D reconstructed domains. In results, the stVGP achieved high correlation coefficients between predicted and true gene expression across all slices (Figure 4k). Quantitatively, the correlation exceeded 0.60 for most slices, and the predicted spatial expression patterns closely recapitulated the original data (Figure 4k,l). These results highlight stVGP's capability to not only infer missing expression with high accuracy but also facilitate continuous 3D tissue reconstruction across developmental time points.

In summary, stVGP successfully reconstructs the 3D architecture of the developing human heart, accurately aligns spatial slices, identifies functionally distinct tissue domains, and predicts gene expression in unsampled regions (Figure 4). These results prove that stVGP is a robust and versatile framework for interrogating the spatially coordinated transcriptional programs that govern complex human organogenesis.

### 2.5 The Inference of the Trajectory of the Olfactory Bulb in Mice Across Individuals

We further applied stVGP to a mouse olfactory bulb (MOB) spatial transcriptomics dataset [1], comprising three consecutive coronal sections with highly similar morphology and organization. This analysis aimed to evaluate stVGP's ability to align multi‐slice data from highly structured tissue and infer spatial trajectories across layers.

**FIGURE 5:**
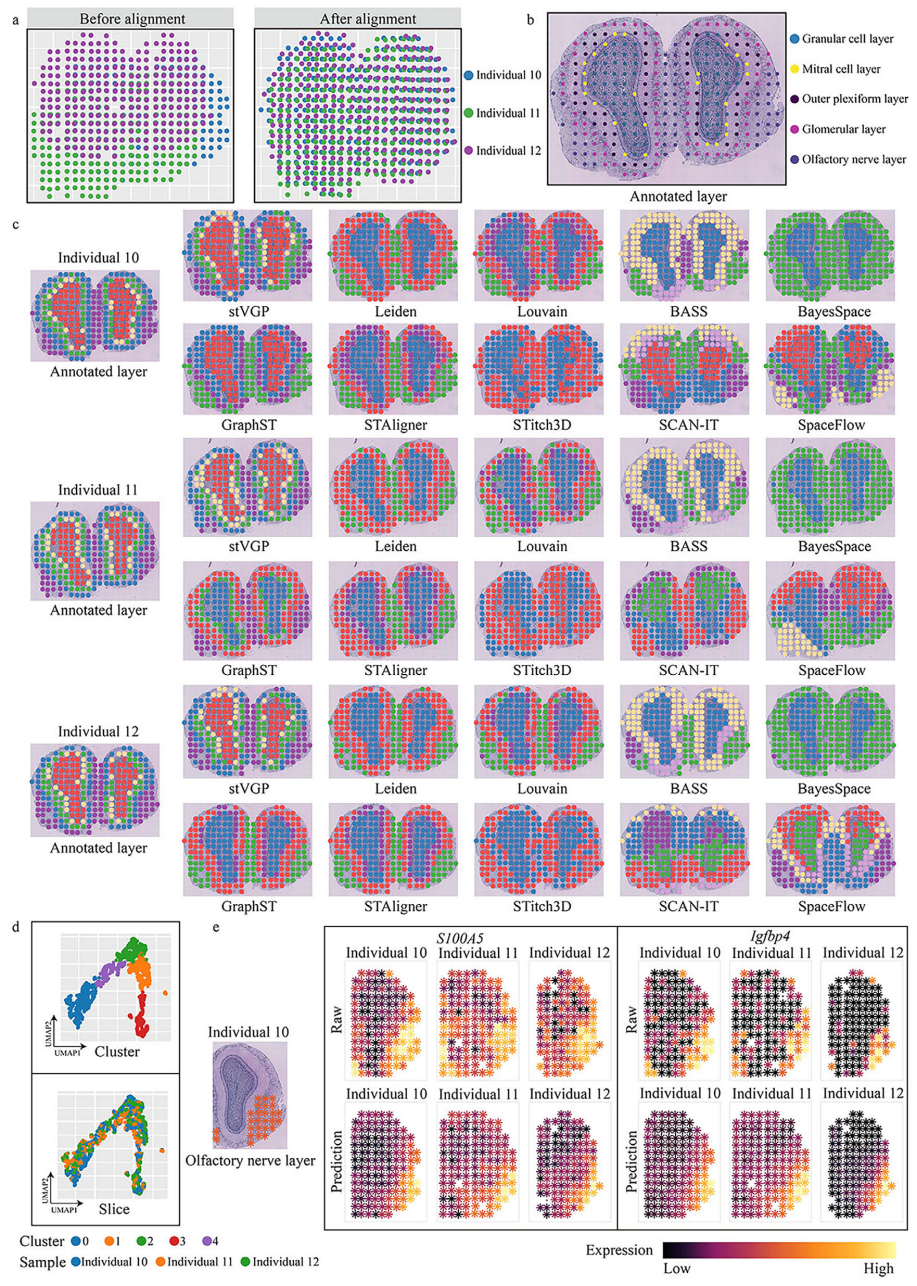
The inference of the trajectory of the olfactory bulb in mice across individuals. (a) Visualization of olfactory bulb slices from three individual mice before and after alignment using stVGP, showing improved spatial correspondence across individuals (*n* = 278 spots, 258 spots, 275 spots, respectively). (b) H&E staining of the olfactory bulb displays five anatomic layers that are organized in an inside‐out fashion: granular cell layer, mitral cell layer, outer plexiform layer, glomerular layer, and olfactory nerve layer. (c) Spatial domains and corresponding manual annotations for the three MOB slices are compared across all methods. Computational methods included stVGP, Leiden, Louvain, BASS, BayesSpace, GraphST, STAligner, STitch3D, SCAN‐IT and SpaceFlow (Wilcox signed rank test, *p* < 10^−12^). (d) UMAP plots of low‐dimensional embeddings, with colors representing stVGP clustering results and three slices information. Here, stVGP demonstrates the ability to infer domain distribution trajectories. (e) Distribution of olfactory nerve layer in individual 10 slice and comparison of the expression of olfactory nerve layer marker genes *S100A5* and *Igfbp4* predicted by stVGP and the true expression levels of them in all tissue slices. In results, stVGP's predicted expression of genes is consistent with the true expression of genes.

We initially evaluated the efficacy of inter‐slice alignment using stVGP. stVGP effectively aligned all three MOB slices (Figure 5a), achieving superior performance compared to other state‐of‐the‐art methods (Figures S33, S34). This high‐fidelity alignment successfully captured the consistent laminar architecture across slices and formed a coherent spatial framework for downstream analysis.

Next, we annotated the MOB tissue layers based on the original study [1]. Using individual 10 slice as an example, five canonical layers were identified from inside to outside: granular cell layer, mitral cell layer, outer plexiform layer, glomerular layer, and olfactory nerve layer (Figure 5b). Similar laminar structures were observed in individual 11 and 12 slices, forming the basis for assessing domain detection.

The stVGP accurately identified all five laminar domains across slices (Figure 5c). The inferred domains showed clear spatial stratification that mirrored known MOB anatomy. Compared with alternative methods, stVGP exhibited superior resolution in detecting fine tissue layers (Figure 5c). In contrast, methods such as Louvain, Leiden, BASS, SCAN‐IT, and SpaceFlow either missed peripheral layers or over‐smoothed the domains into amorphous regions (Figure 5c). While GraphST and STAligner captured most domains, they failed to delineate key boundaries such as the mitral cell layer (Figure 5c).

To evaluate representation quality, we visualized the stVGP's low‐dimensional UMAP embeddings. The embeddings showed clear separation of tissue domains and correct batch effects across slices (Figure 5d), outperforming other methods in preserving spatial identity (Figure S35).

Finally, we tested stVGP's capacity to predict and denoise gene expression in virtual and real slices. For marker genes *S100A5* and *Igfbp4*, stVGP reconstructed expression patterns consistent with the location of the olfactory nerve layer across replicates (Figure 5e). Notably, in the case of *Igfbp4*, stVGP further removed spatial noise, enhancing the clarity of tissue‐specific expression signals.

Together, these results show that stVGP not only aligns MOB slices across individuals and detects consistent spatial domains, but also enables virtual reconstruction and denoising of gene expression, revealing latent tissue structure and spatial trajectories with high fidelity.

### 2.6 Accurate Prediction of Gene Expression Through Cross‐modal Generation in human Breast Cancer

**FIGURE 6:**
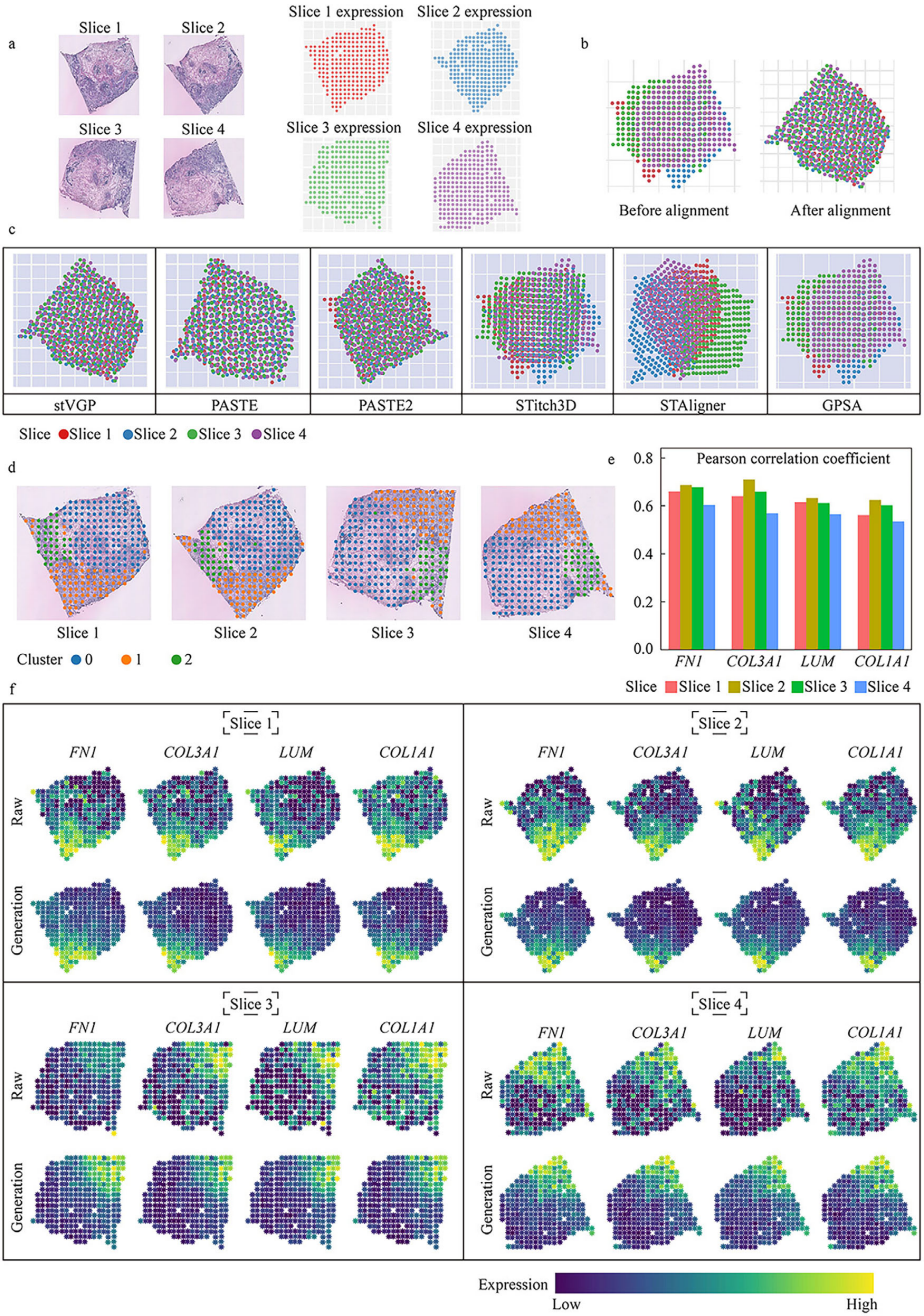
Accurate prediction of gene expression through cross‐modal generation in human breast cancer. (a) The human breast cancer data has four tissue slices (*n* = 254 spots, 251 spots, 264 spots, 262 spots, respectively). These slices are morphologically very similar. But slice 3 and slice 4 are rotated at a large angle to other slices prior to aligned. (b) Visualization of the four breast cancer slices before alignment and visualization of four breast cancer slices after stVGP alignment. (c) Visualization of alignment results for stVGP, PASTE, PASTE2, STitch3D, STAligner, and GPSA methods. (d) Consistent spatial domain conditions in the four slices identified by stVGP. (e) The pearson correlation coefficient of the predicted *FN1*, *COL3A1*, *LUM*, and *COL1A1* expression and true expression of them in slices 1–4. A higher correlation coefficient indicates a more similar gene expression pattern between prediction and truth. (f) Heat map of true expression levels of *FN1*, *COL3A1*, *LUM*, and *COL1A1* and heat map of stVGP‐predicted gene expression. The consistency of the two parts heat maps proved the accuracy of stVGP's performance of the prediction.

To evaluate the cross‐modal predictive capabilities of stVGP, we applied it to a human breast cancer spatial transcriptomics dataset [1], which included four spatial slices with paired H&E‐stained histological images and gene expression matrices. Notably, Slice 3 and Slice 4 exhibited significant rotational differences compared to the other slices (Figure 6a), imposing a significant computational hurdle for spatial registration and subsequent cross‐modal integration.

Despite slice 3 and slice 4 both having large angular discrepancies, stVGP accurately aligned all slices, reconstructing a consistent tissue outline across sections (Figure 6b). Compared to other state‐of‐the‐art alignment methods—including PASTE, PASTE2, STAligner, STitch3D, and GPSA—only PASTE achieved reasonable results, while the others failed to properly handle rotational variance (Figure 6c). Quantitative evaluation further confirmed the superior alignment accuracy of stVGP. Specifically, it achieved the highest alignment score of 0.603 in Pair3 (with the four slices ordered sequentially into three slice‐pair sets) and the second‐lowest median alignment distance of 0.52 across all slices (Figure S36).

Next, we assessed spatial domain detection on all slices. The clustering results from stVGP were morphologically consistent with features in the H&E images (Figure 6d). Specifically, stVGP accurately identified tumor‐enriched regions (Clusters 1 and 2 in Figure 6d), corresponding to densely stained areas in the histology. This performance was found to be equivalent to or superior than that of established spatial domain detection methods (Figure S37). Furthermore, in low‐dimensional embedding space, stVGP demonstrated robust mitigation of batch‐associated technical variations, facilitating a harmonized cross‐sectional interpretation across all tissue sections (Figure S38).

To evaluate cross‐modal gene expression prediction, we selected four marker genes—*FN1*, *COL3A1*, *LUM*, and *COL1A1*—known to show spatially distinct expression patterns associated with the tumor microenvironment. Using only the histological features, stVGP accurately predicted their spatial expression, achieving high correlation coefficients with the ground truth (Figure 6e; Figure S39a–e and Note S1.14). Furthermore, spatial visualization confirmed that stVGP predictions recapitulated the original gene expression landscapes while also denoising background signals, underscoring its superior spatial reconstruction capability (Figure 6f).

In summary, these results demonstrate that stVGP enables accurate cross‐modal generation of spatial transcriptomic profiles from histological images, is robust to tissue distortion and rotational variation, and provides a powerful framework for virtual spatial gene inference in clinical samples.

## 3 Discussion

Understanding the spatial organization of gene expression across complex tissues requires computational methods that can effectively integrate information from multiple spatial transcriptomics (ST) slices while preserving both biological structure and morphological continuity. In this work, we present stVGP, a unified variational spatial Gaussian process framework that simultaneously performs slice alignment, domain detection, batch correction, and virtual slice generation. Unlike existing 3D and multi‐modal integration methods, stVGP can directly model spatial transcriptomics without the requirement of single‐cell reference data, and reduce bias from cross‐platform discrepancies. So, the stVGP can achieve high‐fidelity 3D tissue reconstruction from multi‐modal, multi‐slice ST data.

A key innovation of stVGP lies in its use of spatially constrained variational Gaussian processes to model latent representations across tissue slices. Unlike prior approaches that rely primarily on graph convolutional networks (GCNs) or self‐attention mechanisms to capture spatial dependencies [38], stVGP directly incorporates physical spatial proximity into the Gaussian process kernel. This allows it to exploit the intrinsic geometry of tissue sections, resulting in more biologically coherent spatial domains. GCN‐based methods typically rely on fixed graph structures and may fail to capture complex or non‐local patterns unless explicitly encoded. In contrast, the Gaussian process in stVGP offers continuous, flexible modeling of spatial variation, enabling better resolution of spatial boundaries and robust handling of irregular or sparse spatial sampling [39, 40].

Furthermore, achieving accurate cross‐slice alignment remains a cornerstone for reconstructing seamless 3D spatial landscapes. Within the stVGP framework, we address this challenge through a hybrid alignment strategy that fuses both rigid geometric transformations and non‐rigid deformation modeling. The rigid alignment captures global structural correspondence based on the spatial distribution of highly specific genes, while the spatial transformer network (STN) handles local distortions due to slicing artifacts or tissue warping. The integration of these two views is achieved through an attention‐based fusion mechanism, which adaptively weights the contributions of rigid and non‐rigid alignment at the spot level. This design ensures both global consistency and local flexibility, which is essential for preserving anatomical integrity across slices.

Beyond spatial alignment, another critical aspect of stVGP is its explicit modeling and removal of batch effects between slices. Traditional methods often assume a shared latent space without fully addressing the variation introduced by technical or experimental inconsistencies [41]. stVGP incorporates batch‐specific priors into the variational autoencoder and alignment process, thereby disentangling technical variation from true biological signals. This step significantly improves the robustness of both domain detection and alignment across heterogeneous datasets.

By integrating variational inference, Gaussian process, and deep alignment mechanisms, stVGP represents a comprehensive solution for reconstructing spatially continuous transcriptomic structures from high‐dimensional, multi‐modal ST data. Our evaluations across diverse tissue types demonstrate that stVGP achieves state‐of‐the‐art performance in spatial alignment, domain detection, and gene expression prediction, while also enabling virtual slice generation for continuous 3D modeling. In summary, stVGP offers a novel computational paradigm for spatial transcriptomics, moving beyond discrete slice‐based analysis to a unified, probabilistic modeling framework that captures the complexity of biological tissues in space. Future work may extend this framework to incorporate temporal dynamics, integrate with single‐cell multi‐omics data, or generalize to other spatially resolved modalities beyond transcriptomics.

However, we recognize that the cross‐modal module of stVGP may still possess certain limitations. Specifically, cross‐modal prediction depends on the effective alignment of latent distributions across modalities. Despite mapping data into a shared space, intrinsic differences between modalities can still constrain prediction accuracy. Consequently, relying solely on low‐resolution histological features to predict high‐resolution transcriptomic signals may introduce potential errors. Moreover, our current implementation of distance‐based alignment might overlook feature‐specific discrepancies. To overcome this limitation in future work, we plan to explore more advanced distribution alignment techniques, such as adversarial learning or optimal transport, to further strengthen cross‐modal prediction.

And regarding new ST modalities, Stereo‐seq [42] typically has higher sparsity. Based on our evaluations under sparse conditions (Note S1.20), we think a moderate decrease in stVGP's performance but no drastic degradation. In contrast, MERFISH [43] often profiles a targeted set of genes, which limits feature dimensionality and may impact deep learning‐based frameworks like stVGP (Note S1.20). Consequently, applying stVGP to MERFISH data may require parameter tuning and possibly modality‐specific adjustments. Therefore, moving forward, stVGP will explore algorithmic enhancements to accommodate spatial transcriptomics data across all platforms.

## 4 Methods

We developed stVGP, a variational spatial Gaussian process framework for the integrative analysis of multi‐modal, multi‐slice spatial transcriptomics (ST) data. The goal of stVGP is to align spatial slices, identify coherent spatial domains across slices, correct batch effects, and reconstruct a continuous 3D representation of the tissue with the capacity to predict gene expression in unsampled regions.

### 4.1 Analytical Pipeline of stVGP

The input to stVGP consists of multiple 2D ST slices, each represented by a gene expression matrix $X^{i}\in R^{G\times N_{i}}$ and spatial coordinates $Z^{i}\in R^{2\times N_{i}}$, where *N_i_* is the number of spots in slice *i*, and *G* is the number of genes. If available, histological images can be incorporated as additional spatial priors. We first segment the histological image according to the spatial coordinates of each spot to obtain its partial image. Then, we transform each spot image into 2048‐D latent variables (i.e. $Y^{i}\in R^{2048\times N_{i}}$) by using a pretrained CNN (i.e. ResNet‐50).

The analytical pipeline of stVGP comprises three main modules: (1) Cross‐slice domain detection and batch correction: A multi‐modal variational spatial Gaussian process autoencoder is used to capture spatial patterns across slices and identify coherent spatial domains. Slice‐specific batch effects are modeled and mitigated during this process. (2) Spatial alignment of slices: A dual‐stage alignment is performed, combining non‐rigid registration via spatial transformers with rigid geometric alignment. An attention‐based fusion mechanism integrates both components to register slices into a shared 3D space. (3) Virtual slice generation and expression prediction: A variational Gaussian process decoder is employed to interpolate gene expression across unmeasured spatial regions, allowing prediction in unsampled slices and reconstruction of a smooth 3D gene expression landscape.

### 4.2 Identification of Spatially Variable Genes via Subspace Partitioning

To identify spatially variable genes, stVGP divides each tissue slice into equally‐sized spatial subspace by inserting equally spaced intervals along both horizontal and vertical dimensions. For each subspace, spots are treated as independent samples. Differentially expressed genes are first identified using Seurat, followed by the computation of Moran's I statistic for each gene within each subspace.

Let $m_{j}^{s}$ denote the number of spatial spots in subspace *j* on slice *s*. The Moran's I statistic for gene *i* in subspace *j* is calculated as:

(1) $$I_{i}^{s,j}=\frac{m_{j}^{s}}{\sum_{a}^{m_{j}^{s}}\sum_{b}^{m_{j}^{s}}w_{a,b}}\;\frac{\sum_{a}^{m_{j}^{s}}\sum_{b}^{m_{j}^{s}}w_{a,b}\left(x_{a,i}^{s,j}-{\bar{x}}_{i}^{j}\right)\left(x_{b,i}^{s,j}-{\bar{x}}_{i}^{j}\right)}{\sum_{a}^{m_{j}^{s}}{\left(x_{a,i}^{s,j}-{\bar{x}}_{i}^{j}\right)}^{2}}$$

where $x_{a,i}^{s,j}$ and $x_{b,i}^{s,j}$ represent the expression values of gene *i* at spot *a* and *b* within subspace *j*, respectively, ${\bar{x}}_{i}^{j}$ is the mean expression level of gene *i* in *j* subspace, and *w*_*a*,*b*_ is an element of the spatial weight matrix between spots *a* and *b*.

The stVGP computes the variance of Moran's I values across all subspaces for each gene and selects genes with the highest variance as spatially informative. This procedure ensures that the most spatially variable genes are retained for downstream analysis, facilitating accurate domain detection and alignment.

### 4.3 Multi‐Modal Variational Gaussian Process Autoencoder for Cross‐Slice Domain Detection

To detect coherent tissue domains across slices and correct for inter‐slice variability, stVGP integrates Gaussian processes with variational autoencoders to construct a multi‐modal variational Gaussian process (VGP) autoencoder.

#### 4.3.1 Learning Modal‐Specific Representations from Gene Expression

Let $H^{1,\Theta }={\left[H_{n}^{1}\right]}_{n=1}^{N_{\Theta }}\;\in R^{L\times N_{\Theta }}$ denote the latent representations of gene expression data, where *L* is the dimensionality of the latent space and *N*_Θ_ is the number of spatial spots in batch Θ. In this context, 1 and 2 refer to gene expression and image data, respectively. To account for spatial dependencies and batch effects, we model the latent distribution using a conditional Gaussian process:

(2) $$\begin{array}{ccc} & & p\;\left(H^{1,\Theta }\right)=p\left(h_{1}^{1,\Theta }\right)\prod_{j=2}^{N_{\Theta }}p\left(h_{j}^{1,\Theta }|H_{nei\left(j\right)}^{1,\Theta }\right),\\ & & p\;\left(h_{j}^{1,\Theta }|H_{nei\left(j\right)}^{1,\Theta }\right)=N\left(0,\;k_{\psi }^{j}\left(Z_{nei\left(j\right)},Z_{nei\left(j\right)}\right)\right) \end{array}$$

where $k_{\psi }^{j}$ is a kernel function with parameters ψ. *Z*_*nei*(*j*)_ denotes the coordinates of neighboring spots around spot *j*. Note that, the GP prior is defined solely over spatial coordinates, and that for datasets such as DLPFC, mouse brain, breast cancer, and olfactory bulb, the z‐axis represents physical depth, not time.

The encoder and decoder for gene expression data are defined as:

(3) $$q_{\phi }\;\left(H^{1,\Theta }|X\right)=\prod_{n=1}^{N_{\Theta }}N\left(h_{n}^{1,\Theta }|\mu_{\phi }\left(x_{n}\right),\sigma_{\phi }^{2}\left(x_{n}\right)I_{L}\right)$$

(4) $$p\;\left(H^{1,\Theta }|\Theta \right)=N\left(H^{1,\Theta }|\mu \left(\Theta \right),1\right)$$

(5) $$p_{\theta }\;\left(X|H^{1,\Theta }\right)=\prod_{n=1}^{N_{\Theta }}p_{\theta }\left(x_{n}|h_{n}^{1,\Theta }\right)$$

where the mean μ_ϕ_ and variance $\sigma_{\phi }^{2}$ are given by an encoder network with parameters ϕ. $p_{\theta }\left(x_{n}|h_{n}^{1,\Theta }\right)$ is modelled by a decoder network with parameters θ. *p*(*H* ^1,Θ^|Θ) represents the probability of generating *H* ^1,Θ^ under batch Θ, which is used to eliminate the influence of batch effect during representation learning.

The VGP parameters {ψ,  θ,  ϕ, μ} are jointly optimized by maximizing the evidence lower bound (ELBO):

(6) $$\begin{array}{ccc} L_{exp} & = & \sum_{i=1}^{N_{\Theta }}E_{q\left(h_{i}^{1,\Theta }|x_{i}\right)}\left[logp\left(x_{i}|h_{i}^{1,\Theta }\right)\right]\\ & & -\;\sum_{j=1}^{N_{\Theta }}E_{q\left(H_{nei\left(j\right)}^{1,\Theta }\right)}KL\left[q\left(h_{j}^{1,\Theta }\right)\left|\right|p\left(h_{j}^{1,\Theta }|H_{nei\left(j\right)}^{1,\Theta }\right)\right]\\ & & +\;log\frac{p\left(H^{1,\Theta }|\Theta \right)}{q\left(H^{1,\Theta }|X\right)} \end{array}$$

Details of the derivation are provided in Note S1.2. By setting the number of neighbors to *N*_Θ_, full spatial dependency is retained. Setting the number of neighbors to 0, VGP reduces the model to a standard VAE.

#### 4.3.2 Learning Modal‐Specific Representations from Histological Images

To encode histological features, stVGP uses a similar VGP autoencoder to learn latent representations *H* ^2,Θ^ from image embeddings [44, 45] *Y*:

(7) $$\begin{array}{ccc} L_{img} & = & \sum_{i=1}^{N_{\Theta }}E_{q\left(h_{i}^{2,\Theta }|y_{i}\right)}\left[logp\left(y_{i}|h_{i}^{2,\Theta }\right)\right]\\ & & -\;\sum_{j=1}^{N_{\Theta }}E_{q\left(H_{nei\left(j\right)}^{2,\Theta }\right)}KL\left[q\left(h_{j}^{2,\Theta }\right)\left|\right|p\left(h_{j}^{2,\Theta }|H_{nei\left(j\right)}^{2,\Theta }\right)\right]\\ & & +\;log\frac{p\left(H^{2,\Theta }|\Theta \right)}{q\left(H^{2,\Theta }|Y\right)} \end{array}$$

(8) $$\begin{array}{ccc} & & p\;\left(H^{2,\Theta }\right)=p\left(h_{1}^{2,\Theta }\right)\prod_{j=2}^{N_{\Theta }}p\left(h_{j}^{2,\Theta }|H_{nei\left(j\right)}^{2,\Theta }\right),\\ & & \;p\;\left(h_{j}^{2,\Theta }|H_{nei\left(j\right)}^{2,\Theta }\right)=N\left(0,\;k_{\psi }^{j}\left(Z_{nei\left(j\right)},Z_{nei\left(j\right)}\right)\right) \end{array}$$

#### 4.3.3 Spatial Distribution Alignment Across Modalities

To enforce alignment between gene and image representations, we define alignment losses using Maximum Mean Discrepancy (MMD) and contrastive loss:

(9) $$L_{align\_GD}=MMD\left(H^{1,\Theta },H^{2,\Theta }\right)$$

(10) $$L_{align\_SLD}=\frac{1}{N_{\Theta }}\;\sum_{k=1}^{N}MMD\left({\left(H^{1,\Theta }\right)}_{k}^{Nei},{\left(H^{2,\Theta }\right)}_{k}^{Nei}\right)$$

(11) $$L_{CL}=∥H^{1,\Theta }-H^{2,\Theta }{∥}_{F}^{2}/∥H^{1,\Theta }+H^{2,\Theta }∥$$

The MMD is computed in a reproducing kernel Hilbert space (RKHS):

(12) $$MMD\;\left(X,Y\right)={∥\frac{1}{n}\sum_{i=1}^{n}\Psi \left(x_{i}\right)-\frac{1}{n}\sum_{j=1}^{n}\Psi \left(y_{j}\right)∥}_{H}^{2}\;$$

with kernel $\left(x,y\right)=e^{-\frac{∥x-y{∥}^{2}}{2}}$. ${\left(H^{1,\Theta }\right)}_{k}^{Nei}$ and ${\left(H^{2,\Theta }\right)}_{k}^{Nei}$ denote the representations of spot *k*’s spatial neighbors.

- $L_{align\_GD}$ aligns global distributions for consistent clustering.
- $L_{align\_SLD}$ encourages local spatial consistency.
- $L_{CL}$ regularizes representation drift between modalities.

#### 4.3.4 Joint Optimization

The overall loss function for stVGP is:

(13) $$L_{\mathrm{overall}\_\mathrm{VGP}}=\left(\frac{L_{\exp}+L_{\mathrm{img}}}{\mathrm{VGP}\mathrm{loss}}\right)+\frac{\lambda_{1}\left(L_{\mathrm{CL}}+L_{\mathrm{alig}n_{\mathrm{GD}}}+L_{\mathrm{alig}n_{\mathrm{SLD}}}\right)}{\mathrm{cross}-\mathrm{modal}\mathrm{loss}}$$

where λ_1_ is a tunable weight controlling the strength of alignment constraints (default value is 0.01), and is robust to moderate variation as confirmed by sensitivity analysis (Figure S40a–d; Note S1.15).

### 4.4 Hybrid Alignment With Spatial Transformers and Attention Fusion

To reconstruct coherent 3D tissue architecture from multiple 2D spatial transcriptomics (ST) slices, stVGP integrates both rigid and non‐rigid alignment strategies. This dual approach ensures the preservation of global structural organization while accommodating local morphological variability across slices.

#### 4.4.1 Rigid Alignment using Spatial Landmarks

Let (*X^s^*,*Z^s^*) and (*X* ^s′^,*Z* ^s′^) denote the gene expression and spatial coordinate matrices for the template and alignment slices, respectively, where $Z^{s}\in R^{N_{s}\times 2}$ contains the 2D coordinates of slice *s*. The stVGP selects genes with high spatial specificity, quantified by Moran's I variance, and computes their center of mass coordinates:

(14) $$Z_{g,x}^{s}=\frac{\sum_{n}Z_{n,x}^{s}\times X_{n,g}^{s}}{\sum_{n}Z_{n,x}^{s}}\;,\;Z_{g,y}^{s}=\frac{\sum_{n}Z_{n,y}^{s}\times X_{n,g}^{s}}{\sum_{n}Z_{n,y}^{s}}$$

(15) $$Z_{g,x}^{s^{\prime }}=\frac{\sum_{n}Z_{n,x}^{s^{\prime }}\times X_{n,g}^{s^{\prime }}}{\sum_{n}Z_{n,x}^{s^{\prime }}}\;,\;Z_{g,y}^{s^{\prime }}=\frac{\sum_{n}Z_{n,y}^{s^{\prime }}\times X_{n,g}^{s^{\prime }}}{\sum_{n}Z_{n,y}^{s^{\prime }}}\;$$

Let *m* be the number of selected genes. We stack these coordinates to form landmark matrices:

(16) $$Z_{g}^{s}=\left(Z_{g1}^{s},\text{…},Z_{\mathrm{gm}}^{s}\right),Z_{g1}^{s^{\prime }}=\left(Z_{g1}^{s},\text{…},Z_{\mathrm{gm}}^{s^{\prime }}\right)$$

A multilayer perception (MLP) learns a mapping $f\left(Z_{g}^{s^{\prime }}\right)\to {\hat{Z}}_{rigid}^{s}$, which yields the rigidly aligned coordinates. A third spatial dimension is added post‐alignment to embed each slice into a common 3D coordinate space.

#### 4.4.2 Non‐Rigid Alignment via Spatial Transformer Networks

To model nonlinear deformations from slicing artifacts, stVGP employs a spatial transformer network (STN). Each STN predicts a deformation field per slice that adjusts spatial coordinates at the spot level. The attention mechanism operates as follows for each layer *i*:

(17) $$\begin{array}{ccc} & & Attention\;\left(Q^{i},K^{i},V^{i}\right)=V^{i}E^{i},\;E^{i}\\ & & =\;softmax\left(clip\left(\frac{{\left(Q^{i}\right)}^{T}\cdot K^{i}}{\sqrt{2}}\right)+A^{i}\right) \end{array}$$

where $Q^{i},K^{i},V^{i}\in R^{2\times N}$ are the query, key, and value matrices derived from ϒ^*i*^, the input at layer *i*. Furthermore, a single‐head attention mechanism was employed in this study. *N* represents the number of spots. $A^{i}\in R^{N\times N}$ encodes spatial neighbor similarity, with its locality controlled across layers. As the number of layers *i* increases, the number of adjacent spots selected for *A^i^* decreases. The scaled dot product is constrained by the *clip*(·) operation, improving numerical stability. The final spatially transformed coordinates are:

(18) $$\Upsilon^{i}=\Upsilon^{i-1}+V^{i-1}E^{i-1}$$

(19) $$\Upsilon^{i}=LN\left(\Upsilon^{i}+FFN^{i}\left(LN\left(\Upsilon^{i}\right)\right)\right)$$

where ϒ^0^ = *Z* ^s′^ and $\Upsilon^{L}={\hat{Z}}_{non-rigid}^{s}$ after *L* layers.

#### 4.4.3 Attention‐Based Fusion of Alignment Strategies

To integrate the rigid and non‐rigid alignment results ${\hat{Z}}_{rigid}^{s}$ and ${\hat{Z}}_{non-rigid}^{s}$, we introduce an attention‐based fusion mechanism. For each spot *i*, we compute fusion weights using a shared attention vector $W_{att}\in R^{2\times 1}$:

(20) $$\begin{array}{ccc} & & \left[\alpha_{1}^{i},\alpha_{2}^{i}\;\right]\\ & & =\;softmax\left({W_{att}}^{T}\cdot tanh\left(W\left[{\hat{Z}}_{rigid,i}^{s},{\hat{Z}}_{non-rigid,i}^{s}\right]+\left[b,b\right]\right)\right) \end{array}$$

where $W\in R^{2\times 2}$ and $b\in R^{2\times 1}$ are trainable parameters. Final fused coordinates are computed as:

(21) $$\begin{array}{c} \;{\hat{Z}}^{s}=\alpha_{1}\;{\hat{Z}}_{rigid}^{s}+\alpha_{2}{\hat{Z}}_{non-rigid}^{s} \end{array}$$

#### 4.4.4 Optimization Objective for Alignment

The alignment loss function minimizes the Frobenius norm between the fused coordinates ${\hat{Z}}^{s}$ and the reference coordinates *Z^s^*:

(22) $$L_{align}=min\;{∥{\hat{Z}}^{s}-Z^{s}∥}_{F}^{2}\;$$

This hybrid alignment framework enables accurate spatial registration that preserves both global and local structure, supporting high‐fidelity 3D reconstruction.

### 4.5 Variational Gaussian Process Decoder for Virtual Slice Generation

To achieve continuous 3D reconstruction of spatial transcriptomic landscapes and impute gene expression in unsampled or sparsely measured regions, stVGP integrates a variational Gaussian process (VGP) decoder. This module enables the generation of virtual slices and spatially coherent gene expression interpolation, effectively filling in transcriptomic gaps and denoising spatial measurements across the reconstructed tissue volume.

Formally, we aim to estimate the predictive posterior distribution of gene expression at a novel spatial coordinate Z*, denoted as *p*(X* | Z*,  *X*), conditioned on the observed data *X*. This formulation is widely used in conditional generative tasks. Within the stVGP framework, we approximate this posterior using local neighborhood information based on the latent space learned by the VGP encoder.

Given that VGP encodes spatial correlations through Gaussian processes, we assume that latent variables for a new location H* are primarily influenced by its *K* nearest neighbors in the latent space, indexed by *nei*(*). Based on this, the predictive distribution can be approximated as:

(23) $$q\;\left(H^{*}|X\right)=\int p\left(H^{*}|H_{nei\left(*\right)}\right)q\left(H_{nei\left(*\right)}|X_{nei\left(*\right)}\right)dH_{nei\left(*\right)}$$

(24) $$p\left(X^{*}\;|\;Z^{*},\;X\right)=\int p\left(X^{*}|H^{*}\right)q\left(H^{*}|X\right)dH^{*}$$

In this formulation:

- *p*(H*|*H*_*nei*(*)_) is the Gaussian process prior, which encodes spatial correlation among neighboring latent variables.
- *q*(*H*_*nei*(*)_|*X*_*nei*(*)_) represents the variational posterior inferred from the observed data at nearby locations.
- *p*(X*|H*) is the generative likelihood parameterized by the decoder.

Since *q*(H*|*X*) is Gaussian and fully characterized by its mean and covariance, we can efficiently sample from this distribution using Monte Carlo sampling to approximate the expectation of *p*(X* | Z*,  *X*). This allows stVGP to reconstruct virtual slices, generate smooth gene expression fields across tissue depth, and interpolate unmeasured regions with spatial coherence.

### 4.6 Evaluation of Alignment Accuracy Scores Used in Spatial Transcriptomics Data

To quantitatively evaluate the performance of alignment methods for 3D spatial reconstruction, we employed two complementary metrics: the alignment score and the alignment distance. The alignment score reflects the fidelity of spatial alignment, with higher values indicating greater accuracy and robustness. In contrast, the alignment distance captures the degree of spatial discrepancy between aligned structures, where lower values correspond to more precise and reliable alignments.

#### 4.6.1 Alignment Score

We provide a detailed description of how the alignment score is computed to evaluate the accuracy of spatial alignment between tissue slices. Manual region annotations from the original study were used as the reference standard. Intuitively, if a pair of slices is well‐aligned, the regions with identical annotations in both slices should exhibit substantial spatial overlap. For each aligned pair, we designate slice A as the template slice and slice B as the target slice to be aligned. For every spot in slice B, we calculate its Euclidean distance to all spots in slice A and identify the nearest neighbor. If a spot in slice B and its nearest neighbor in slice A share the same region label, the alignment score is incremented by 1 (starting from 0). This process is repeated for all spots in slice B. Finally, the alignment score is normalized by dividing the total score by the number of spots in slice B, resulting in a final score ranging from 0 to 1. A higher alignment score indicates better alignment, reflecting greater overlap of corresponding annotated regions between adjacent slices. This metric is computed for all tested methods to enable a quantitative comparison of alignment performance.

#### 4.6.2 Alignment Distance

We detail the computation of the alignment distance, which serves as a complementary metric to evaluate spatial alignment accuracy. Manual region annotations provided by the original study were used as a reference. For each aligned slice pair, we define slice A as the template slice and slice B as the target slice to be aligned. In contrast to the alignment score, the alignment distance is computed on a per‐spot basis for all spots in slice B. Specifically, for each spot in slice B, we search for its nearest neighbor in slice A that shares the same region annotation. The Euclidean distance between the spot in slice B and this matched spot in slice A is defined as the alignment distance for that specific spot. As a result, the alignment distance yields a distance vector whose dimension equals the number of spots in slice B. This allows for flexible downstream analyses, such as computing the mean, median, or distribution of alignment distances. The alignment distance captures the spatial variation in region correspondence between slices, with lower values indicating more accurate and consistent alignment.

### 4.7 Statistical Analysis

The statistical *p*‐values reported in Figures 2, 3, and 5c were calculated using the Wilcox signed rank test. To assess statistically significant differences in gene expression, we employed the two‐sided Wilcoxon signed‐rank test. This non‐parametric test was chosen as the validity of the normality assumption could not be ensured for gene expression data. To address the issue of multiple comparisons, *p*‐values were adjusted using the Benjamini‐Hochberg (BH) procedure to control the false discovery rate (FDR). We defined statistical significance as an adjusted *p*‐values (FDR) < 0.05. For pathway enrichment analyses, *p*‐values were computed using Fisher's exact test and adjusted using the BH procedure. All statistical analyses and data processing in this study were performed using Python (version 3.8.19) and R (version 4.2.3). Specifically, the R package Seurat (version 4.3.0) and the Python package Scanpy (version 1.9.8) were used for more detailed data analysis.

## Conflicts of Interest

The authors declare no conflicts of interest.

## Supporting information




**Supporting File**: advs73767‐sup‐0001‐SuppMat.docx.

## Data Availability

The data that support the findings of this study are openly available in DLPFC at [URL/DOI], reference number 29. These data were derived from the following resources available in the public domain: [Resource 1], https://www.[resource1]; [Resource 2], https://www.[resource2];
