# Supplementary material for: Reconstructing Coherent Functional Landscape From Multi‐Modal Multi‐Slice Spatial Transcriptomics by a Variational Spatial Gaussian Process
Source: Adv Sci (Weinh). 2026 Jan 11;13(16):e20423. doi: 10.1002/advs.202520423 (PMC13042375; doi:10.1002/advs.202520423)
Supplement: Supplementary file 1 — Supporting File: advs73767‐sup‐0001‐SuppMat.docx. [file ADVS-13-e20423-s001.docx]

**Reconstructing** **coherent functional landscape from** **multi-modal multi-slice spatial transcriptomics by a variational spatial Gaussian process**

**Zedong Wang^1^, Bowen Fu^1^,Chuanchao Zhang^1,*^, Xiaoping Liu^1,*^**

^1^ Key Laboratory of Systems Health Science of Zhejiang Province, School of Life Science, Hangzhou Institute for Advanced Study, University of Chinese Academy of Sciences, Hangzhou, 310024, China

^*^ To whom correspondence should be addressed. Xiaoping Liu, E-mail: [xpliu@ucas.ac.cn](mailto:xpliu@ucas.ac.cn); Chuanchao Zhang, E-mail: chuanchaozhang@ucas.ac.cn

**Section S1. Supplementary Notes:**

- 1. **Comparison with baseline methods**

To showcase the effectiveness of stVGP in aligning, integrating, and reconstructing spatially coherent domains, we compared stVGP with the state-of-the-art methods, including PASTE^1^, PASTE2^2^, STitch3D^3^, STAligner^4^, GPSA^5^, Leiden^6^, Louvain^7^, BASS^8^, BayesSpace^9^, GraphST^10^, SCAN-IT^11^, and SpaceFlow^12^. All methods were updated to their latest stable versions and the detailed implementation for each method is provided below:

- PASTE (alignment): We followed the workflow specified in the tutorial of the paste GitHub repository (https://github.com/raphael-group/paste). Firstly, we created an AnnData object using raw counts and performed preprocessing on expression data using sc.pp.filter_genes() and sc.pp.filter_cells() from Scanpy package^7^. Finally, we ran paste.pairwise_align() to align all slices. We ran these functions with default parameters.
- PASTE2 (alignment): We followed the workflow specified in the tutorial of the paste2 GitHub repository (https://github.com/raphael-group/paste2/blob/main/tutorial.ipynb). Firstly, we created an AnnData object using raw counts with the help of ad.AnnData() function from anndata package. Then we performed preprocessing on expression data using sc.pp.filter_genes(), sc.pp.filter_cells(), sc.pp.normalize_total(), sc.pp.highly_variable_genes(), and sc.pp.log1p() function Scanpy package. After preprocessing, we ran PASTE2.partial_pairwise_align() and partial_stack_slices_pairwise() functions with default parameters.
- STitch3D (alignment): We followed the workflow specified in the tutorials of STitch3D documentation website (https://stitch3d-tutorial.readthedocs.io/en/latest/tutorials/). Firstly, we created an AnnData object using sc.read_visium() function Scanpy package. Then we ran STitch3D.utils.align_spots() to align all slices with default parameters.
- STAligner (alignment): We followed the workflow specified in the tutorials of STAligner documentation website (https://staligner.readthedocs.io/en/latest/). Firstly, we created an AnnData object using sc.read_visium() function Scanpy package. Then we performed preprocessing on expression data using var_names_make_unique(), sc.pp.filter_genes(), sc.pp.filter_cells(), sc.pp.normalize_total(), sc.pp.highly_variable_genes(), and sc.pp.log1p() function Scanpy package. After preprocessing, we sequentially constructed the spatial network and ran STAligner using STAligner.Cal_Spatial_Net() and STAligner.train_STAligner() respectively. After that, we computed neighbor graph on the latent representation, identified spatial domains and performed UMAP visualization, which were implemented in sc.pp.neighbors(), sc.pp.louvain() and sc.tl.umap() from Scanpy package respectively. Finally, we ran STAligner.ICP_align() align all slices. We ran these functions with default parameters.
- GPSA (alignment): We followed the workflow specified in the tutorials of GPSA GitHub repository (https://github.com/andrewcharlesjones/spatial-alignment). Firstly, we created an AnnData object using sc.read_visium() function Scanpy package. Then we performed preprocessing on expression data using sc.pp.filter_genes(), sc.pp.filter_cells(), sc.pp.normalize_total(), sc.pp.highly_variable_genes(), and sc.pp.log1p() function Scanpy package. After preprocessing, we created VariationalGPSA() and ran model.forward(), train() function from GPSA GitHub repository and gpsa package to align all slices. We ran these functions with default parameters.
- Leiden: We followed the workflow specified in the tutorials of Scanpy documentation website (https://Scanpy-tutorials.readthedocs.io/en/latest/spatial/basic-analysis.html). Firstly, we created an AnnData object using raw counts with the help of ad.AnnData() function from anndata package. Then we performed preprocessing on expression data using sc.pp.filter_genes(), sc.pp.filter_cells(), sc.pp.normalize_total(), sc.pp.highly_variable_genes(), and sc.pp.log1p() function Scanpy package. Finallly, we ran sc.tl.leiden() for spatial domain identification. We ran these functions with default parameters, and the resolution was adjusted to match the number of annotated labels for each dataset.
- Louvain: We followed the workflow specified in the tutorials of Scanpy documentation website (https://Scanpy-tutorials.readthedocs.io/en/latest/spatial/basic-analysis.html). Firstly, we created an AnnData object using raw counts with the help of ad.AnnData() function from anndata package. Then we performed preprocessing on expression data using sc.pp.filter_genes(), sc.pp.filter_cells(), sc.pp.normalize_total(), sc.pp.highly_variable_genes(), and sc.pp.log1p() function Scanpy package. Finallly, we ran sc.tl.louvain() for spatial domain identification. We ran these functions with default parameters, and the resolution was adjusted to match the number of annotated labels for each dataset.
- BASS: We followed the workflow specified in the tutorials of BASS documentation website (https://zhengli09.github.io/BASS-Analysis/). BASS is an R software package. Firstly, we read the raw gene expression data, then we created an object using createBASSObject(), followed by data preprocessing using BASS.preprocess(). Finally, we predicted tissue domains using BASS.run() and BASS.postprocess(). We ran these functions with default parameters.
- BayesSpace: We followed the workflow specified in the tutorials of BayesSpace documentation website (https://www.ezstatconsulting.com/BayesSpace/articles/BayesSpace.html). BayesSpace is an R software package. Firstly, we read the raw gene expression data. Then we we performed preprocessing on expression data using spatialPreprocess() and runUMAP(). After that, we ran spatialCluster() function to identify spatial domains. We ran these functions with default parameters.
- GraphST: We followed the workflow specified in the tutorials of GraphST documentation website (<https://deepst-tutorials.readthedocs.io/en/latest/>). Firstly, we created an AnnData object using sc.read_visium() function Scanpy package. Then we preprocessed the expression data using sc.pp.filter_cells(), sc.pp.filter_genes(), sc.pp.normalize_total(), sc.pp.highly_variable_genes(), and sc.pp.log1p(). After that we created GraphST object using GraphST.GraphST() and trained model. Finally, we computed neighbor graph on the latent representation, identified spatial domains and performed UMAP visualization, which were implemented in sc.pp.neighbors(), sc.pp.leiden() and sc.tl.umap() from Scanpy package respectively. We ran these functions with default parameters.
- SCAN-IT: We followed the workflow specified in the tutorials of SCAN-IT GitHub repository (<https://github.com/zcang/SCAN-IT/blob/main/examples/Slide-seq/scanit.ipynb>). Firstly, we created an AnnData object using sc.read_visium() function Scanpy package. Then we preprocessed the expression data using sc.pp.filter_cells(), sc.pp.filter_genes(), sc.pp.normalize_total(), sc.pp.highly_variable_genes(), and sc.pp.log1p(). After preprocessing data, we constructed the spatial network and ran SCAN-IT using scanit.tl.spatial_graph() and scanit.tl.spatial_representation() respectively. Finally, we computed neighbor graph on the latent representation, identified spatial domains and performed UMAP visualization, which were implemented in sc.pp.neighbors(), sc.pp.leiden() and sc.tl.umap() from Scanpy package respectively. We ran these functions with default parameters. We ran these functions with default parameters.
- SpaceFlow: We followed the workflow specified in the tutorials of SpaceFlow GitHub repository (<https://github.com/hongleir/SpaceFlow/blob/master/tutorials/seqfish_mouse_embryogenesis.ipynb>). Firstly, we created an AnnData object using sc.read_visium() function Scanpy package. Then we preprocessed the expression data using sc.pp.filter_cells(), sc.pp.filter_genes(), sc.pp.normalize_total(), sc.pp.highly_variable_genes(), and sc.pp.log1p(). After that we created SpaceFlow object using SpaceFlow.SpaceFlow and trained model. Finally, we computed neighbor graph on the latent representation, identified spatial domains and performed UMAP visualization, which were implemented in sc.pp.neighbors(), sc.pp.leiden() and sc.tl.umap() from Scanpy package respectively. We ran these functions with default parameters.
  1. **Details of multi-modal variational Gaussian process autoencoder**

To detect coherent tissue domains across slices and correct for inter-slice variability, stVGP integrates Gaussian processes with variational autoencoders to construct a multi-modal variational Gaussian process (VGP) autoencoder^13^.

**Learning modal-specific representations from gene expressions:** Let $H^{1,\Theta}=\left[ H_{n}^{1} \right]_{n=1}^{N_{\Theta}}\in R^{L\times N_{\Theta}}$ denote the latent representations of gene expression data, where $L$ is the dimensionality of the latent space and $N_{\Theta}$ is the number of spatial spots in batch $\Theta$. To account for spatial dependencies and batch effects, we model the latent distribution using a conditional Gaussian process:

$$\begin{aligned} p\left( H^{1,\Theta} \right)=p\left( h_{1}^{1,\Theta} \right)\prod_{j=2}^{N_{\Theta}} p\left( h_{j}^{1,\Theta}|H_{nei\left( j \right)}^{1,\Theta} \right), p\left( h_{j}^{1,\Theta}|H_{nei\left( j \right)}^{1,\Theta} \right)=N\left( 0, k_{\psi}^{j}\left( Z_{nei\left( j \right)},Z_{nei\left( j \right)} \right) \right)\#\left( s1 \right) \end{aligned}$$

where $k_{\psi}^{j}$ is a kernel function with parameters $\psi$. $Z_{nei\left( j \right)}$ denotes the coordinates of neighboring spots around spot *j*. Note that, the GP prior is defined solely over spatial coordinates, and that for datasets such as DLPFC, mouse brain, breast cancer, and olfactory bulb, the z-axis represents physical depth, not time.

The encoder and decoder for gene expression data are defined as:

$$\begin{aligned} q_{\phi}\left( H^{1,\Theta}|X \right)=\prod_{n=1}^{N_{\Theta}} N\left( h_{n}^{1,\Theta}|\mu_{\phi}\left( x_{n} \right),\sigma_{\phi}^{2}\left( x_{n} \right)I_{L} \right)\#\left( s2 \right) \end{aligned}$$

$$\begin{aligned} p\left( H^{1,\Theta}|\Theta\right)=N\left( H^{1,\Theta}|\mu\left( \Theta\right),1 \right)\#\left( s3 \right) \end{aligned}$$

$$\begin{aligned} p_{\theta}\left( X|H^{1,\Theta} \right)=\prod_{n=1}^{N_{\Theta}} p_{\theta}\left( x_{n}|h_{n}^{1,\Theta} \right)\#\left( s4 \right) \end{aligned}$$

where the mean $\mu_{\phi}$ and variance $\sigma_{\phi}^{2}$ are given by an encoder network with parameters $\phi$. $p_{\theta}\left( x_{n}|h_{n}^{1,\Theta} \right)$ is modelled by a decoder network with parameters $\theta$. $p\left( H^{1,\Theta}|\Theta\right)$ represents the probability of generating $H^{1,\Theta}$ under batch $\Theta$, which is used to eliminate the influence of batch effect during representation learning.

We derive the ELBO for VGP and have

$$\log p(\mathbf{X})\geq\int q(H^{1,\Theta})\log\frac{p(\mathbf{X} | H^{1,\Theta})\prod_{j=1}^{N} p(h_{j}^{1,\Theta} | H_{nei\left( j \right)}^{1,\Theta})}{q(H^{1,\Theta})}dH^{1}$$

$$=\int q(H^{1,\Theta})\log p(\mathbf{X} | H^{1,\Theta})dH^{1,\Theta}-\int q\left( H^{1,\Theta} \right)\log\frac{\prod_{j=1}^{N} q\left( h_{j}^{1,\Theta} \right)}{\prod_{j=1}^{N} p(h_{j}^{1,\Theta} | H_{nei\left( j \right)}^{1,\Theta})}dH^{1,\Theta}+\int q\left( H^{1,\Theta}|\mathbf{X} \right)log\frac{p\left( H^{1,\Theta}|\Theta\right)}{q\left( H^{1,\Theta} \right)}dH^{1,\Theta}$$

$$=\int q(H^{1,\Theta})\log p(\mathbf{X} | H^{1,\Theta})dH^{1,\Theta}-\sum_{j=1}^{N} \int q\left( H_{nei\left( j \right)}^{1,\Theta} \right)q\left( h_{j}^{1,\Theta} \right)\log\frac{q(h_{j}^{1,\Theta})}{p(h_{j}^{1,\Theta} | H_{nei\left( j \right)}^{1,\Theta})}dh_{j}^{1,\Theta}dH_{nei\left( j \right)}^{1,\Theta}+log\frac{p\left( H^{1,\Theta}|\Theta\right)}{q\left( H^{1,\Theta}|X \right)}$$

$$=\sum_{\mathcal{i}=1}^{N} \mathbb{E}_{q(h_{j}^{1,\Theta})}\left[ \log p(x_{i}|h_{i}^{1,\Theta}) \right]-\sum_{j=1}^{N} \mathbb{E}_{q(H_{nei\left( j \right)}^{1,\Theta})}\mathrm{KL}\left[ q\left( h_{j}^{1,\Theta} \right)||p\left( h_{j}^{1,\Theta}|H_{nei\left( j \right)}^{1,\Theta} \right) \right]+log\frac{p\left( H^{1,\Theta}|\Theta\right)}{q\left( H^{1,\Theta}|X \right)}$$

The VGP parameters $\{\psi, \theta, \phi\}$ are jointly optimized by maximizing the evidence lower bound (ELBO):

$$\begin{aligned} L_{exp}=\sum_{i=1}^{N_{\Theta}} E_{q\left( h_{i}^{1,\Theta}|x_{i} \right)}\left[ logp\left( x_{i}|h_{i}^{1,\Theta} \right) \right]-\sum_{j=1}^{N_{\Theta}} E_{q\left( H_{nei\left( j \right)}^{1,\Theta} \right)}KL\left[ q\left( h_{j}^{1,\Theta} \right)||p\left( h_{j}^{1,\Theta}|H_{nei\left( j \right)}^{1,\Theta} \right) \right]+log\frac{p\left( H^{1,\Theta}|\Theta\right)}{q\left( H^{1,\Theta}|X \right)}\#\left( s5 \right) \end{aligned}$$

**Learning modal-specific representations from** **histological images:** To encode histological features, stVGP uses a similar VGP autoencoder to learn latent representations $H^{2,\Theta}$ from image embeddings $Y$:

$$\begin{aligned} L_{img}=\sum_{i=1}^{N_{\Theta}} E_{q\left( h_{i}^{2,\Theta}|y_{i} \right)}\left[ logp\left( y_{i}|h_{i}^{2,\Theta} \right) \right]-\sum_{j=1}^{N_{\Theta}} E_{q\left( H_{nei\left( j \right)}^{2,\Theta} \right)}KL\left[ q\left( h_{j}^{2,\Theta} \right)||p\left( h_{j}^{2,\Theta}|H_{nei\left( j \right)}^{2,\Theta} \right) \right]+log\frac{p\left( H^{2,\Theta}|\Theta\right)}{q\left( H^{2,\Theta}|Y \right)}\#\left( s6 \right) \end{aligned}$$

$$\begin{aligned} p\left( H^{2,\Theta} \right)=p\left( h_{1}^{2,\Theta} \right)\prod_{j=2}^{N_{\Theta}} p\left( h_{j}^{2,\Theta}|H_{nei\left( j \right)}^{2,\Theta} \right), p\left( h_{j}^{2,\Theta}|H_{nei\left( j \right)}^{2,\Theta} \right)=N\left( 0, k_{\psi}^{j}\left( Z_{nei\left( j \right)},Z_{nei\left( j \right)} \right) \right)\#\left( s7 \right) \end{aligned}$$

**Spatial distribution alignment across modalities.** To enforce alignment between gene and image representations, we define alignment losses using Maximum Mean Discrepancy (MMD) and contrastive loss:

$$\begin{aligned} {\mathcal{\mathcal{L}}}_{align\_GD}=MMD\left( H^{1,\Theta},H^{2,\Theta} \right)\#\left( s8 \right) \end{aligned}$$

$$\begin{aligned} {\mathcal{\mathcal{L}}}_{align\_SLD}=\frac{1}{N_{\Theta}}\sum_{k=1}^{N} MMD\left( \left( H^{1,\Theta} \right)_{k}^{Nei},\left( H^{2,\Theta} \right)_{k}^{Nei} \right)\#\left( s9 \right) \end{aligned}$$

$$\begin{aligned} {\mathcal{\mathcal{L}}}_{CL}={\left\| H^{1,\Theta}-H^{2,\Theta} \right\|_{F}^{2}}/\left\| H^{1,\Theta}+H^{2,\Theta} \right\|\#\left( s10 \right) \end{aligned}$$

The MMD is computed in a reproducing kernel Hilbert space (RKHS):

$$\begin{aligned} MMD\left( X,Y \right)=\left\| \frac{1}{n}\sum_{i=1}^{n} \Psi\left( x_{i} \right)-\frac{1}{n}\sum_{j=1}^{n} \Psi\left( y_{j} \right) \right\|_{H}^{2}\#\left( s11 \right) \end{aligned}$$

with kernel $\left( x,y \right)=e^{-\frac{\left\| x-y \right\|^{2}}{2}}$. $\left( H^{1,\Theta} \right)_{k}^{Nei}$ and $\left( H^{2,\Theta} \right)_{k}^{Nei}$ denote the representations of spot $k$'s spatial neighbors.

- ${\mathcal{\mathcal{L}}}_{align\_GD}$ aligns global distributions for consistent clustering.
- ${\mathcal{\mathcal{L}}}_{align\_SLD}$ encourages local spatial consistency.
- ${\mathcal{\mathcal{L}}}_{CL}$ regularizes representation drift between modalities.

**Joint Optimization:** The overall loss function for stVGP is:

$$\begin{aligned} {\mathcal{\mathcal{L}}}_{overall\_VGP}=\underset{VGP loss}{\underbrace{(L_{exp}+L_{img})}}+\underset{cross-dimension loss}{\underbrace{\lambda_{1}\left( {\mathcal{\mathcal{L}}}_{CL}+{\mathcal{\mathcal{L}}}_{align_{GD}}+{\mathcal{\mathcal{L}}}_{align_{SLD}} \right)}}\#\left( s12 \right) \end{aligned}$$

where $\lambda_{1}$ is a tunable weight controlling the strength of alignment constraints (default value is 0.01), and is robust to moderate variation as confirmed by sensitivity analysis (Supplementary Fig. 40a-40d and Supplementary Note 1.15).

- 1. **Evaluation of domain detection accuracy used in spatial transcriptomics data.**

We quantitatively evaluated the domain detection accuracy of the results of stVGP and other methods including Leiden^6,7^ (Scanpy version 1.10.1), Louvain^7^ (Scanpy version 1.10.1), BASS^14^ (version 1.1.0.016), BayesSpace^15^ (version 1.14.0), GraphST^10^ (version 1.1.1), STAligner^4^ (version 1.0.0), STitch3D^3^ (version 1.0.3), SCAN-IT(version 0.1)^11^, and SpaceFlow^12^ (version 1.0.3).

**Adjusted Rand Index (ARI)：**Adjusted Rand Index (ARI) is a metric used to assess the consistency between clustering results and true labels. ARI value is between -1 and 1. 1 means perfect agreement and 0 means random agreement. We denote $X=\{X_{1},X_{2},\ldots,X_{n}\}$ and $Y=\{Y_{1},Y_{2},\ldots,Y_{n}\}$ as the clustering result and the true label, respectively. Clustering result $X$ is divided into $r$ categories. The true label $Y$ is divided into $s$ categories. ARI is calculated by:

$$\begin{aligned} ARI=\frac{\sum_{i}^{r} \sum_{j}^{s} \left( \begin{matrix} n_{i,j} \\ 2 \end{matrix} \right)-\frac{\sum_{i}^{r} \left( \begin{matrix} a_{i} \\ 2 \end{matrix} \right)\sum_{j}^{s} \left( \begin{matrix} b_{j} \\ 2 \end{matrix} \right)}{\left( \begin{matrix} n \\ 2 \end{matrix} \right)}}{\frac{\sum_{i}^{r} \left( \begin{matrix} a_{i} \\ 2 \end{matrix} \right)+\sum_{j}^{s} \left( \begin{matrix} b_{j} \\ 2 \end{matrix} \right)}{2}-\frac{\sum_{i}^{r} \left( \begin{matrix} a_{i} \\ 2 \end{matrix} \right)\sum_{j}^{s} \left( \begin{matrix} b_{j} \\ 2 \end{matrix} \right)}{\left( \begin{matrix} n \\ 2 \end{matrix} \right)}}\#\left( s13 \right) \end{aligned}$$

Here, $n_{i,j}$ is the number of clustered pairs that one element of pairs belongs to class $i$ in clustering result and the other belongs to class $j$ in true label. $a_{i}$ is the number of class $i$ in clustering result. $b_{j}$ is the number of class $j$ in true label. $n$ is the length of the clustering result and true label.

**Normalized Mutual Information (NMI):** Normalized Mutual Information (NMI) is a metric used to assess the consistency between clustering results and true labels. NMI value is between -1 and 1. 1 means perfect agreement and 0 means random agreement. We denote $X=\{X_{1},X_{2},\ldots,X_{n}\}$ and $Y=\{Y_{1},Y_{2},\ldots,Y_{n}\}$ as the clustering result and the true label, respectively. Clustering result $X$ is divided into $r$ categories. The true label $Y$ is divided into $s$ categories. NMI is calculated by:

$$\begin{aligned} NMI\left( X,Y \right)=\frac{MI\left( X,Y \right)}{\frac{H\left( X \right)+H\left( Y \right)}{2}}\#\left( s14 \right) \\ MI\left( X,Y \right)=\sum_{i}^{r} \sum_{j}^{s} \frac{\left| X^{i}\cap Y^{j} \right|}{n}\log\frac{\left| X^{i}\cap Y^{j} \right|\times n}{\left| X^{i} \right|\times\left| Y^{j} \right|}\#(s15) \\ H\left( X \right)=-\sum_{i}^{r} \frac{\left| X^{i} \right|}{n}\log\frac{\left| X^{i} \right|}{n}\#(s16) \\ H\left( Y \right)=-\sum_{j}^{s} \frac{\left| Y^{j} \right|}{n}\log\frac{\left| Y^{j} \right|}{n}\#(s17) \end{aligned}$$

Here, MI is the Mutual Information of clustering result and true label. $H()$ is the entropy of clustering result or true label. $X^{i}$ represents class $i$ in clustering result and $\left| X^{i} \right|$ represents the number of class $i$. $Y^{j}$ represents class $j$ in clustering result and $\left| Y^{j} \right|$ represents the number of class $j$. $\left| X^{i}\cap Y^{j} \right|$ represents the number of clustered pairs that one element of pairs belongs to class $i$ in clustering result and the other belongs to class $j$ in true label.

- 1. **Practical application of stVGP across multiple datasets**

To demonstrate its applicability, the stVGP was evaluated on five representative datasets, including the human dorsolateral prefrontal cortex (DLPFC)^16^, whole brain^17^, developing human heart^18^, mouse olfactory bulb (MOB) spatial transcriptomics^19^, and human breast cancer datasets^19^. These datasets were generated using multiple sequencing platforms and exhibited substantial variation in spatial resolution. In addition, they span different species and encompass diverse tissue systems. Despite these differences, stVGP consistently achieved highly accurate predictions, thereby confirming not only its methodological validity but also its robustness across heterogeneous platforms and tissue types. Collectively, these evaluations provide a comprehensive benchmark for assessing stVGP across diverse biological contexts.

The DLPFC dataset consists of four slices (IDs:151673–151676), which exhibit both morphological similarity (151673–151674, 151675–151676) and divergence (151674–151675). For evaluation, stVGP was applied to all slices jointly. Specifically, the data were encoded using a variational Gaussian process autoencoder to correct for batch effects, and the slices were aligned by selecting one slice as the reference and mapping the remaining slices onto it to construct a unified spatial coordinate system. The latent representations were then embedded into this framework, and Gaussian process–based correlations were leveraged to infer gene expression at unsampled locations.

Subsequently, clustering analysis was performed on the latent representations using the R package mclust (version 6.0.0), and the results were visualized in a lower-dimensional UMAP embedding. Model performance was further evaluated by comparing the predicted gene expression with the measured data, and the reconstructed virtual sections were registered into the unified spatial coordinate framework.

The whole-brain dataset comprises 35 slices, representing a relatively large collection. Due to the substantial inter-slice distances, these slices exhibit considerable morphological variation, which poses additional challenges for clustering and alignment. To address this, we adopted a sequential alignment strategy tailored for whole-brain data. Specifically, slices were ordered according to the sequence of tissue collection, with each slice aligned to its immediate predecessor. This approach effectively mitigates the challenges of integrating multi-slice data spanning diverse functional regions. After alignment, all slices were batch-normalized and embedded into latent representations, which were subsequently registered into a unified spatial coordinate system. A Gaussian mixture model was then applied to cluster the latent representations and delineate coherent feature domains. Finally, we reconstructed the three-dimensional distribution and composition of functional domains across the whole-brain dataset, providing an integrated view of their spatial organization. Importantly, the reconstructed domains were consistent with known anatomical structures, underscoring the robustness of stVGP on large, heterogeneous datasets.

Following the whole-brain analysis, we next analyzed the developing heart dataset, which comprises nine slices exhibiting considerable morphological variability and thus posing challenges for alignment and domain reconstruction. To address this, we employed the sequential alignment strategy. All slices were integrated into stVGP for batch correction and 3D registration, and we subsequently evaluated its performance in predicting gene expression. Specifically, each slice was sequentially masked, and its expression was inferred from the remaining slices; the predicted patterns of three representative marker genes were then compared with their measured expression profiles. The predictions closely recapitulated the spatial expression patterns of these marker genes, highlighting stVGP’s robustness in handling morphologically heterogeneous cardiac tissue and its utility in studying dynamic developmental processes.

For the MOB dataset, the original study collected twelve slices from different individuals, of which we selected the last three for analysis. Because no additional manual annotations were available, our functional domain analysis could not be directly compared with the results in the original report. Instead, we focused on performing trajectory analysis of the tissue domains inferred by stVGP. We analyzed three MOB slices with stVGP, using a reference-based alignment strategy in which one slice was designated as the reference and the remaining slices were aligned to it. After obtaining the MOB embeddings, we used the Python toolkit Scanpy to perform clustering across all methods and delineate the distributions of functional domains. We then performed additional dimensionality reduction to analyze cellular trajectory changes inferred by stVGP. Finally, we evaluated the generative capability of stVGP through masking strategies and validation against MOB marker genes.

The final dataset analyzed by stVGP was the human breast cancer dataset, which comprises four sections. These sections exhibit substantial rotational variation, leading to pronounced spatial discrepancies across them. Such variability poses a particular challenge for the alignment task. Here, given the limited number of slices, we employed the reference-based alignment strategy. The four slices were subsequently integrated into stVGP for batch correction, latent representation extraction, construction of a 3D coordinate framework, and masked prediction of gene expression. Here, we focused on evaluating the predictive capability of stVGP. By leveraging features extracted from tissue images, we selected spatially informative genes and specifically compared the discrepancies between predicted and measured expression levels.

- 1. **Model architecture and parameterization of stVGP**

The stVGP is a variational spatial Gaussian process framework designed to integrate multi-modal, multi-slice spatial transcriptomics (ST) data for coherent 3D tissue reconstruction.

First, a variational Gaussian process autoencoder is employed for batch correction and the latent embedding of gene expression. Key parameters such as the latent dimension and learning rate can be specified by the user. If not explicitly defined, the dropout rate defaults to 0.1 and the weight decay defaults to 1e-4. The activation function defaults to LeakyReLU, and the optimizer defaults to Adam.

Second, a hybrid alignment module integrates rigid and non-rigid registration to generate aligned coordinates. The weighting ratio between rigid and non-rigid components is adjustable, along with parameters such as the number of spatially specific genes for rigid alignment and the choice of spatial anchors.

Finally, a Gaussian process decoder is applied to reconstruct gene expression in unsampled regions. In this module, parameters such as the Gaussian process kernel (covariance function) are available for tuning.

- 1. **Description of datasets**

The 10x Visium human DLPFC dataset consists of four adjacent tissue slices (151673–151676). The original study provides manual annotations, including white matter (WM) and six neocortical layers (L1–L6), which we used as the ground truth for accuracy evaluation. This dataset contains the largest number of spots, with approximately 3,500 spots per slice, and a shared gene set of 33,538 genes. From each slice, we selected the top 5,000 highly variable genes and subsequently calculated their intersection.

The adult mouse whole brain dataset comprises 35 tissue slices, with spot counts ranging from 152 to 620 and totaling 17,088 spots. These slices share a gene set of 23,371 genes. We selected the 8,000 highly variable genes from each slice and computed their intersection before inputting the data into stVGP.

The developing human heart dataset contains nine slices, each with 100 to 212 spots, and a common gene set of 39,525 genes. Preprocessing was performed in a manner similar to that of the whole-brain dataset, after which the processed data were input into stVGP.

The mouse olfactory bulb (MOB) dataset comprises three individual slices, each containing 262–282 spots and 16,034–16,416 genes. Because the gene sets across slices were not completely identical, we first calculated their intersection. We then applied Scanpy to filter out low-quality spots, and subsequently input all slice data into stVGP as well as comparative methods.

Finally, the human breast cancer dataset consists of four slices, containing 254, 251, 264, and 262 spots, and 14,789–14,929 genes. Data preprocessing followed the same procedure as for the MOB dataset, involving intersection of gene sets across slices prior to input into stVGP.

- 1. **Benchmarking stVGP against SpaGCN for the task of spatial domain identification.**

We benchmarked stVGP against SpaGCN^20^ for the task of spatial domain identification. Utilizing all slices from the DLPFC dataset, we performed a quantitative evaluation based on the Adjusted Rand Index (ARI). The results demonstrate that stVGP not only accurately delineated spatial domains but also achieved superior performance metrics, maintaining a minimum ARI exceeding 0.60 across all four slices (Supplementary Fig. 12). This comparative analysis clearly underscores the efficacy of stVGP in capturing complex spatial tissue architectures.

In comparison to SpaGCN, stVGP's key differentiator lies in its ability to overcome the limitations associated with multi-slice analysis. By integrating multiple slices for spatial consensus domain analysis and facilitating 3D gene reconstruction, stVGP enables a comprehensive framework for multi-modal and multi-slice prediction.

- 1. **Benchmarking of spatial reconstruction methods.**

To comprehensively evaluate stVGP's reconstruction capability, we have supplemented the analysis with additional performance metrics and included a simple interpolation baseline for comparative benchmarking. Specifically, we benchmarked stVGP against this baseline alongside four established methods: GPSA, STitch3D, Tangram^21^, and CellTrek^22^. For this evaluation, we utilized the DLPFC dataset, selecting the White Matter (WM) marker gene, *MOBP*, as the primary target. *MOBP* not only serves as a canonical marker for critical layers but also exhibits distinct spatial specificity, making it an excellent candidate for assessing reconstruction fidelity. To facilitate a fair comparison with interpolation, we focused on the intermediate slices (151674 and 151675) from the DLPFC data. All reported performance metrics represent the average values calculated across these two slices.

We quantitatively assessed the reconstruction accuracy of the *MOBP* gene. Specifically, we calculated the Pearson correlation coefficient (PCC) and Root Mean Square Error (RMSE) between the reconstructed expression of all methods and the ground truth across the two slices (Supplementary Fig. 16a). Among all results, stVGP achieved the highest correlation coefficient and the lowest RMSE, indicating that it demonstrates superior reconstruction performance in quantitative metrics (Supplementary Fig. 16b).

Finally, we analyzed the spatial distribution of the *MOBP* gene. By directly comparing the results with actual expression patterns (Supplementary Fig. 16c-16d), we clearly observed stVGP's generative capability: it effectively preserves biological information while minimizing noise from the surrounding background (Supplementary Fig. 16c-16d). These findings collectively demonstrate that stVGP is the optimal method for gene expression reconstruction.

- 1. **Differences between stVGP spatial reconstruction and single-cell spatial localization reconstruction.**

We have expanded our literature review and conducted a comprehensive comparative analysis to better illustrate stVGP's performance in both reconstruction and cross-modal fusion. Specifically, Tangram and CellTrek are representative mapping-based approaches that align single-cell data to spatial coordinates, thereby enabling gene expression reconstruction. Accordingly, we benchmarked stVGP against these methods to evaluate gene reconstruction capabilities.

For this evaluation, we selected the DLPFC dataset, specifically utilizing the intermediate sections (151674 and 151675) to ensure representative sampling and minimize potential boundary effects. We focused on the White Matter (WM) marker gene, *MOBP*, as our primary validation target. *MOBP* was chosen for its distinct spatial clustering patterns and strict layer specificity, making it an optimal candidate for assessing reconstruction fidelity. Qualitatively, we visualized the reconstruction of *MOBP* by stVGP, Tangram, and CellTrek, comparing them against the ground truth (Supplementary Fig. 17a). stVGP accurately captured the gene's spatial clustering patterns and faithfully reproduced its global expression profile. In contrast, while Tangram and CellTrek successfully detected the general expression trend, they exhibited signs of over-smoothing (or over-correction), which compromised the fidelity of the fine-grained spatial details (Supplementary Fig. 17a).

Quantitatively, we calculated the correlation coefficients and RMSE between the predicted and ground truth expression across the two slices (Supplementary Fig. 17b-17d). stVGP achieved the best performance in terms of both correlation and RMSE (Supplementary Fig. 17b and 17c). Regarding structural similarity, although stVGP did not achieve the absolute highest SSIM index, its performance remained highly competitive, comparable to that of the top-performing method, CellTrek (Supplementary Fig. 17d).

Finally, to provide a holistic comparison, we evaluated computational efficiency. The results demonstrate that stVGP offers the most efficient runtime among the tested methods (Supplementary Fig. 17e). Collectively, considering reconstruction accuracy, structural fidelity, and computational efficiency, stVGP emerges as the most robust and suitable approach for spatial reconstruction (Supplementary Fig. 17e).

- 1. **Benchmarking for computational efficiency of spatial reconstruction methods.**

To further illustrate the spatial reconstruction capabilities of stVGP, we have included additional gene reconstruction comparisons. Specifically, we focused on the *MOBP* gene within the DLPFC dataset. We selected *MOBP* as a representative validation target because it not only exhibits a distinct spatial pattern but also serves as a canonical marker for the white matter (WM) layer. We benchmarked our method against four established approaches for spatial expression reconstruction: GPSA, STitch3D, Tangram and CellTrek. Among these, GPSA and STitch3D perform direct spatial reconstruction, whereas Tangram and CellTrek infer virtual spatial expression profiles via single-cell localization. Additionally, to rigorously evaluate stVGP's capacity to capture spatial gene distributions, we included direct slice interpolation as a baseline for comparison. In results, stVGP demonstrates solid performance in both computational efficiency and accuracy (Supplementary Fig. 18a-18d).

First, we evaluated the runtime of all methods. With the exception of the interpolation method, stVGP achieved the fastest execution time (Supplementary Fig. 18a). Additionally, we quantified resource consumption, where stVGP exhibited the lowest total peak memory footprint (CPU + GPU) (Supplementary Fig. 18b). We also examined the peak utilization of all methods on both CPU and GPU (Supplementary Fig. 18c and 18d). The results demonstrate that stVGP maintains a highly efficient memory footprint. Overall, these findings confirms that stVGP does not consume excessive computational resources.

- 1. **Benchmarking batch effect integration and biological conservation across multiple methods.**

Here, we benchmarked stVGP against five established methods: STitch3D, STAligner, MENDER^23^, Harmony^24^, and DeepST^25^. We analyzed slice data spanning three developmental stages of the human heart. Dimensionality reduction visualization of the raw data revealed three distinct clusters corresponding to these stages, indicating strong batch effects.

Subsequently, we applied stVGP and the comparative methods to address these discrepancies. The results demonstrate that stVGP successfully identified and harmonized batch effects across all three periods, effectively integrating the data based on biological signals (Supplementary Fig. 29). In contrast, other methods failed to adequately remove these variations, showing residual batch effects (Supplementary Fig. 29). To quantitatively evaluate this performance, we assessed the Batch Average Silhouette Width (bASW)^26^. stVGP achieved the highest bASW score (bASW = 0.914), verifying its superior capability and robustness in batch effect removal (Supplementary Fig. 29).

- 1. **Spatial trajectory inference and molecular dynamics in the developing human heart.**

We performed a comprehensive spatial trajectory analysis on the integrated dataset spanning 4.5-9 PCW (Supplementary Fig. 30)^18,27^. When mapping the inferred pseudotime back to the physical time points, we observe a clear temporal stratification (Supplementary Fig. 30a) that mirrors the actual developmental timeline: the 4.5–5 PCW slices are predominantly occupied by 'early' pseudotime states (represented in dark purple in Supplementary Fig. 30b, whereas the 9 PCW slices are significantly enriched with 'late' pseudotime states (yellow/green). This gradient confirms that the batch correction did not compress the temporal axis.

Furthermore, we validated that this trajectory is driven by biologically relevant molecular programs rather than technical artifacts. As shown in the gene trend analysis (Supplementary Fig. 30d-30g), we identified specific gene modules that exhibit monotonic changes along the inferred pseudotime. For instance, *SLIT3*, a marker associated with structural maturation, shows a consistent upregulation along the trajectory and is spatially enriched in the 9 PCW sections (Supplementary Fig. 30e). Conversely, *MYL2* exhibits high expression in the early pseudotime phases and is spatially predominant in the 4.5-5 PCW sections (Supplementary Fig. 30f). The precise alignment between these molecular dynamics, the inferred pseudotime, and the physical developmental stages confirms that stVGP successfully corrects technical misalignment to allow comparison, yet strictly preserves the underlying biological progression associated with tissue maturation.

- 1. **Characterization of spatial domains and developmental trajectories in the developing human heart.**

Leveraging stVGP's capacity to characterize spatial features, we analyzed the spatial dynamics of gene expression and successfully reconstructed tissue developmental trajectories^18,27^. Here, we selected the developing heart dataset. This dataset encompasses three developmental stages: 4.5-5, 6.5, and 9 post-conception weeks (PCWs). We conducted biological interpretation and developmental trajectory studies on this dataset.

To validate the biological interpretability of the spatial domains identified by stVGP, we performed a comprehensive differential expression analysis on the human heart dataset at late developmental stages (6.5 and 9 PCW). We first examined the top four marker genes for each of the ten identified domains (Supplementary Fig. 31a). The expression signatures of these domains remained highly conserved across both developmental time points, suggesting that stVGP captures robust and biologically stable spatial structures (Supplementary Fig. 31a). We then characterized the functional identity of specific domains based on their unique gene signatures (Supplementary Fig. 31b). For instance, Domain 0 was defined by the upregulation of *MYH7*, *NPPA*, and *MYL2*, consistent with the molecular profile of trabecular ventricular myocardium. Domain 1 exhibited high expression of *OGN* and *DCN*, identifying it as the outflow tract and large vessel region. Conversely, Domain 8 was distinguished by *MYH6* expression, marking it as atrial myocardium.

To further investigate the functional role of the trabecular ventricular myocardium (Domain 0), we performed functional enrichment analysis on its specific marker genes (Supplementary Fig. 31c). Pathway enrichment analysis revealed that this domain is strongly associated with cardiac contraction-related programs (Supplementary Fig. 31c). Multiple hierarchically related pathways, including 'cardiac muscle contraction', 'myofibril assembly', and 'sarcomere organization', were significantly enriched, validating the ability of stVGP to correctly delineate functional tissue regions.

Leveraging stVGP's capacity to integrate spatial and molecular information, we reconstructed the developmental trajectory of the cardiac tissue. The trajectory inference revealed a progression from early trabecular myocardium towards an oxidative-metabolism-enriched phenotype (Supplementary Fig. 31d). Notably, projecting these pseudotime values back onto the tissue coordinates (Supplementary Fig. 31e) unveiled a clear spatial gradient that mirrors the anatomical maturation of the heart.

We further dissected the molecular drivers of this trajectory by analyzing gene expression dynamics along the pseudotime (Supplementary Fig. 31f). We specifically highlighted two distinct patterns: *FOSB*, a regulator of cell proliferation and differentiation, showed a monotonic increase along the trajectory, whereas *MYL2* exhibited a declining trend (Supplementary Fig. 31g). Crucially, the spatial visualization of these genes (Supplementary Fig. 31h) corroborated the pseudotime analysis: *FOSB* was highly enriched in the spatially 'late' developmental regions, while *MYL2* was localized to the 'early' regions. This congruence between pseudotime dynamics and spatial expression patterns confirms that stVGP can effectively capture the spatial logic of tissue development.

- 1. **Quantitative evaluation of cross-slice gene expression prediction performance.**

To provide a more comprehensive assessment, we have incorporated additional evaluation metrics and expanded our gene visualization comparisons. Specifically, we included Pearson and Spearman correlation coefficients to quantify expression trends, along with the Structural Similarity Index (SSIM) to evaluate spatial structural fidelity. Furthermore, to strictly measure reconstruction accuracy, we calculated the Root Mean Square Error (RMSE) between the predicted and ground truth expression. Regarding visualization, we supplemented the analysis with the expression patterns of structure-specific genes, such as *SPARC* and *PRSS23*, to further validate stVGP's predictive capabilities.

First, we conducted a cross-modal quantitative evaluation to assess the similarity between the predicted and ground truth gene expression (Supplementary Fig. 39a and 39b). We initially computed Pearson's and Spearman's correlation coefficients. The results demonstrate a high correlation; for instance, both coefficients for *FN1* exceeded 0.64, and for *COL3A1* exceeded 0.56 (Supplementary Fig. 39a and 39b).

Subsequently, we employed the Structural Similarity Index (SSIM), with all genes achieving values exceeding 0.7 (Supplementary Fig. 39c). This indicates that the spatial distribution of our predicted genes exhibits high structural fidelity to the true distribution. To further quantify precision, we measured the Root Mean Square Error (RMSE), which remained below 1 (in count units) for all genes (Supplementary Fig. 39d).

Qualitatively, we evaluated the spatial distribution of *SPARC* and *PRSS23* across four tissue slices (Supplementary Fig. 39e). Compared to the observed expression, stVGP accurately localized gene clusters, effectively reducing noise while preserving key distributional characteristics (Supplementary Fig. 39e).

Furthermore, we acknowledge certain limitations within the stVGP cross-modal prediction module. Its performance is heavily contingent on the alignment of hidden layer distributions. Despite mapping data into a shared latent space, the inherent heterogeneity between modalities continues to challenge prediction accuracy. Consequently, the efficacy of this distribution alignment remains a critical determinant of model performance. Currently, our approach primarily relies on distance-based metrics within the same space for alignment. This may overlook subtle feature-specific discrepancies, potentially leading to suboptimal representations. Moving forward, we plan to investigate advanced distribution alignment strategies to develop a more robust cross-modal prediction framework.

- 1. **Sensitivity analysis of stVGP performance to hyperparameter variations.**

Specifically, we utilized the Adjusted Rand Index (ARI) to assess the robustness of the identified spatial domains under different parameter settings on DLPFC dataset. Furthermore, we validated the reconstruction of the WM marker gene *MOBP*, providing additional evidence of the model's stability against parameter variations. Across all parameter settings, the ARI values consistently remained above 0.55 (10 independent runs per setting) (Supplementary Fig. 40a), despite minor fluctuations. Furthermore, in the reconstruction of the *MOBP* marker gene, stVGP demonstrated a high correlation with the actual expression levels: Pearson Correlation Coefficient > 0.65 (Supplementary Fig. 40b), Spearman's Rank Correlation Coefficient > 0.5 (Supplementary Fig. 40c), and Kendall's Rank Correlation Coefficient > 0.4 (Supplementary Fig. 40d). This stable performance highlights the robustness of the stVGP framework.

- 1. **Evaluation of stVGP robustness under various data perturbations.**

To better characterize the robustness of stVGP, we conducted additional tests across different biological scenarios. Specifically, we validated stVGP's performance across different spatial resolutions and missing slice scenarios, while also assessing its robustness against data sparsity and varying levels of noise.

First, we evaluated stVGP's performance across different spatial resolutions using the DLPFC dataset, calculating the average performance across all tissue slices. The results demonstrate that stVGP maintains remarkable stability under varying resolutions. Specifically, at Merge 2 resolution (50% spot reduction), stVGP achieved an ARI of 0.633, showing only a negligible decline compared to the full-resolution data (Supplementary Fig. 41a). Even under a substantial 75% reduction in spot density (Merge 4), stVGP maintained an ARI exceeding 0.55 (Supplementary Fig. 41a). This moderate performance attenuation is attributed not only to the shift in data distribution but also to the information loss inherent to the synthetic downsampling process. Collectively, these findings underscore the robust capabilities of stVGP.

Subsequently, we proceeded to test the model under conditions of slice loss. We constructed scenarios where slices 151673 to 151676 were sequentially discarded, and calculated stVGP's performance for each case (Supplementary Fig. 41b). In all scenarios, the ARI metric remained robust, staying above 0.55 (Supplementary Fig. 41b). Most notably, when slice 151675 was missing, the ARI reached 0.636, demonstrating almost no performance degradation (Supplementary Fig. 41b). Even in the most challenging scenario - where slice 151674 was missing - stVGP still achieved an ARI of 0.562 (Supplementary Fig. 41b). We attribute this specific decline to the fact that slice 151674 exhibited the highest accuracy in the original dataset; therefore, its absence significantly weighed down the overall average.

Additionally, stVGP demonstrated exceptional robustness against varying levels of noise interference and data sparsity. While accuracy naturally showed a gradual decline as noise and sparsity intensities increased, the ARI metric consistently remained above 0.4 across all tested scenarios (Supplementary Fig. 41c and 41d). In the sparse data impact test, we introduced artificial sparsity by randomly masking individual gene expression entries with a fixed probability (The original data sparsity was 0.7942). Notably, under conditions of minor perturbations (sparsity levels between 0.1 and 0.3), the model exhibited negligible degradation, maintaining highly stable performance (Supplementary Fig. 41c). Similarly, even under elevated noise levels ranging from 0.25 to 0.75, stVGP's ARI consistently exceeded 0.5 (Supplementary Fig. 41d). Overall, stVGP can overcome the influence of different data types and demonstrates a high level of performance.

- 1. **Impact of fine-tuning strategy on spatial domain identification and gene expression reconstruction.**

In the context of domain recognition on the DLPFC dataset, the absence of fine-tuning resulted in a tangible decline in clustering performance (Supplementary Fig. 42a and 42b). Quantitatively, the Adjusted Rand Index (ARI) decreased from 0.638 to 0.566 (Supplementary Fig. 42c), and the Normalized Mutual Information (NMI) dropped from 0.725 to 0.651 (Supplementary Fig. 42d). Beyond these numerical metrics, visual inspection revealed that spatial domain continuity deteriorated significantly, leading to fragmented regions that failed to accurately delineate fine-grained tissue boundaries. Furthermore, regarding cross-modal prediction on the human breast cancer dataset, the ablation of fine-tuning negatively impacted the generative capabilities of the model, resulting in an approximate 5% decrease in prediction accuracy. While stVGP retained the ability to capture general spatial patterns, the pixel-level precision required for gene recovery was compromised (Supplementary Fig. 42e and 42f). Given the high density of spots inherent to spatial transcriptomics, even a 5% reduction implies a substantial cumulative loss of biological fidelity across the tissue (Supplementary Fig. 42f). Collectively, these results confirm that fine-tuning the visual backbone is indispensable for stVGP to achieve both high-coherence domain recognition and precise cross-modal prediction.

- 1. **Scalability analysis of stVGP across varying dataset sizes.**

In our scalability analysis, we subsampled the dataset, incrementally increasing the size from 1,000 to 17,000 spots. We systematically evaluated stVGP's runtime alongside aggregate peak memory utilization (CPU and GPU). The results demonstrate the efficacy of the 'neighbor-driven' strategy: stVGP achieved an execution time of merely 251 seconds even when processing 17,000 spots (Supplementary Fig. 43a). Furthermore, memory consumption exhibited a linear and controllable increase as the dataset size grew (Supplementary Fig. 43b and 43c). Consequently, these findings confirm that the neighbor-driven strategy significantly enhances stVGP's execution speed, scalability, and computational efficiency.

- 1. **Ablation study of spatial alignment components on the mouse brain dataset.**

We selected the adult mouse brain dataset for this analysis, as it represents the most challenging alignment scenario due to its high volume of slices.

Qualitative evaluation demonstrated that stVGP's hybrid strategy achieves optimal results. Specifically:

Rigid alignment only: Successfully reconstructed the global 3D structure but struggled to perfectly align local tissue details (Supplementary Fig. 44a).

STN alignment only: Achieved local proximity between slices but failed to maintain global structural integrity, resulting in a loss of overall shape (Supplementary Fig. 44a).

Hybrid strategy: Effectively integrated the strengths of both, capturing the global framework while precisely aligning local morphological details (Supplementary Fig. 44a).

Quantitative evaluation further confirmed these findings. We calculated the alignment distance and alignment score for all adjacent slices (Supplementary Fig. 44b). Results showed that the hybrid strategy yielded the best performance metrics, whereas relying on a single strategy led to performance degradation—most notably with STN alone (Supplementary Fig. 44c). The statistics for mean and median alignment scores followed the same pattern (Supplementary Fig. 44d).

In conclusion, the hybrid strategy is the optimal choice. Rigid alignment provides the fundamental structural basis (primary performance), while STN alignment acts as a crucial supplementary step for refining local details (Supplementary Fig. 44).

- 1. **stVGP robustness, ablation studies, and parameter sensitivity.**

We systematically evaluated how the clustering accuracy of stVGP responds to different data conditions, including varying spot resolutions, missing slices, different sparsity levels, and diverse noise. Across all settings, stVGP maintained high accuracy, demonstrating strong robustness (Supplementary Fig. 41a–d and Supplementary Note 1.16).

We further examined the effect of fine-tuning the ResNet-50 backbone on cross modal performance. The results showed that while stVGP's performance without fine-tuning exhibited some fluctuations, it remained generally stable and robust (Supplementary Fig. 42a-42f and Supplementary Note 1.17).

Because Gaussian processes (GPs) naively scale as O(*N*³) with the number of observations, stVGP adopts a neighbor-driven sampling strategy that restricts computations to a local neighborhood (by default, the 20 nearest neighbors), achieving a favorable balance between accuracy and efficiency. To assess scalability, we quantified the computational cost of stVGP across increasing dataset sizes (Supplementary Fig. 43a–c and Supplementary Note 1.18).

Finally, we performed ablation experiments on the alignment module to evaluate the contributions of different alignment strategies, further clarifying their roles in the overall performance of stVGP (Supplementary Fig. 44a–d and Supplementary Note 1.19).

- 1. **Data preprocessing and domain detection metric evaluation.**

**Data preprocessing:** stVGP takes ST gene expression, spatial coordinate information, and histological image of multiple ST slices as input. Before all analyses, for each slice we normalize the raw gene expressions according to library size and log-transformed using the Scanpy package. Then, we select the top highly variable genes. We take the intersection of the highly variable genes of all the input slices to ensure the spot features are shared across slices.

**Clustering:** The spatial domains were identified by clustering algorithm with the embeddings from stVGP as input. For datasets with prior background about the number of domain labels, the mclust clustering algorithm^28^ implemented by R package mclust and Gaussian Mixture Model (GMM) implemented by Python package sklearn were used. For datasets without background, the Louvain algorithm implemented by Python package Scanpy was used.

**Evaluation metrics:** We used alignment score, alignment distance, Adjusted Rand Index (ARI), and normalized mutual information (NMI) to evaluate stVGP and other methods. We also used Pearson correlation coefficient (PCC) to evaluate the reconstruction slices accuracy. Specific calculations of these evaluation metrics are described in the Supplementary Note 1.3.

**Data description:** The 10x Visium human DLPFC dataset was consists of four adjacent tissue (151673-151676) slices. The manually annotated labels in the original study include white matter (WM) and six neocortical layers (L1-L6). We used these annotations as ground truth to evaluate the performance of simultaneous spatial domain identification and the quality of batch-corrected embeddings. Each slice contains ~3,500 spots, ranging from 3,460 to 3,673. They have the common gene number 33,538. The adult mouse brain dataset consisted of 35 tissue slices. The number of spots in these slices ranged from 152 to 620, totaling 17,088 spots. Also, they had a common gene set with a number of 23,371. The developing human heart dataset has 9 slices. The number of spots in these slices ranged from 100 to 212. They had a common gene set with a number of 39525. MOB dataset has three slices and the number of spots in MOB dataset ranged from 262 to 282. Also, the number of genes in these three slices were 16416, 16218, and 16034 respectively. Finally, human breast cancer dataset has four slices with 254, 251, 264, 262 spots and 14880,14789,14929,14808 genes respectively.

**Section S2. Supplementary Figures and Supplementary Tables：**

**Supplementary Figure 1**. **Applications of stVGP.** stVGP supports diverse downstream analyses, including virtual slice generation, functional structure decoding, coherent domain identification, trajectory inference, and cross-modal prediction.

**
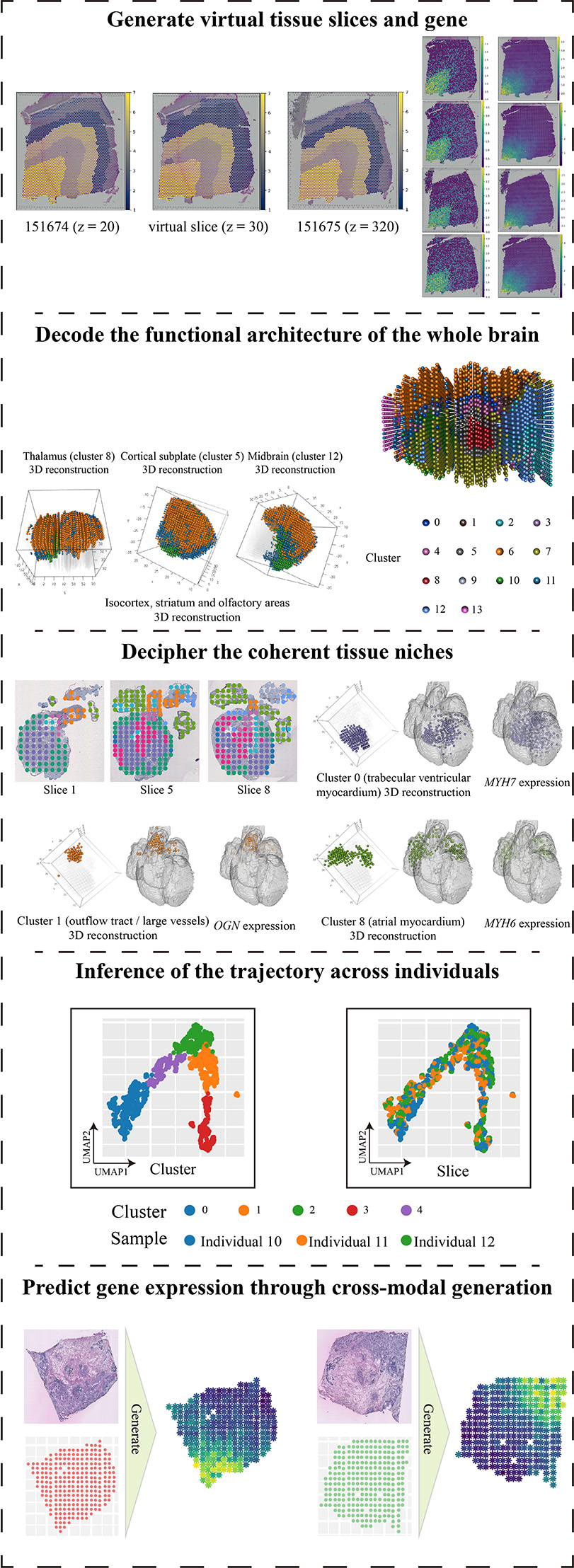
**

**Supplementary Figure 2**. **Overview of stVGP.** stVGP is a variational spatial Gaussian process framework designed to integrate multi-modal, multi-slice spatial transcriptomics (ST) data for coherent 3D tissue reconstruction. It jointly models spatial alignment, domain identification, batch effect correction, and gene expression interpolation across slices. **a.** Reconstructing coherent domains from multi-modal multi-slice spatial transcriptomics. The inputs to stVGP are a set of 2D ST slices, each consisting of gene expression matrices, histological images and spatial coordinates. During the analysis process, stVGP gradually completes batch correction, domain identification, and three-dimensional alignment. After completing the above analysis, stVGP also performs virtual slice generation and calculates gene expression in unsampled slices. **b.** Multi-modal variational Gaussian process autoencoder. Here, stVGP performs domain identification and batch correction, and aligns spatial gene expression patterns with histological image features. In the latent space, stVGP utilizes Gaussian processes for more precise analysis and enhanced performance. **c.** Hybrid alignment with spatial transformers and attention fusion. Here, stVGP performs non-rigid alignment using a spatial transformer and fuses this with rigid alignment using an attention mechanism. **d.** Variational Gaussian process decoder for virtual slice generation. Here, stVGP first registers all slices into a unified 3D coordinate system. Then, stVGP uses the mapping relationship to find the 3D spatial network. Subsequently, stVGP generates a new expression embedding based on the 3D spatial network and Gaussian process. Through decoding, stVGP obtains the final gene expression. **e.** Applications of stVGP. stVGP supports diverse downstream analyses, including virtual slice generation, functional structure decoding, coherent domain identification, trajectory inference, and cross-modal prediction.

**
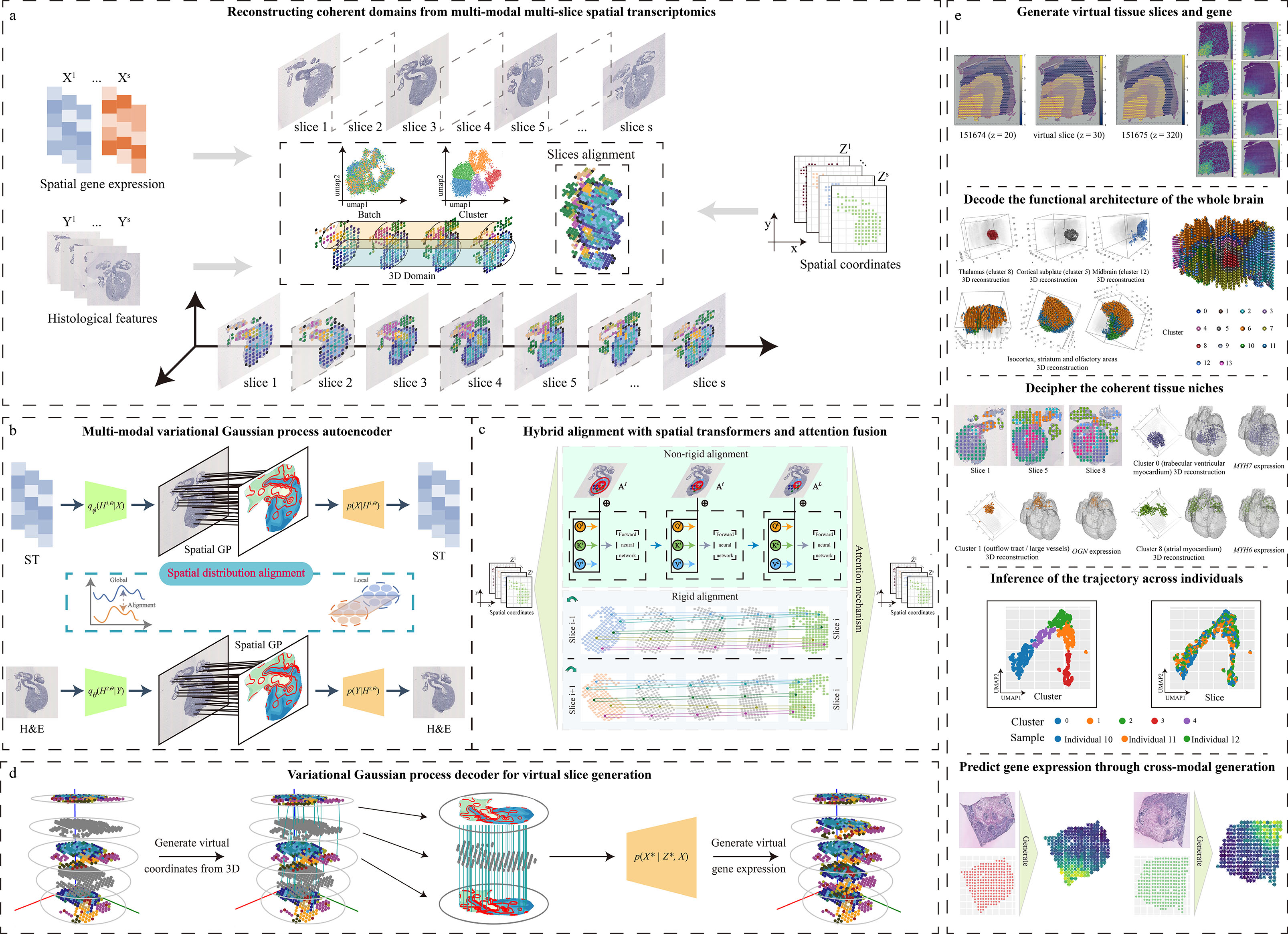
**

**Supplementary Figure 3**. **Comparison of spatial alignment methods on human dorsolateral prefrontal cortex (DLPFC) dataset^16^.** The manual annotations of 10x Visium DLPFC slice 151673, 151674, 151675 and 151676 are shown based on the spatial coordinates. The annotations include cortical layers L1–L6 (Layer 1 to Layer 6) and white matter (WM)^16^. This figure presents the spatial layout of the four slices before alignment and after alignment using six methods: stVGP, PASTE, PASTE2, STitch3D, STAligner, and GPSA.


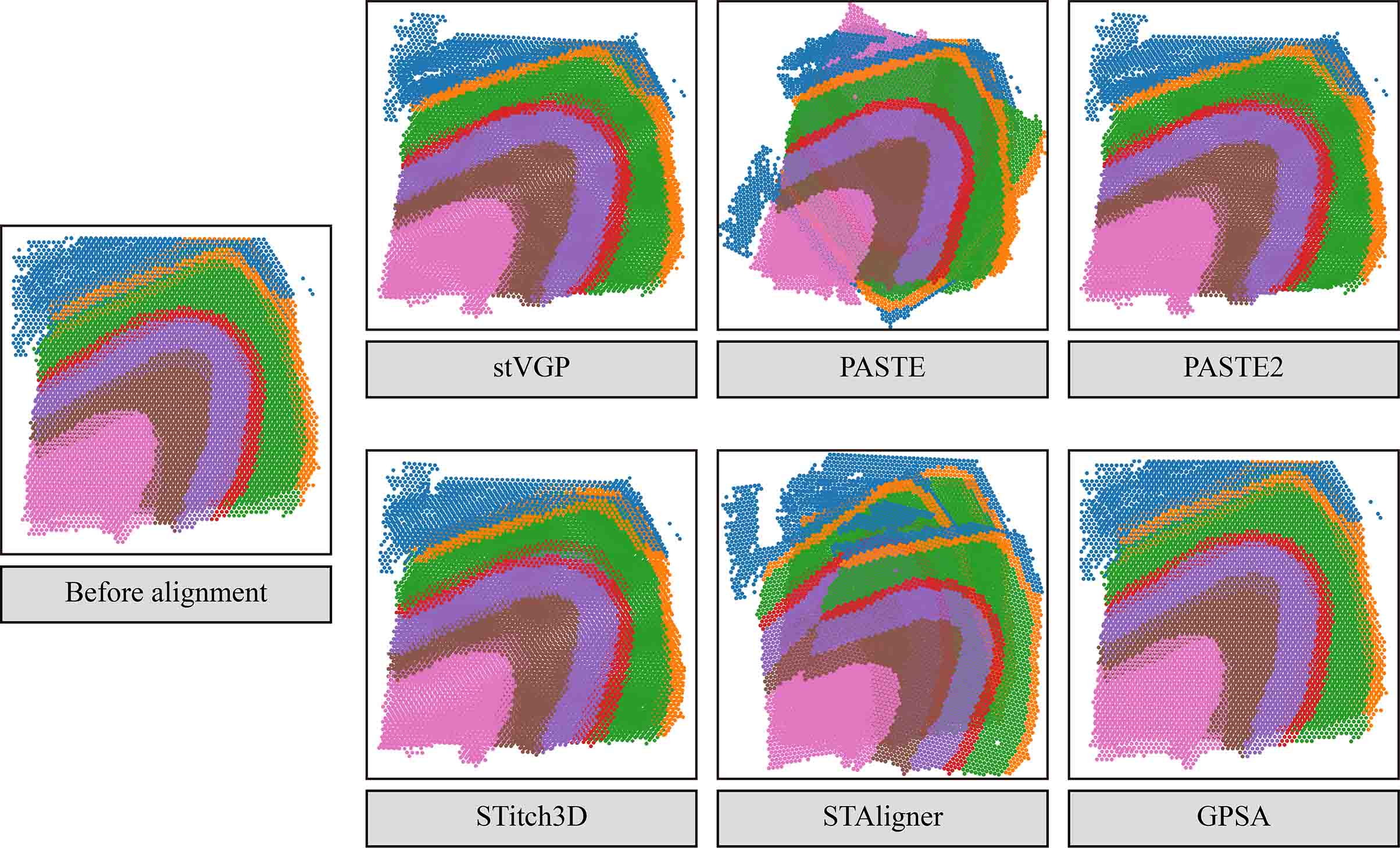


**Supplementary Figure 4**. **Alignment performance of stVGP and comparison with six other alignment methods. a.** Spatial alignment of four slices 151673-151676 using stVGP. **b.** Alignment scores of six alignment methods for three pairs of adjacent slices derived from the four slices. A higher alignment score indicates that the method was more effective. Computational methods included stVGP, PASTE, PASTE2, STitch3D, STAligner, and GPSA. stVGP achieved the best results in the vast majority of pairs. **c.** Box plots of alignment distances for three pairs of adjacent slices derived from the four slices. Each box plot ranges from the first to the third quartiles with the median as the horizontal line, while whiskers represent 1.5 times the interquartile range from the lower and upper bounds of the box. The lower the value of alignment distances, the more reliable the method is.


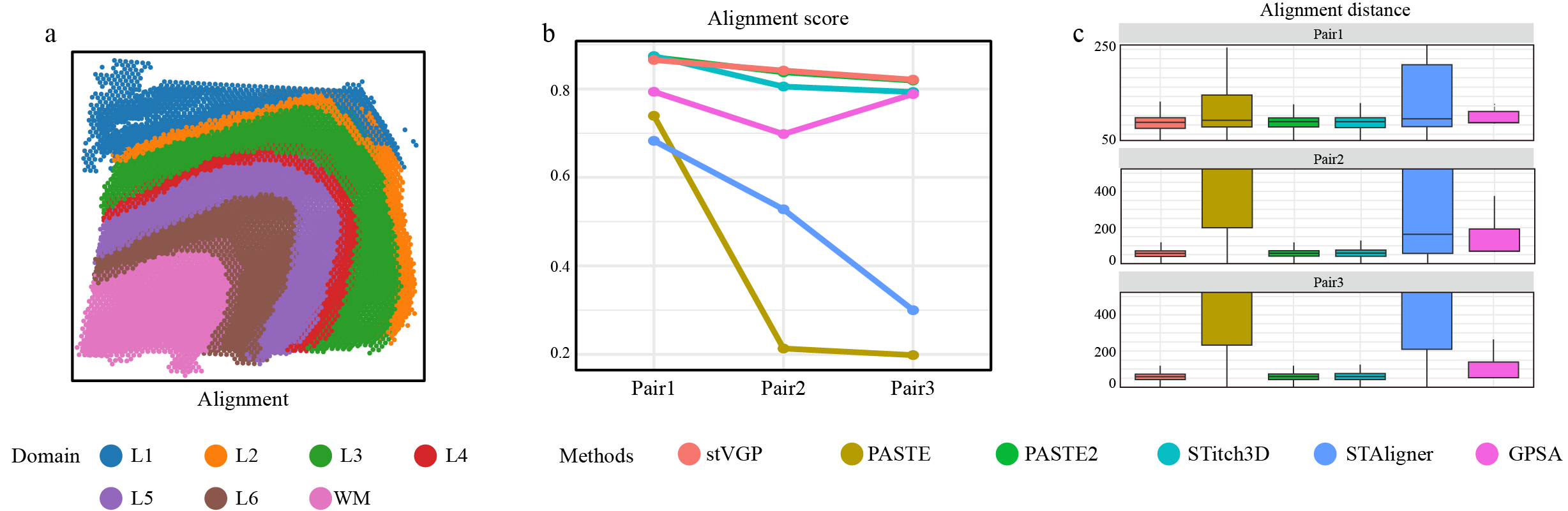


**Supplementary Figure 5**. **Comparison of spatial domain detection methods on DLPFC slice 151674.** Manual region annotations of slice 151674 are shown alongside spatial domain detection results generated by various computational methods. The evaluated methods include stVGP, Leiden, Louvain, BASS, BayesSpace, GraphST, STAligner, STitch3D, SCAN-IT, and SpaceFlow. This comparison highlights the ability of each method to recover anatomically meaningful spatial domains as annotated in the reference.


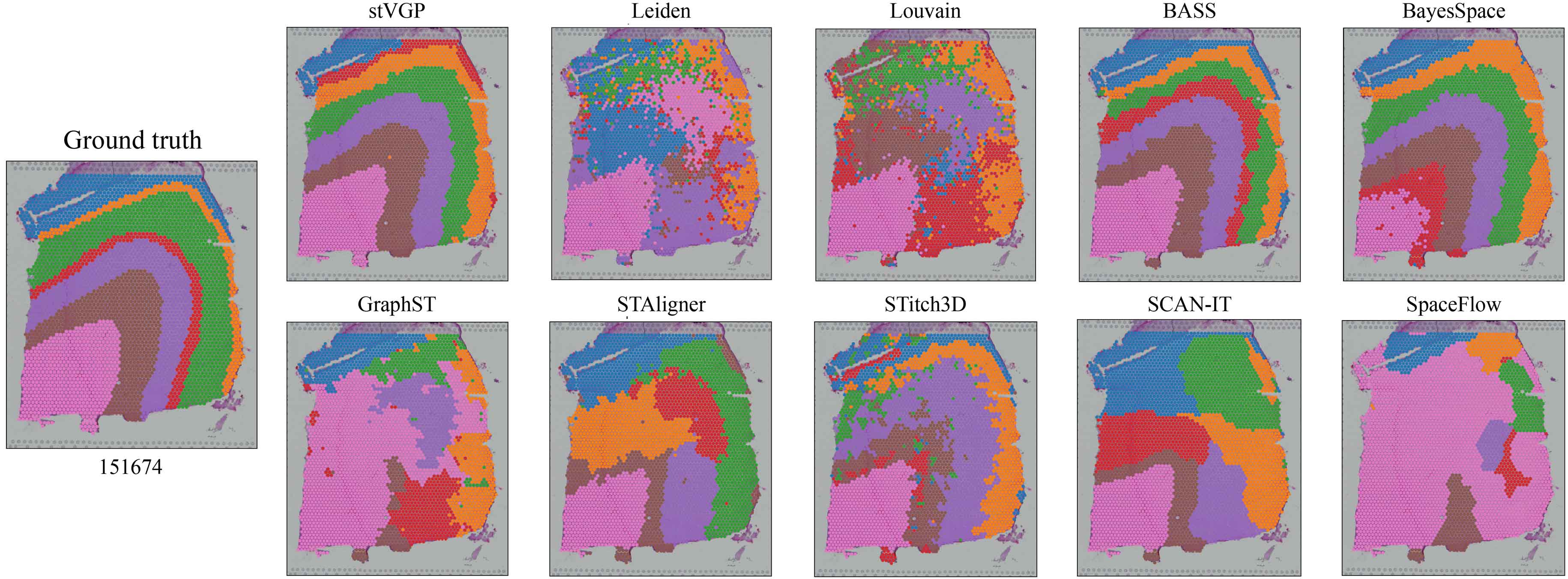


**Supplementary Figure 6**. **Comparison of spatial domain detection methods on DLPFC slice 151675.** Manual region annotations of slice 151675 are shown alongside spatial domain detection results generated by various computational methods. The evaluated methods include stVGP, Leiden, Louvain, BASS, BayesSpace, GraphST, STAligner, STitch3D, SCAN-IT, and SpaceFlow.


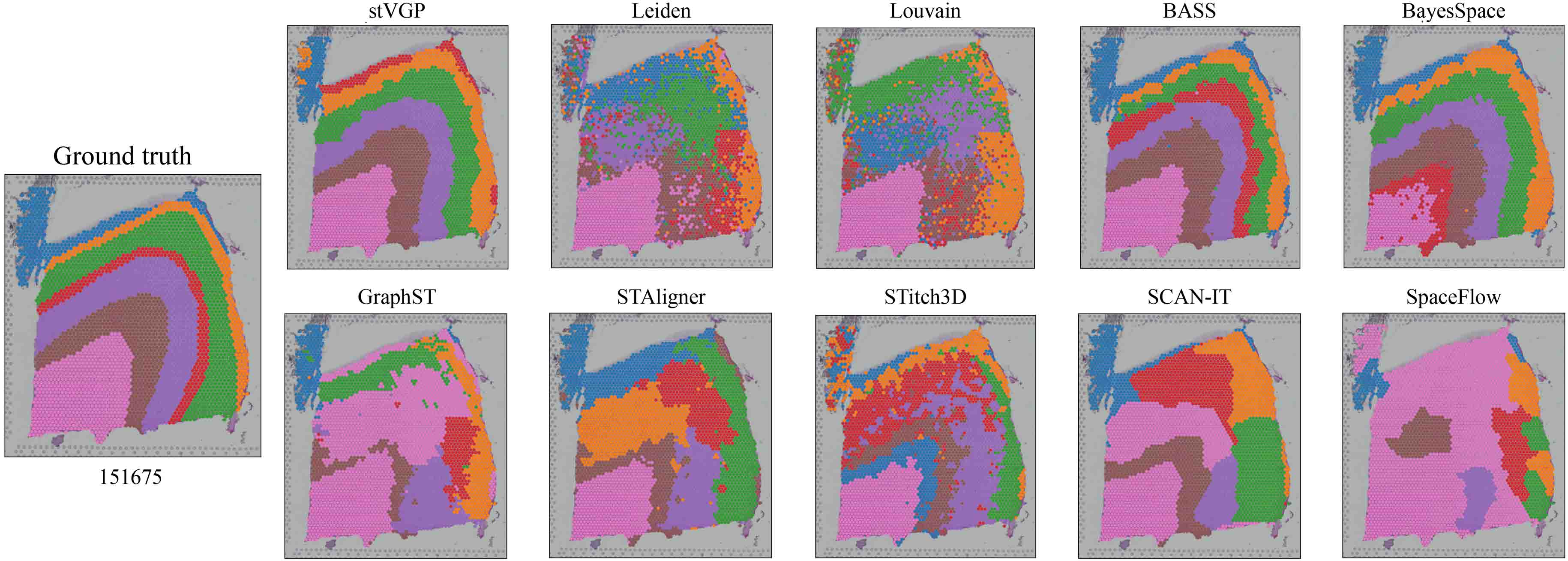


**Supplementary Figure 7**. **Comparison of spatial domain detection methods on DLPFC slice 151676.** Manual region annotations of slice 151676 are shown alongside spatial domain detection results generated by various computational methods. The evaluated methods include stVGP, Leiden, Louvain, BASS, BayesSpace, GraphST, STAligner, STitch3D, SCAN-IT, and SpaceFlow.


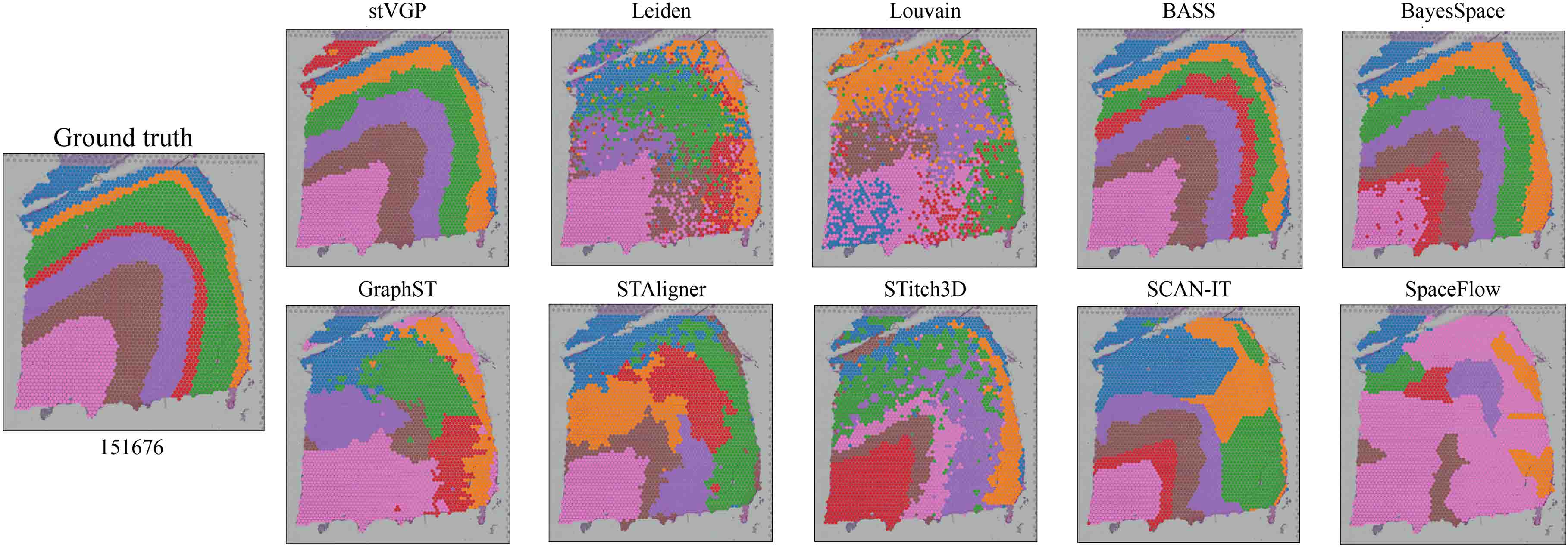


**Supplementary Figure 8**. **Quantitative comparison of spatial domain detection methods on DLPFC slice 151673.** Adjusted Rand Index (ARI) and Normalized Mutual Information (NMI) scores were computed for all methods on slice 151673 to evaluate clustering accuracy. In this comparison, stVGP achieved the highest performance across both metrics, indicating superior consistency with manual annotations.


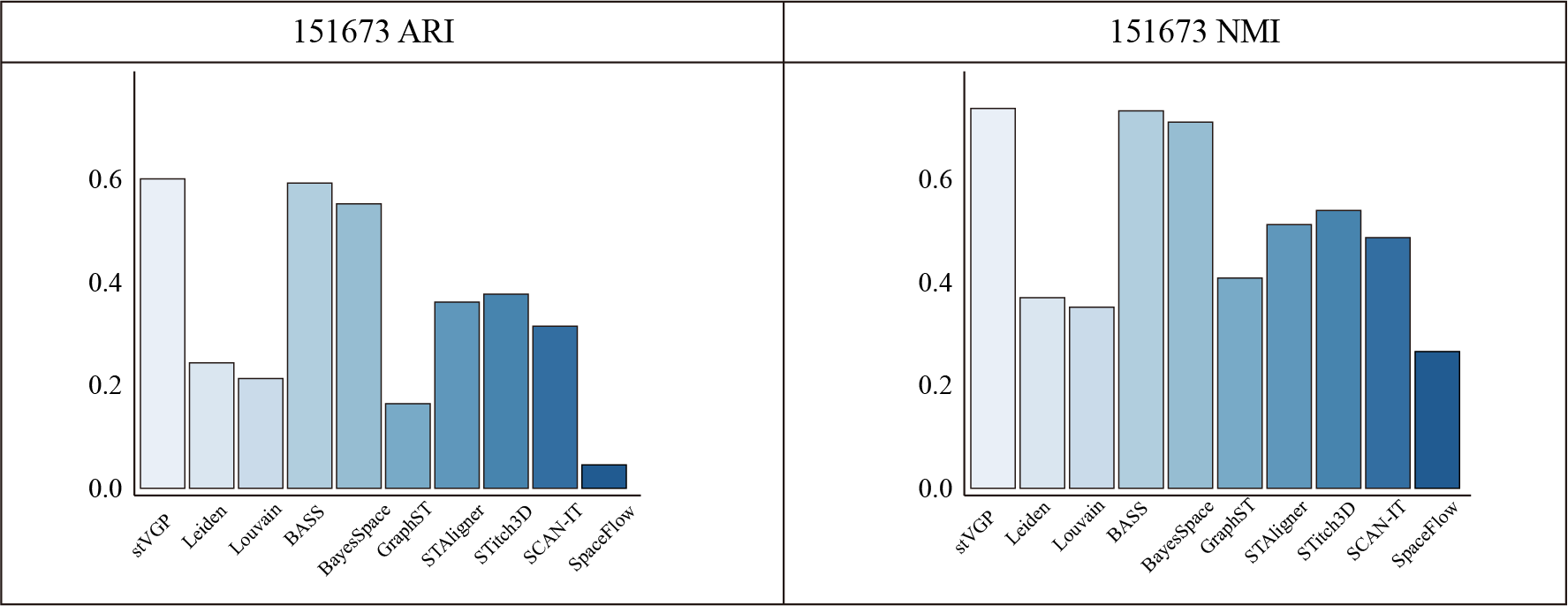


**Supplementary Figure 9**. **Quantitative comparison of spatial domain detection methods on DLPFC slice 151674.** Adjusted Rand Index (ARI) and Normalized Mutual Information (NMI) scores were computed for all methods on slice 151674 to evaluate clustering accuracy. In this comparison, stVGP achieved the highest performance across both metrics, indicating superior consistency with manual annotations.


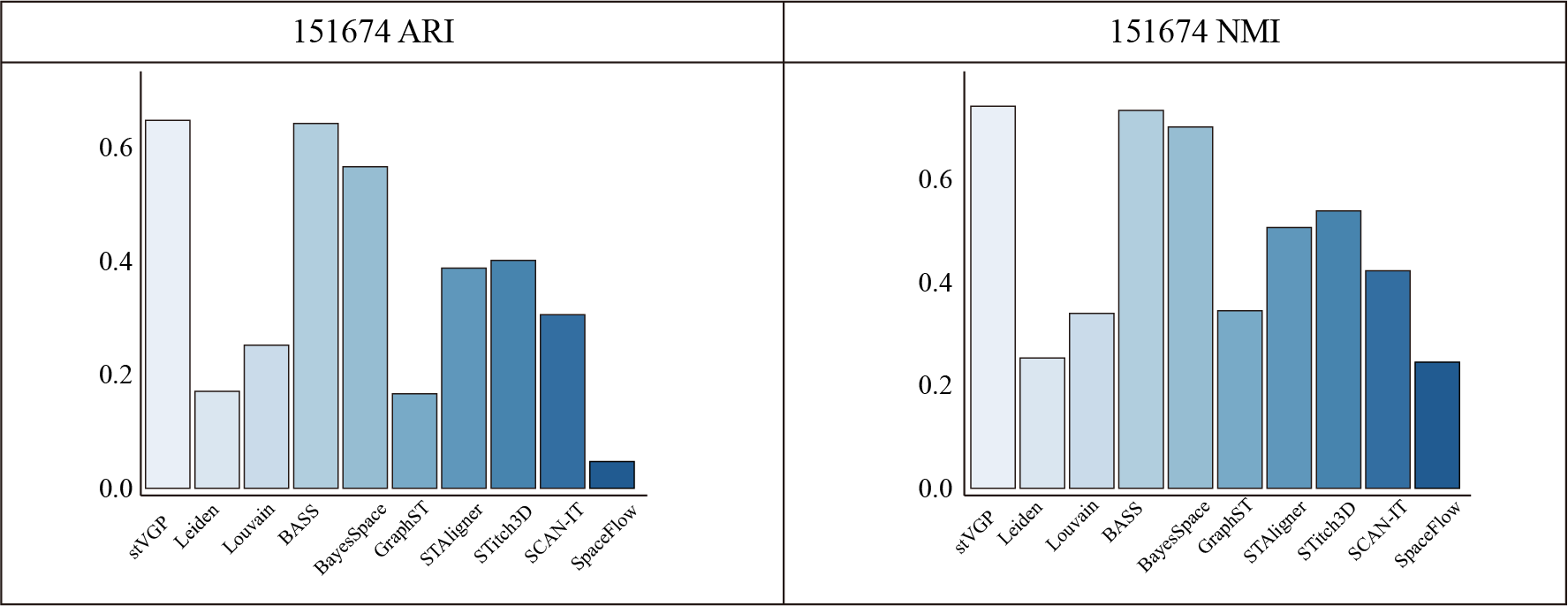


**Supplementary Figure 10**. **Quantitative comparison of spatial domain detection methods on DLPFC slice 151675.** Adjusted Rand Index (ARI) and Normalized Mutual Information (NMI) scores were computed for all methods on slice 151675 to evaluate clustering accuracy. In this comparison, stVGP achieved the second-highest performance across both metrics, surpassed only by the BASS method.


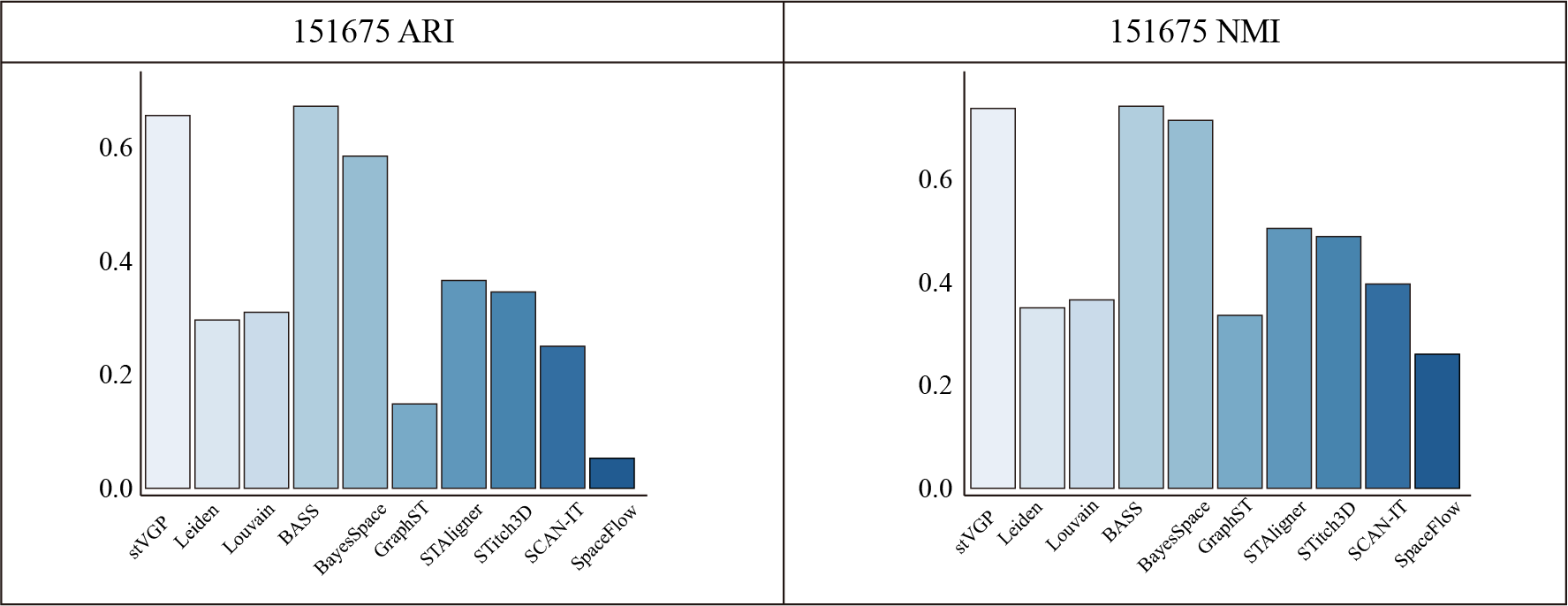


**Supplementary Figure 11**. **Quantitative comparison of spatial domain detection methods on DLPFC slice 151676.** Adjusted Rand Index (ARI) and Normalized Mutual Information (NMI) scores were computed for all methods on slice 151676 to evaluate clustering accuracy. In this comparison, stVGP achieved the second-highest performance across both metrics, surpassed only by the BASS method.


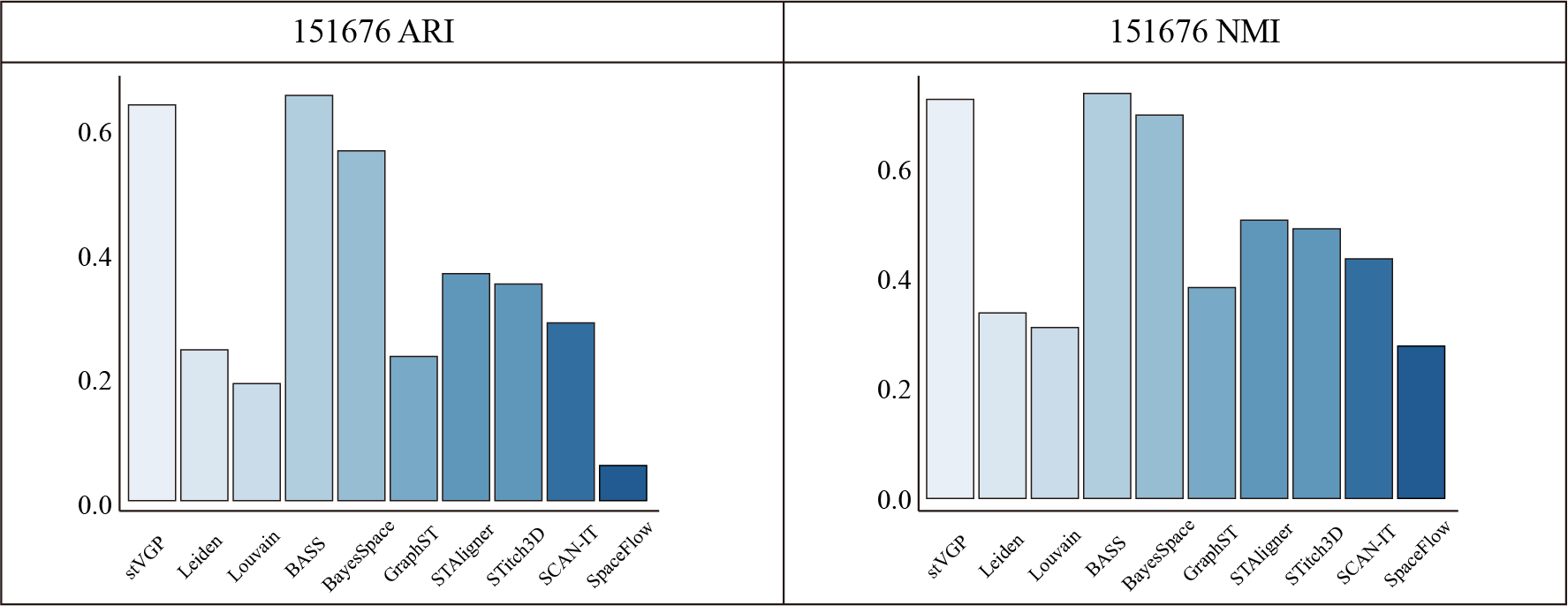


**Supplementary Figure 12**. **Benchmarking stVGP against SpaGCN for the task of spatial domain identification.** Comparison of spatial domain identification performance between SpaGCN and stVGP across four slices (151673–151676). The Adjusted Rand Index (ARI) score for each slice is displayed below the corresponding plot, indicating the superior consistency of stVGP in domain recognition.

**
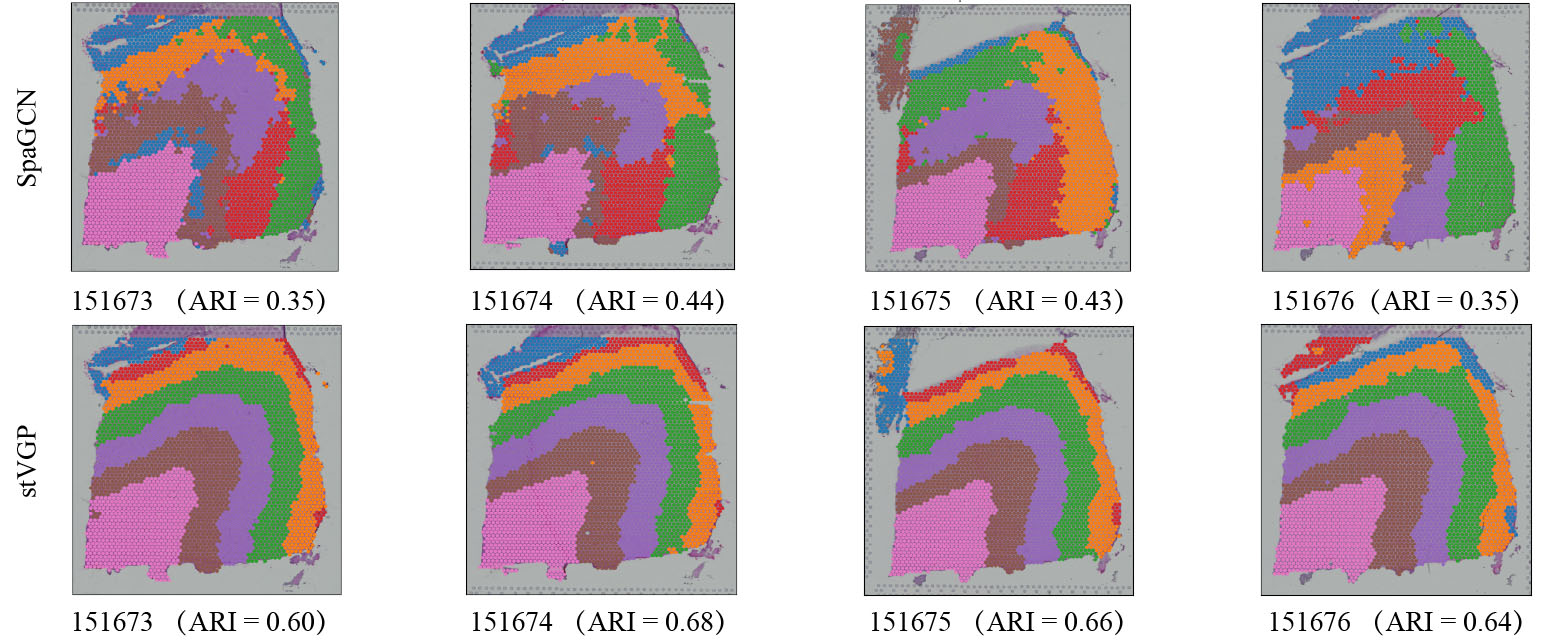
**

**Supplementary Figure 13**. **UMAP visualization of embeddings colored by manual annotations across integrated slices.** UMAP plots display the low-dimensional embeddings obtained by integrating all slices using ten spatial domain detection methods: stVGP, Leiden, Louvain, BASS, BayesSpace, GraphST, STAligner, STitch3D, SCAN-IT, and SpaceFlow. Among these, stVGP, BASS, BayesSpace, and STitch3D effectively preserved the hierarchical structure in the latent space. In contrast, SCAN-IT and SpaceFlow, which are not designed for multi-slice integration, showed suboptimal performance in capturing coherent low-dimensional representations.


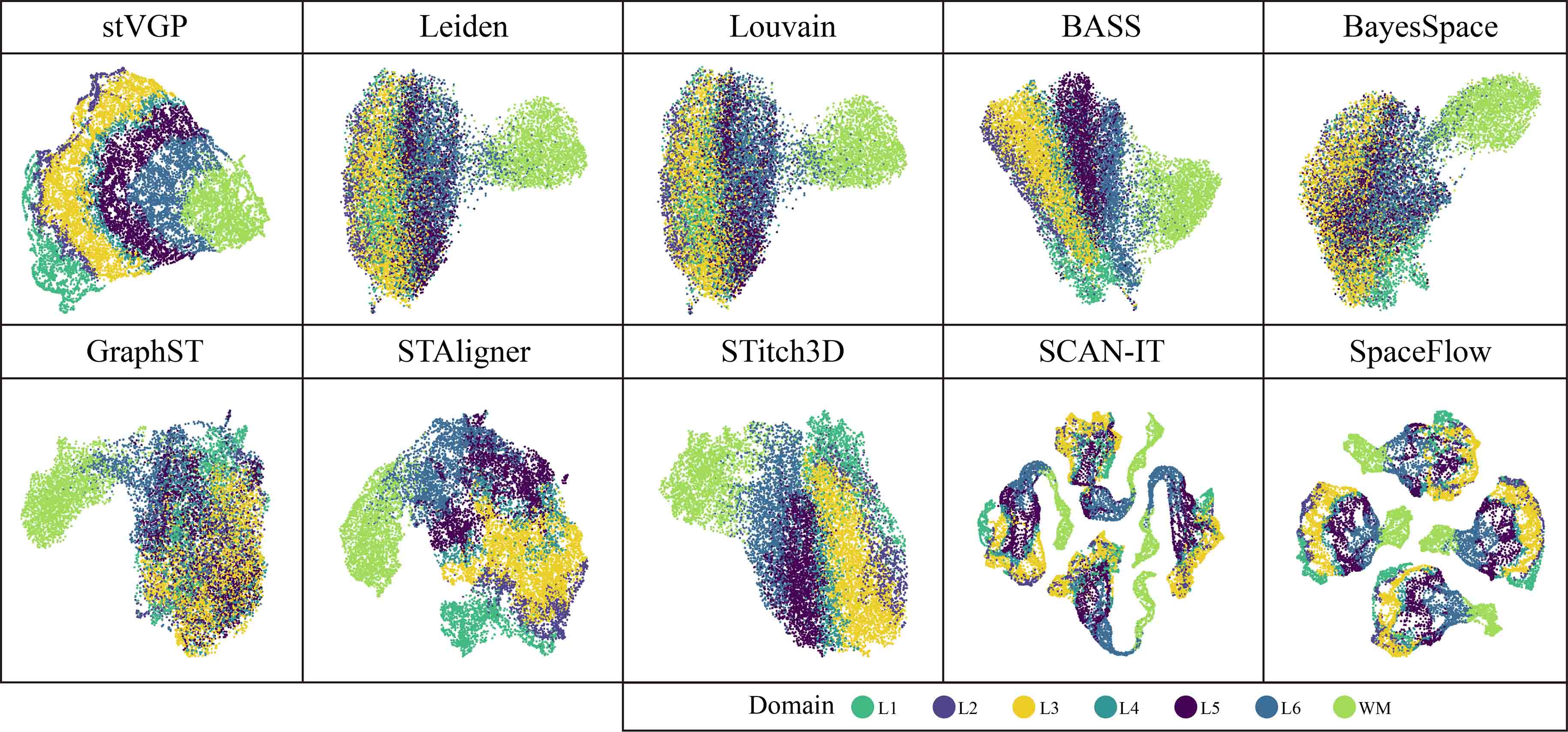


**Supplementary Figure 14**. **UMAP visualization of embeddings colored by clustering results across integrated slices.** UMAP plots show the low-dimensional embeddings derived from clustering results after integrating all slices using ten methods: stVGP, Leiden, Louvain, BASS, BayesSpace, GraphST, STAligner, STitch3D, SCAN-IT, and SpaceFlow. Among these, stVGP, BASS, and BayesSpace exhibited clustering patterns that reflect a hierarchical organization consistent with manual annotations. In contrast, the remaining methods failed to effectively capture and compress spatial information into meaningful low-dimensional representations.


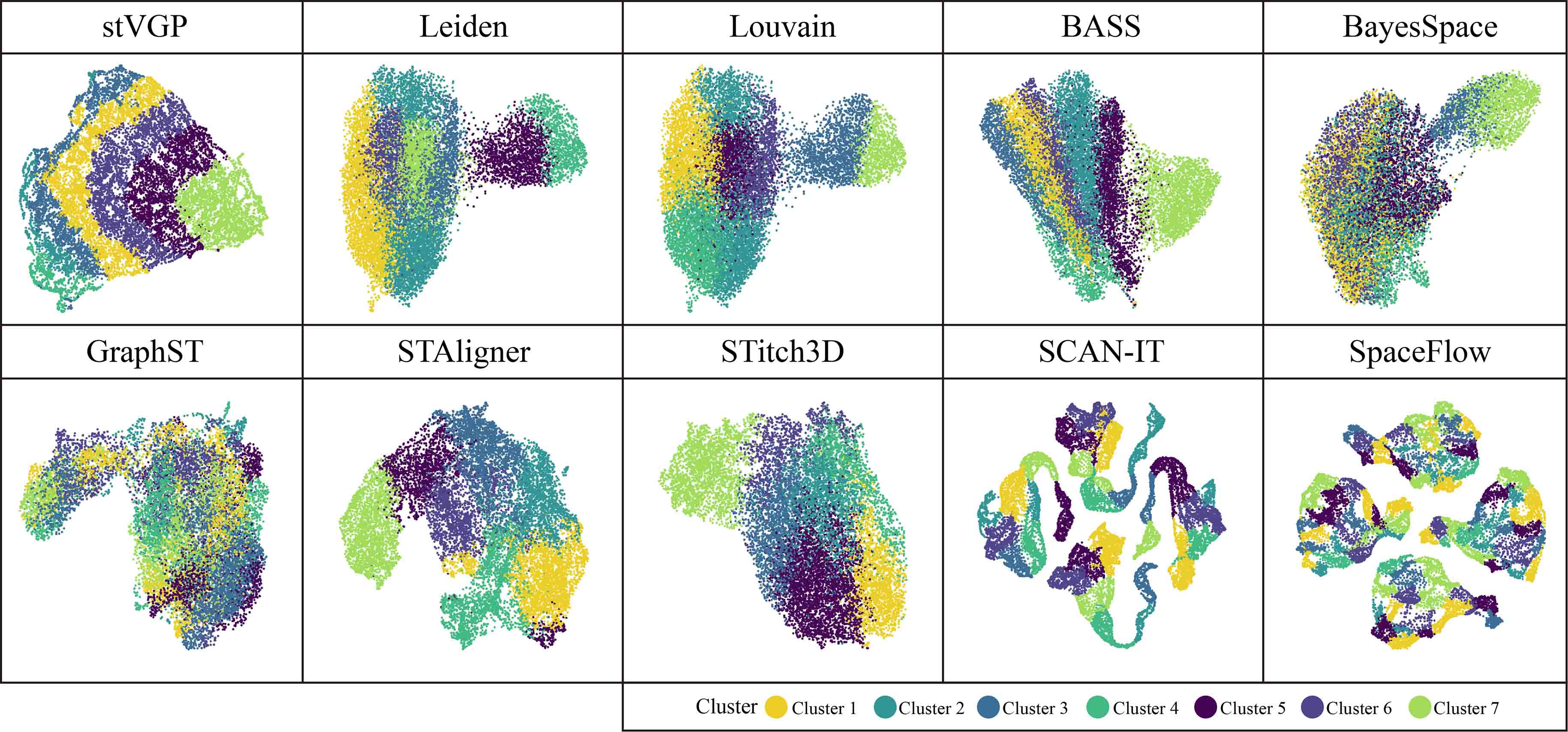


**Supplementary Figure 15**. **UMAP visualization of embeddings colored by slice labels across integrated slices.** In the DLPFC dataset analysis without batch correction, batch effects were observed between slices (e.g., Leiden or Louvain clustering result). Among all methods, stVGP successfully projected slice-specific information into low-dimensional embeddings. Although GraphST and STAligner also managed to map slice information into low-dimensional spaces, they overcorrected this information, leading to inaccuracies in the clustering results. SCAN-IT and SpaceFlow were not designed for multi-slice analysis, which explains the suboptimal performance observed with both methods.


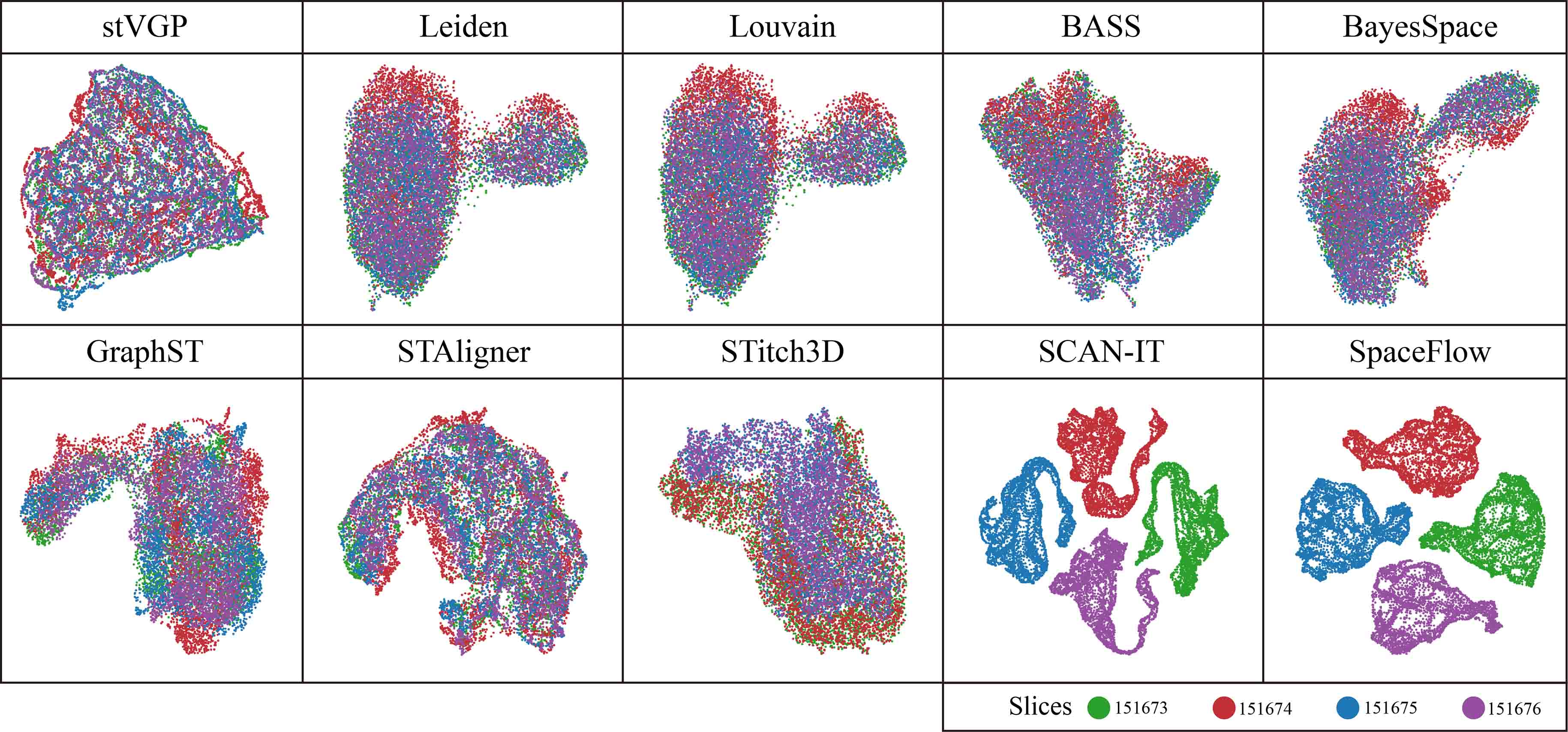


**Supplementary Figure 16**. **Benchmarking of spatial reconstruction methods. a, b.** Quantitative assessment of *MOBP* prediction accuracy evaluated by the Pearson correlation coefficient (a) and the Root Mean Square Error (RMSE) (b). **c, d.** Spatial visualization of *MOBP* expression reconstruction for slices 151674 (c) and 151675 (d). The panels display the ground truth expression alongside predictions from stVGP and five benchmarking methods, highlighting the visual fidelity of gene pattern recovery.

**
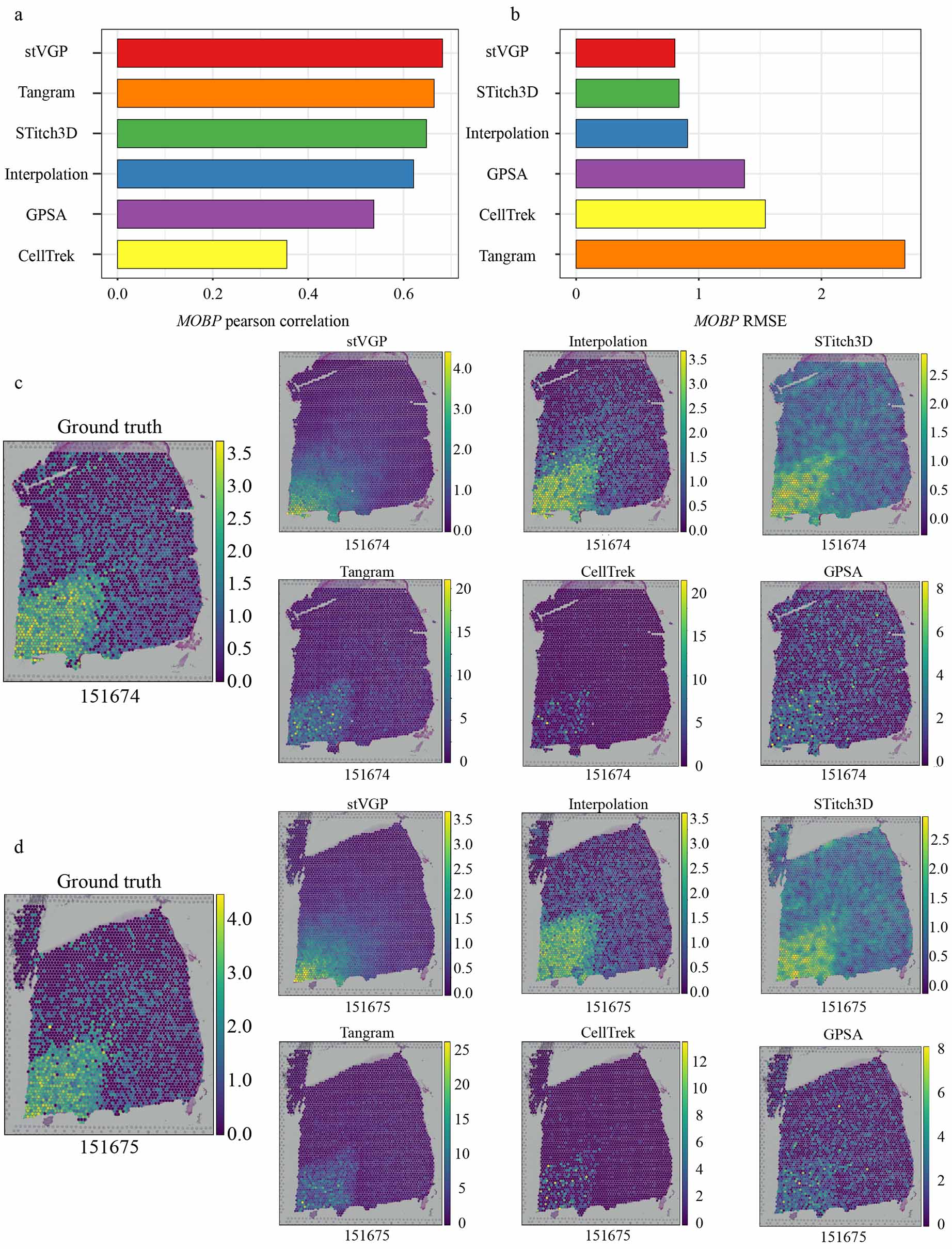
**

**Supplementary Figure 17**. **Differences between stVGP spatial reconstruction and single-cell spatial localization reconstruction. a.** Spatial visualization of *MOBP* expression predicted by stVGP, Tangram, and CellTrek compared to the ground truth across slices 151674 and 151675. **b, c, d.** Quantitative performance metrics for *MOBP* prediction, including Pearson correlation coefficient (b), Root Mean Square Error (RMSE) (c), and Structural Similarity Index (SSIM) (d). **e.** Comparison of computational running time (in seconds) among stVGP, Tangram, and CellTrek.

**
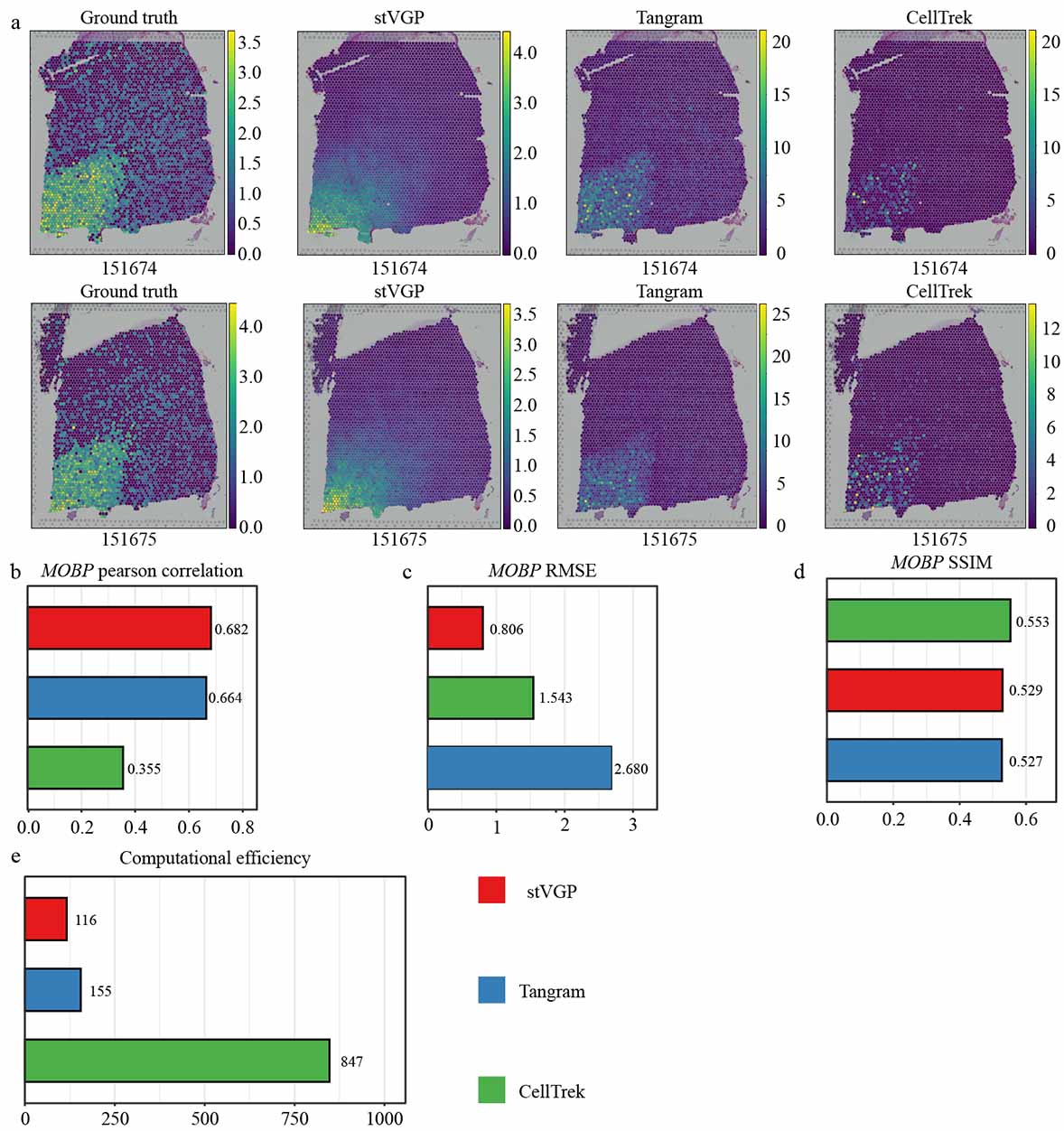
**

**Supplementary Figure 18**. **Benchmarking of computational efficiency of spatial reconstruction methods. a, b, c, d.** Bar plots comparing computational resource consumption across six methods (Interpolation, stVGP, Tangram, STitch3D, CellTrek, and GPSA). Evaluation metrics include total running time (a), total peak memory footprint (b), peak CPU memory usage (c), and peak GPU memory usage (d).


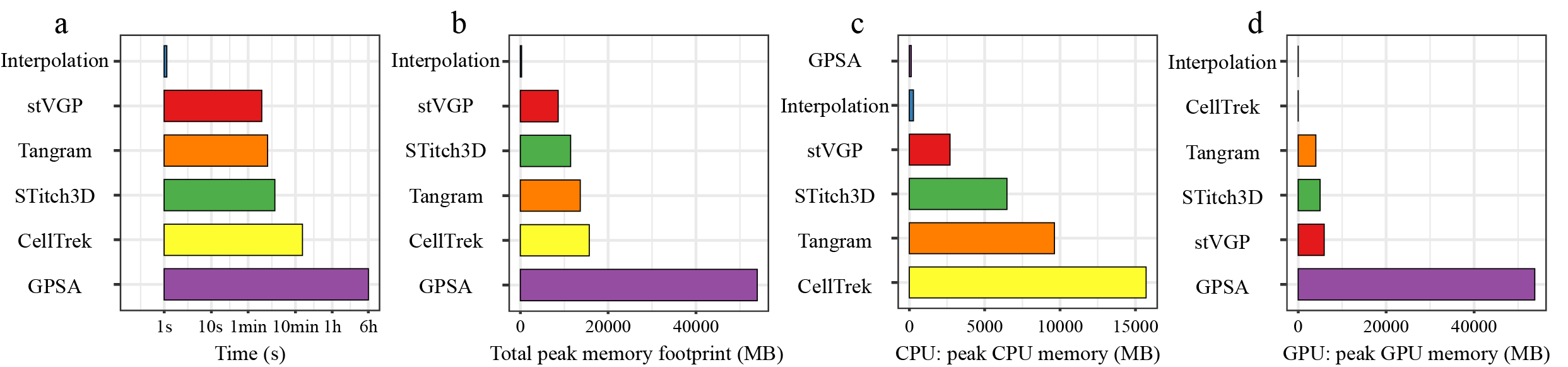


**Supplementary Figure 19**. **Comparison of spatial alignment methods on the adult mouse brain dataset^17^.** The adult mouse brain dataset contains 35 slices. We visualized the distribution of the all 35 slices before alignment. Subsequently, we visualized the effects of all alignment methods on the 35 slices. Here, stVGP, PASTE, PASTE2, and STitch3D demonstrated good alignment results and preliminarily reconstructed the three-dimensional structure of the tissue. In contrast, GPSA and STAligner demonstrated relatively lower performance.


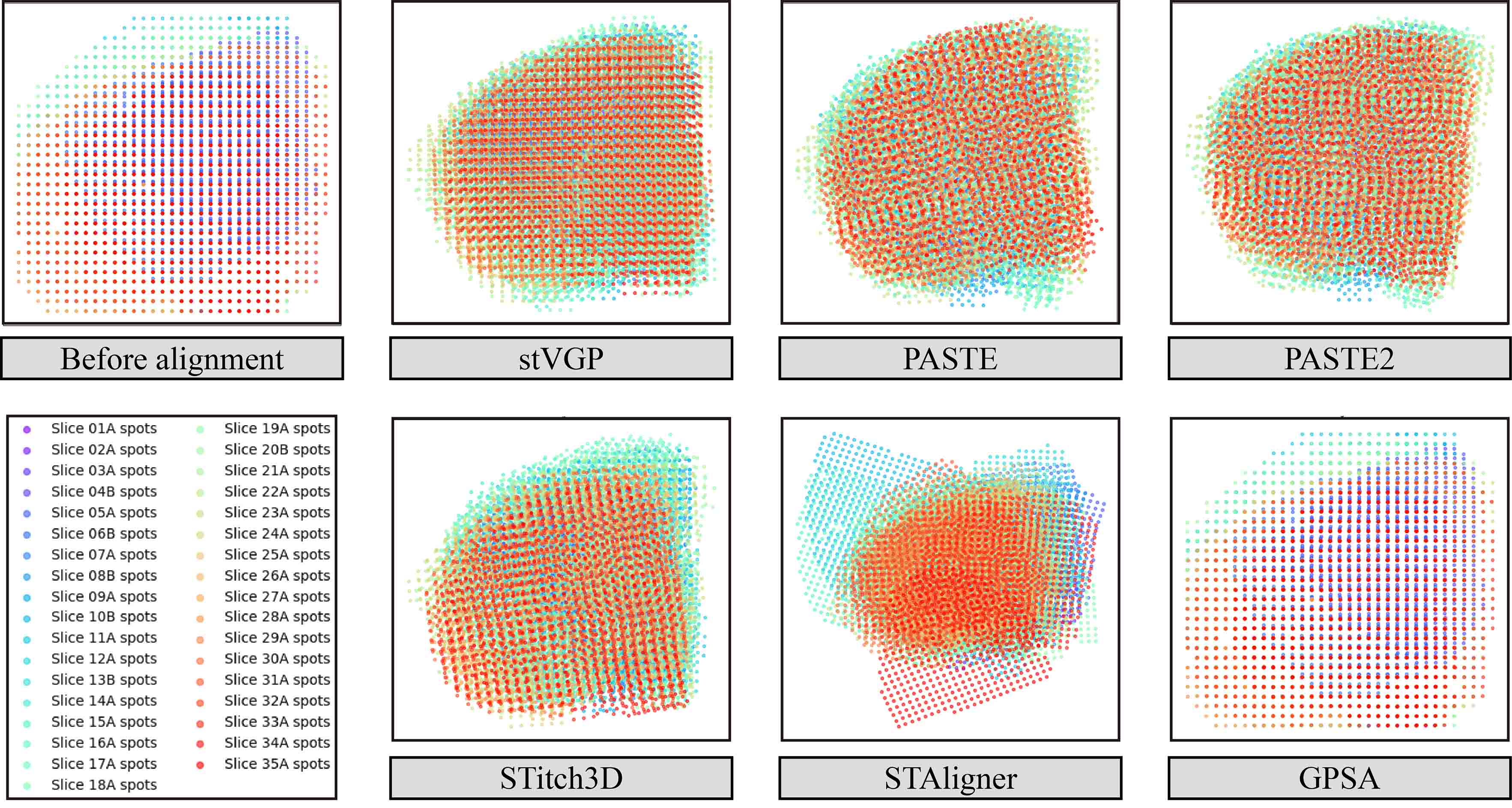


**Supplementary Figure 20**. **Quantitative comparison of tissue alignment methods on adult mouse brain dataset. a.** Mean alignment distance of all methods for 34 pairs of adjacent slices derived from the 35 slices. **b.** Median alignment distance of all methods for 34 pairs of adjacent slices derived from the 35 slices. **c.** box plots of alignment distances for 34 pairs of adjacent slices derived from the 35 slices. Here, we compute all the spot pairs in each pairs. Each box plot ranges from the third and first quartiles with the median as the horizontal line, while whiskers represent 1.5 times the interquartile range from the lower and upper bounds of the box. Among all the alignment methods, stVGP, PASTE, PASTE2, and STitch3D had better performances.


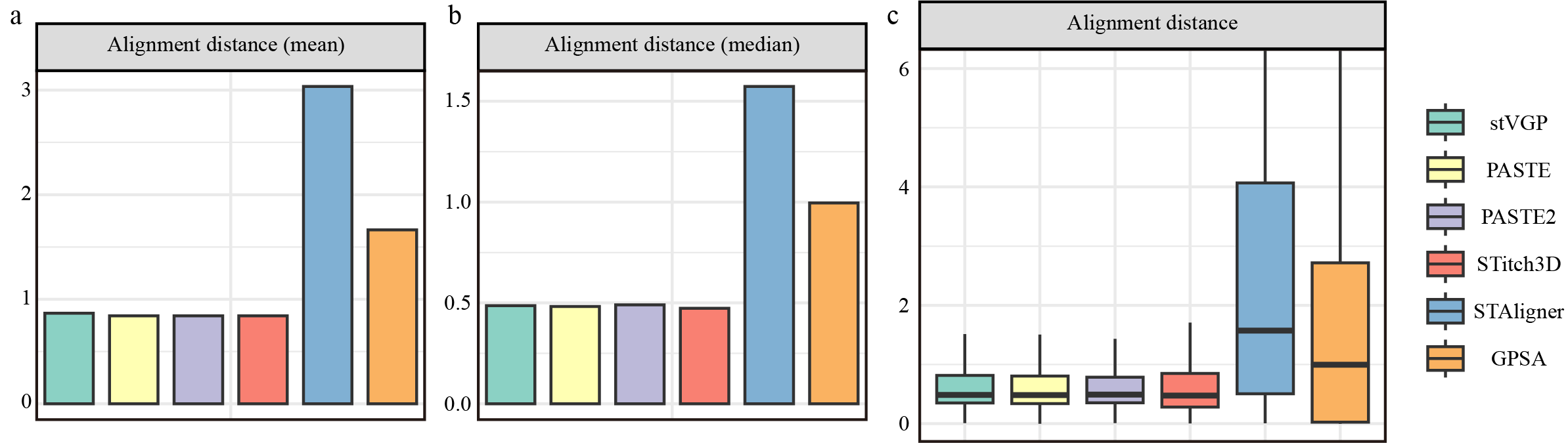


**Supplementary Figure 21**. **Comparison of results from spatial domain detection methods and manual annotation information on adult mouse brain dataset. a.** The annotation information for spatial domains 09A, 14A, and 19A, as provided in the original article. **b.** Clustering results of all methods on slices 09A, 14A, and 19A. In domain identification, the spatial domains detected by stVGP exhibited high consistency with the manually annotated tissue information.


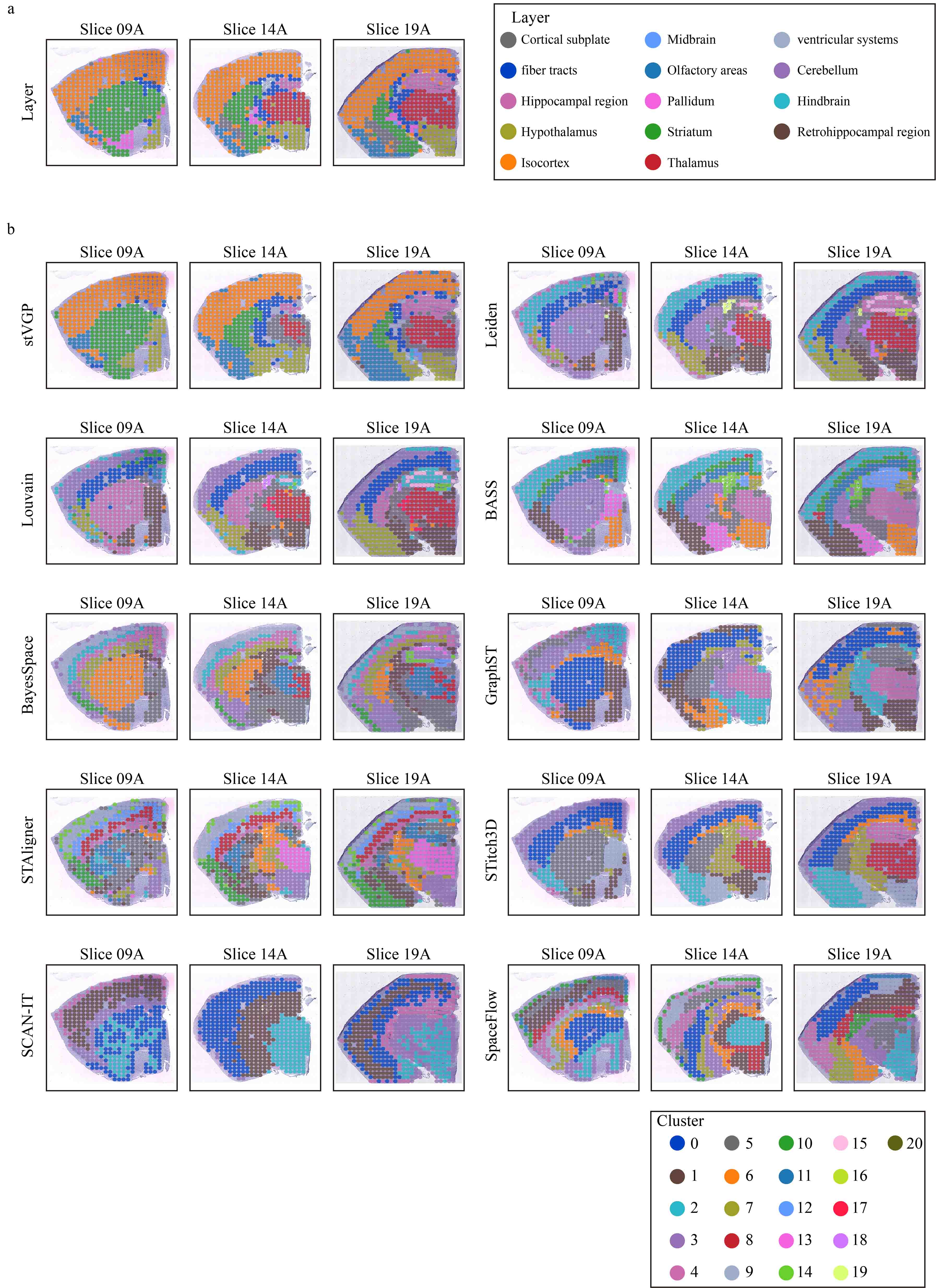


**Supplementary Figure 22**. **Comparison of results from spatial domain detection methods and manual annotation information on adult mouse brain dataset. a.** The annotation information for spatial domains 24A, 26A, and 31A, as provided in the original article. **b.** Clustering results of all methods on slices 24A, 26A, and 31A. In domain identification, the spatial domains detected by stVGP exhibited high consistency with the manually annotated tissue information.


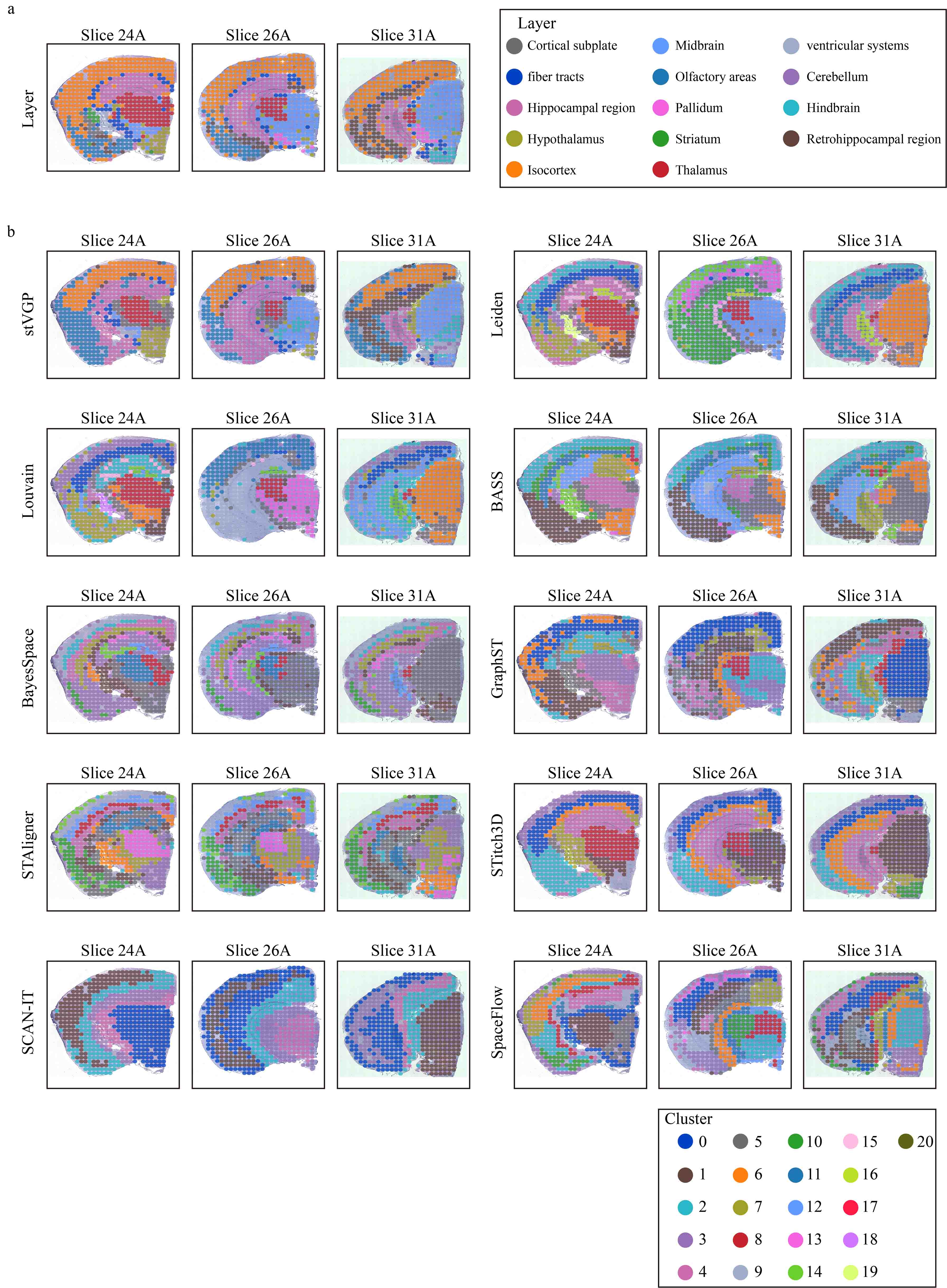


**Supplementary Figure 23**. **UMAP visualization of low-dimensional embeddings generated by all methods. a.** UMAP plots of embeddings colored by slice identity, obtained by integrating all slices using ten methods: stVGP, Leiden, Louvain, BASS, BayesSpace, GraphST, STAligner, STitch3D, SCAN-IT, and SpaceFlow. **b.** UMAP plots of embeddings colored by manual annotation labels, using the same set of methods. **c.** UMAP plots of embeddings colored by clustering results from each method after integrating all slices.


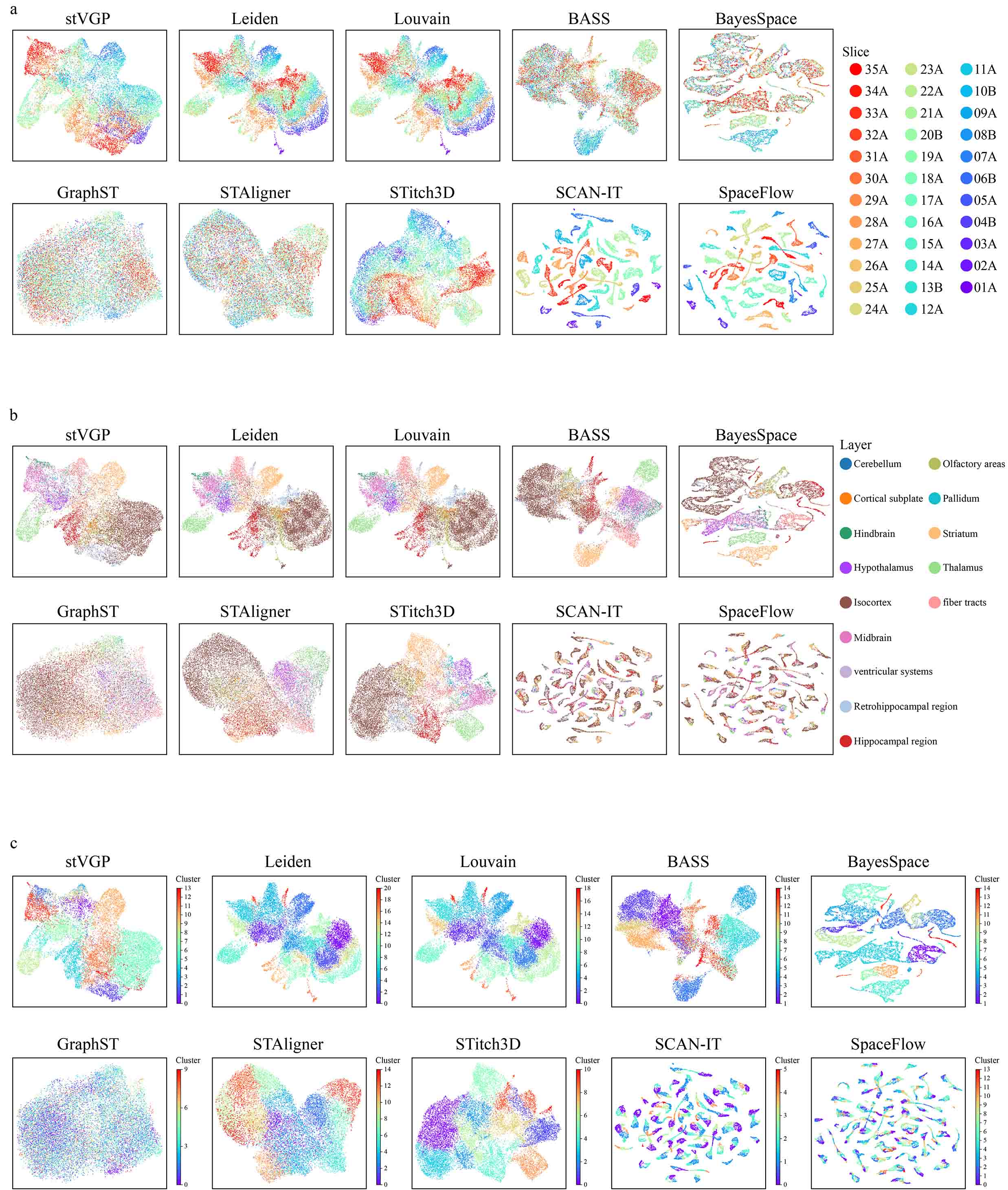


**Supplementary Figure 24**. **Spatial localization of cluster 7 (hypothalamus) and cluster 4 (hippocampal region) identified by stVGP in the adult mouse brain.** **a.** Visualization of cluster 7 on slices 10B, 12A, 14A, 16A, 18A, and 20B. **b.** 3D visualization and construction of cluster 7 (hypothalamus) in stVGP's aligned 3D coordinates. **c.** Visualization of cluster 4 on slices 20B, 22A, 24A, 26A, 28A, and 30A. **d.** 3D visualization and construction of cluster 4 (hippocampal region) in stVGP's aligned 3D coordinates. Here, stVGP effectively captured the localization and spatial distribution dynamics of both clusters within the adult mouse brain tissue. Notably, cluster 7 (hypothalamus) was consistently identified in the central region, while cluster 4 (hippocampal region) was primarily localized to the caudal region across the set of 35 aligned slices.


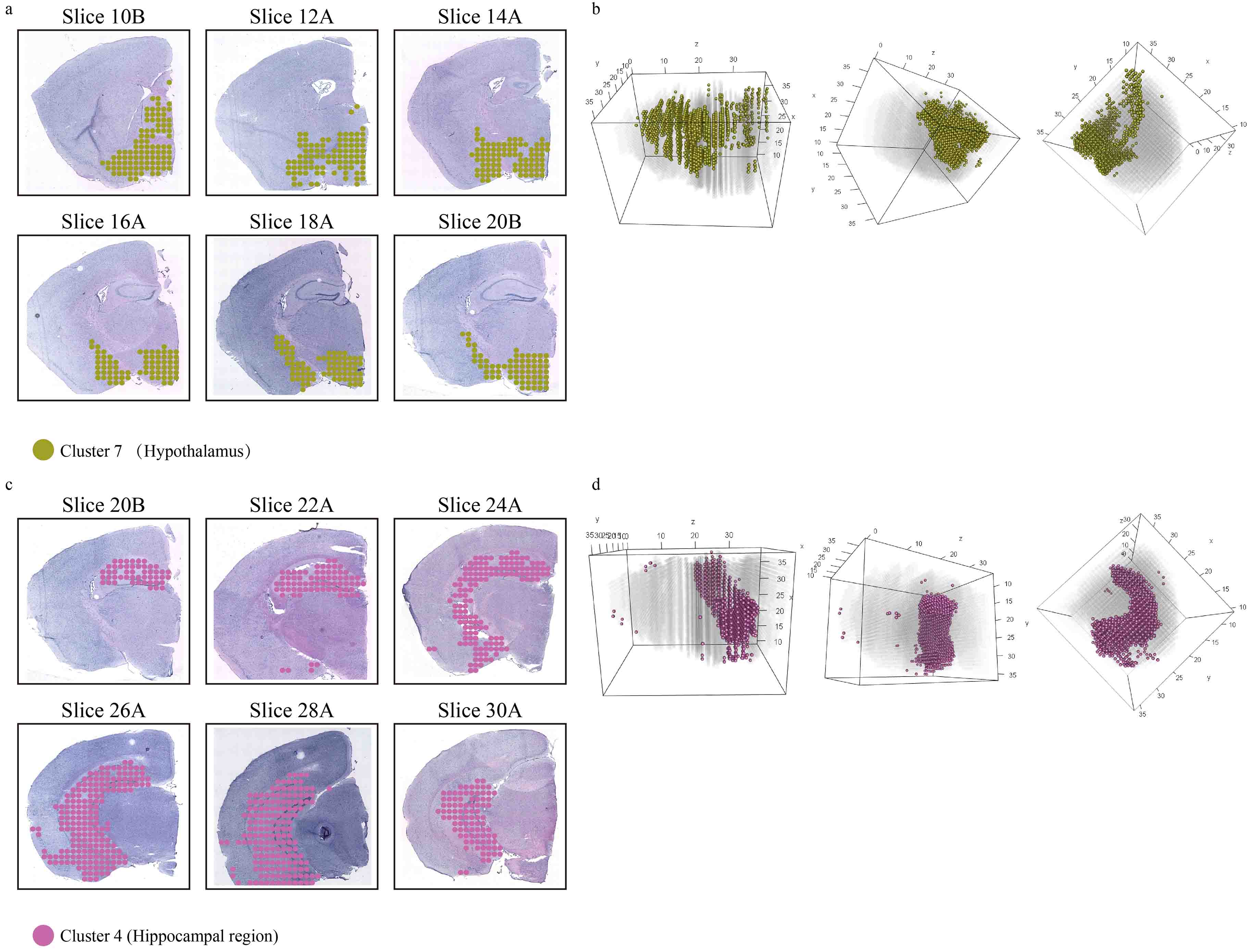


**Supplementary Figure 25**. **Comparison of spatial alignment methods on the developing human heart^18^.** Visualization of nine human heart slices before alignment and after alignment using six methods: stVGP, PASTE, PASTE2, STitch3D, STAligner, and GPSA. In results, stVGP, PASTE, PASTE2, and STitch3D demonstrate superior performance in aligning tissue structures. In contrast, GPSA and STAligner demonstrated relatively lower performance.


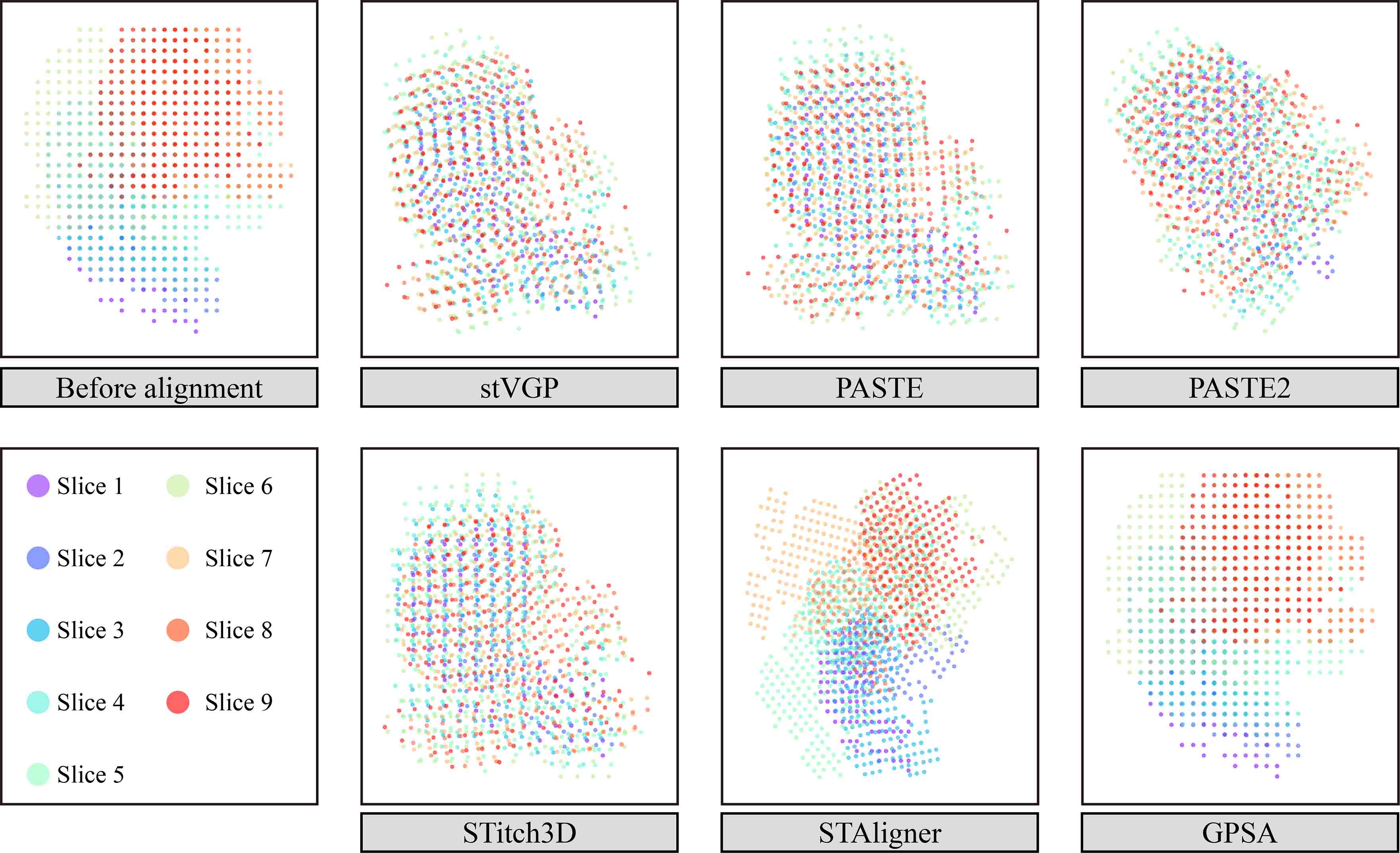


**Supplementary Figure 26**. **Quantitative comparison of tissue alignment methods on the developing human heart. a.** Mean alignment score of all methods across 8 adjacent slice pairs derived from the 9 slices. **b.** Median alignment distance of all methods across 8 adjacent slice pairs derived from the 9 slices. **c.** Box plots of alignment distances across 8 adjacent slice pairs derived from the 9 slices. Here, we compute all the spot pairs in each slice pair. Each box plot ranges from the third and first quartiles with the median as the horizontal line, while whiskers represent 1.5 times the interquartile range from the lower and upper bounds of the box. Among all the alignment methods, stVGP, PASTE, PASTE2, and STitch3D had better performances.


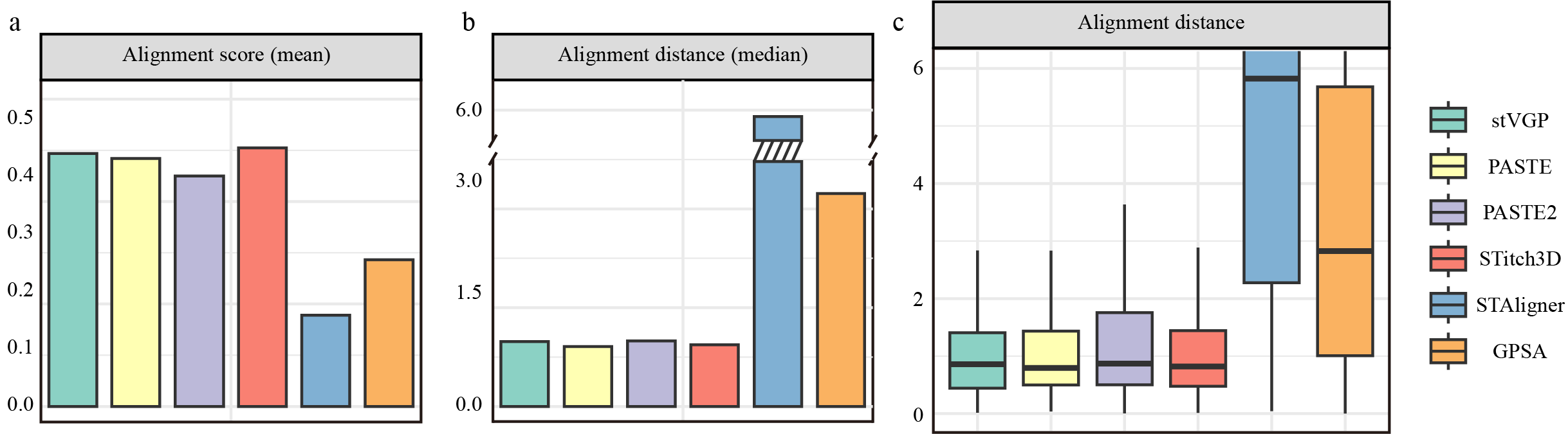


**Supplementary Figure 27**. **Comparison of spatial domain detection methods on the developing human heart dataset.** This figure presents the spatial distribution of domains identified by each method on the developing human heart dataset. Notably, manual annotations are not available for this dataset; only the analysis results from the original publication are provided. Therefore, we did not compare the results of the domain detection methods with the results reported in the original study.


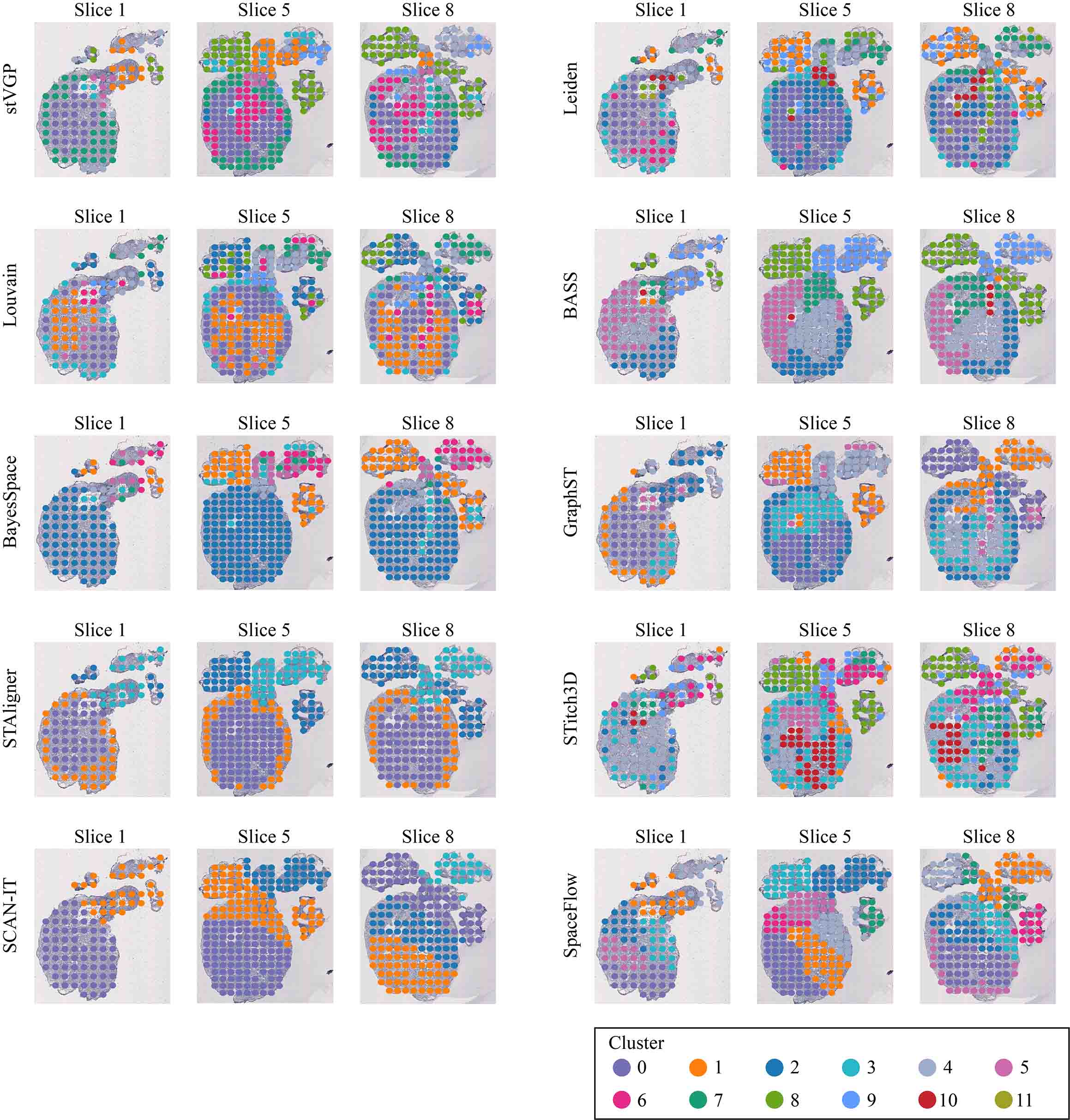


**Supplementary Figure 28**. **UMAP visualization of clustering and slice information across spatial domain detection methods.** **a.** UMAP plots of low-dimensional embeddings colored by clustering results, obtained by integrating all slices using ten methods: stVGP, Leiden, Louvain, BASS, BayesSpace, GraphST, STAligner, STitch3D, SCAN-IT, and SpaceFlow. **b.** UMAP plots of low-dimensional embeddings colored by slice identity, using the same set of methods. Notably, stVGP effectively mapped slice-specific information into the low-dimensional space while mitigating potential batch effects, resulting in improved inter-slice integration.


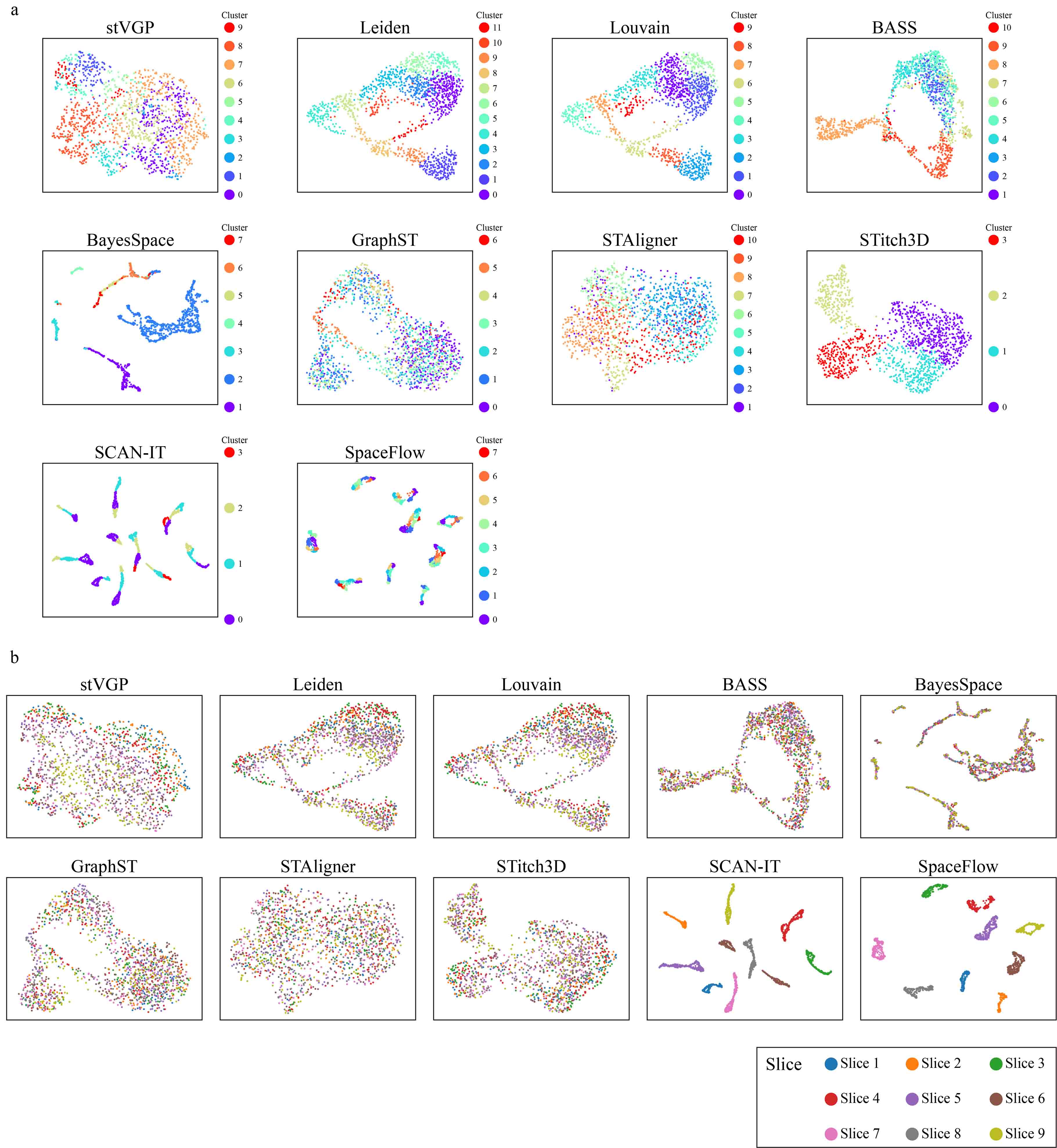


**Supplementary Figure 29**. **Benchmarking batch effect integration and biological conservation across multiple methods.** UMAP visualizations of the raw data and data integrated by six different methods (stVGP, STitch3D, STAligner, MENDER, Harmony, and DeepST). Plots are colored by developmental stage batches (4.5-5 PCW, 6.5 PCW, 9 PCW) and individual slices to visualize batch mixing effects. The batch Average Silhouette Width (bASW) score is provided below each integrated plot as a quantitative metric for batch mixing.

**
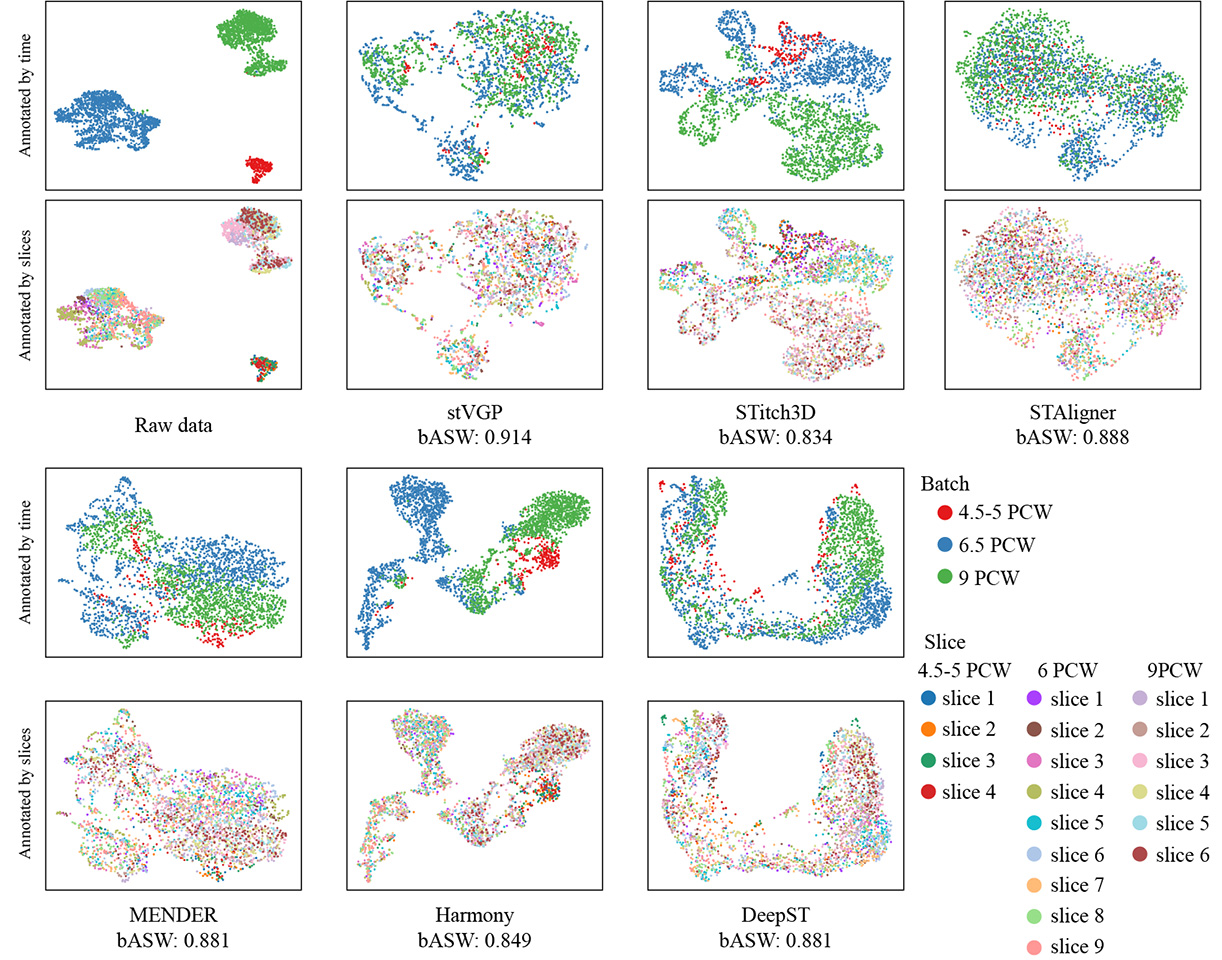
**

**Supplementary Figure 30**. **Spatial trajectory inference and molecular dynamics in the developing human heart. a.** UMAP visualization of the inferred developmental trajectory, colored by spatial domains. The arrow indicates the direction of differentiation from trabecular ventricular myocardium to oxidative-metabolism-enriched phenotypes. **b.** Projection of inferred pseudotime values onto the spatial coordinates of slices from three distinct developmental stages (4.5-5 PCW, 6.5 PCW, and 9 PCW), showing the correspondence between pseudotime progression and physical time. **c.** Heatmap displaying the expression dynamics of spatially variable genes ordered by pseudotime. **d.** Quantitative expression trends of four representative genes (*CPE*, *SLIT3*, *MYL2*, *MB*) along the pseudotime axis. The shaded area represents the confidence interval. e, f. Spatial expression visualization of *SLIT3* (e) and *MYL2* (f) across the three developmental stages. These plots validate the alignment between the model-inferred trajectory and the actual spatial expression patterns of marker genes.

**
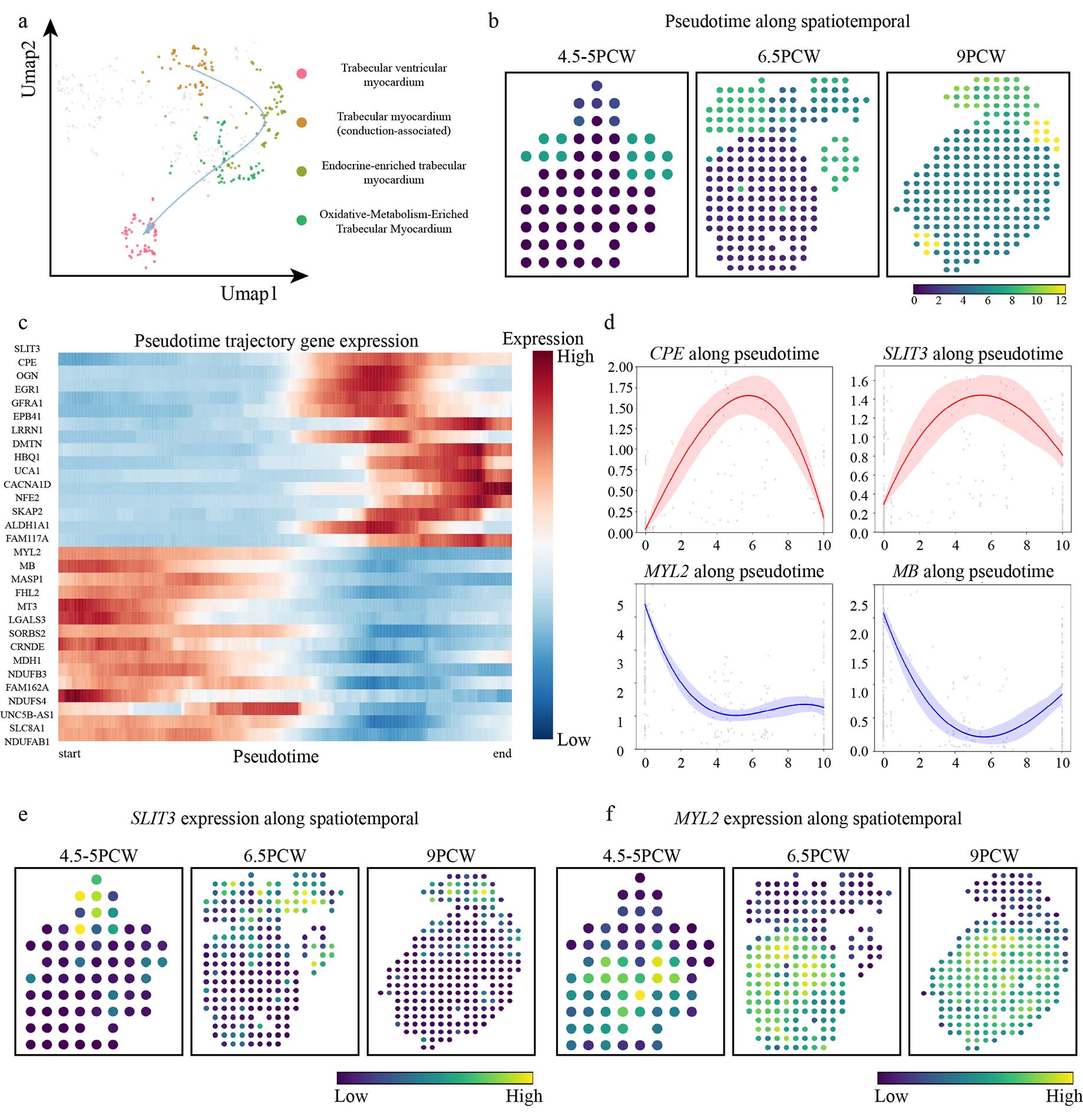
**

**Supplementary Figure 31**. **Characterization of spatial domains and developmental trajectories in the developing human heart. a.** Heatmaps displaying the expression of the top 4 marker genes for spatial domains at 6.5 PCW (left) and 9 PCW (right), indicating conserved domain identities across developmental stages. **b.** Volcano plots highlighting differentially expressed genes (DEGs) for Domain 0 (Trabecular ventricular myocardium), Domain 1 (Outflow tract / large vessels), and Domain 8 (Atrial myocardium). **c.** Gene Ontology (GO) enrichment analysis of marker genes in Domain 0. **d.** UMAP visualization of the inferred developmental trajectory, colored by spatial domains. **e.** Projection of inferred pseudotime values onto the spatial coordinates. **f.** Heatmap of gene expression dynamics along the inferred pseudotime trajectory. **g, h.** Quantitative expression trends (g) and spatial feature plots (h) of representative genes *FOSB* and *MYL2*, validating the correspondence between pseudotime dynamics and spatial localization.

**
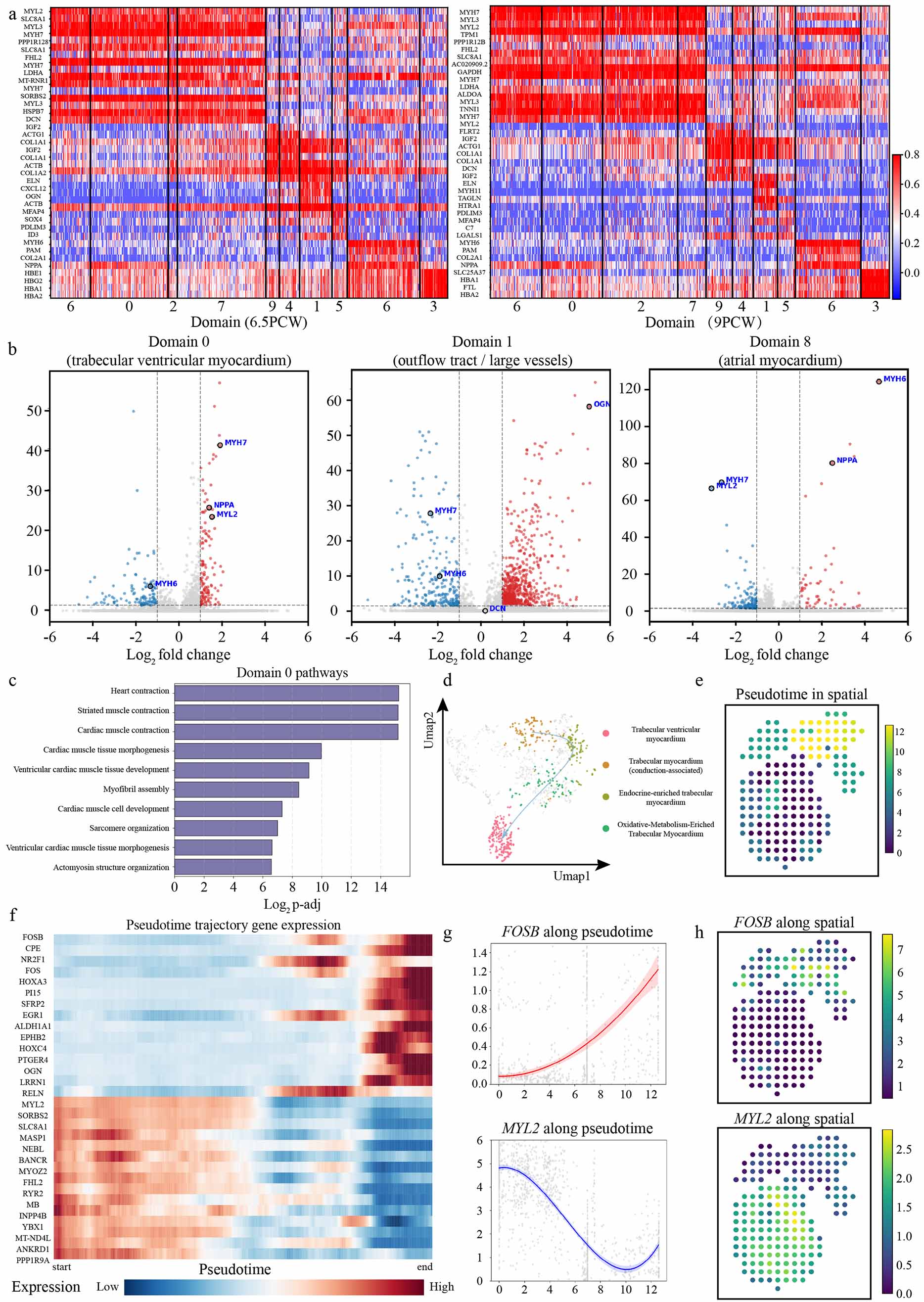
**

**Supplementary Figure 32**. **Spatial localization of** **Cluster 6 (trabecular ventricular myocardium - 2) and cluster 7 (trabecular ventricular myocardium - 3) identified by stVGP in the developing human heart dataset. a.** Visualization of the detected domains in stVGP-aligned 3D coordinates of 6.5-post-conception-week (PCW) slices, alongside a reference heart model from a Carnegie Stage 18 (CS18-6524) embryo^29^. **b.** Distribution and variation of spatial domains across slices, as identified by stVGP. **c.** 3D visualization and construction of cluster 6 (trabecular ventricular myocardium - 2) in stVGP's aligned 3D coordinates. **d.** 3D visualization and construction of cluster 7 (trabecular ventricular myocardium - 3) in stVGP's aligned 3D coordinates. These results demonstrate that stVGP effectively captured the localization patterns of trabecular ventricular myocardium subtypes, offering a more refined interpretation of spatial microenvironmental changes within developing cardiac tissue.


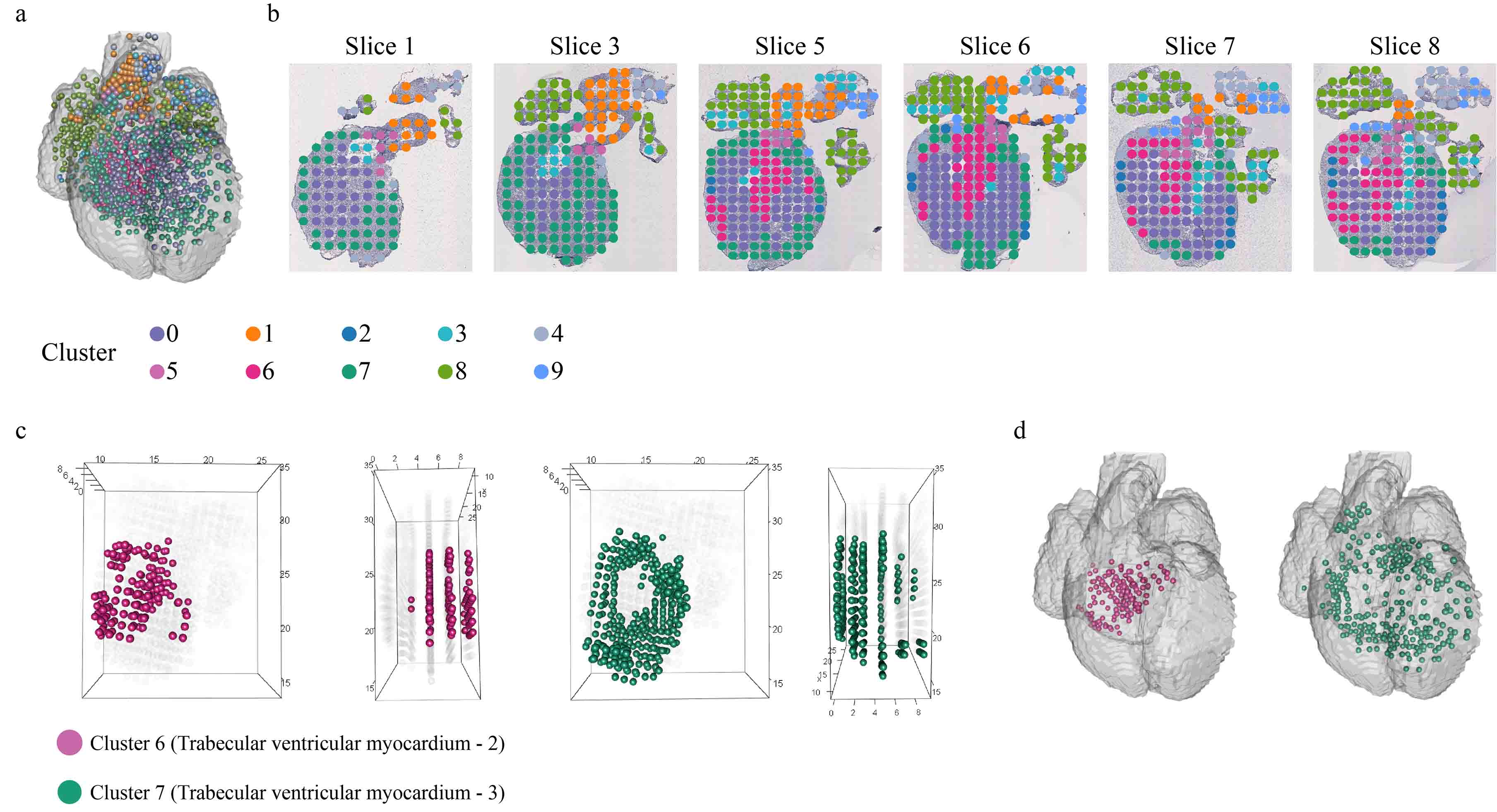


**Supplementary Figure 33**. **Comparison of spatial alignment methods on three individuals mouse olfactory bulb (MOB) dataset^30^.** Visualization of three MOB slices before alignment and visualization of alignment using stVGP, PASTE, PASTE2, STitch3D, STAligner, and GPSA. Here, the stVGP, PASTE, and PASTE2 methods demonstrated superior performance.


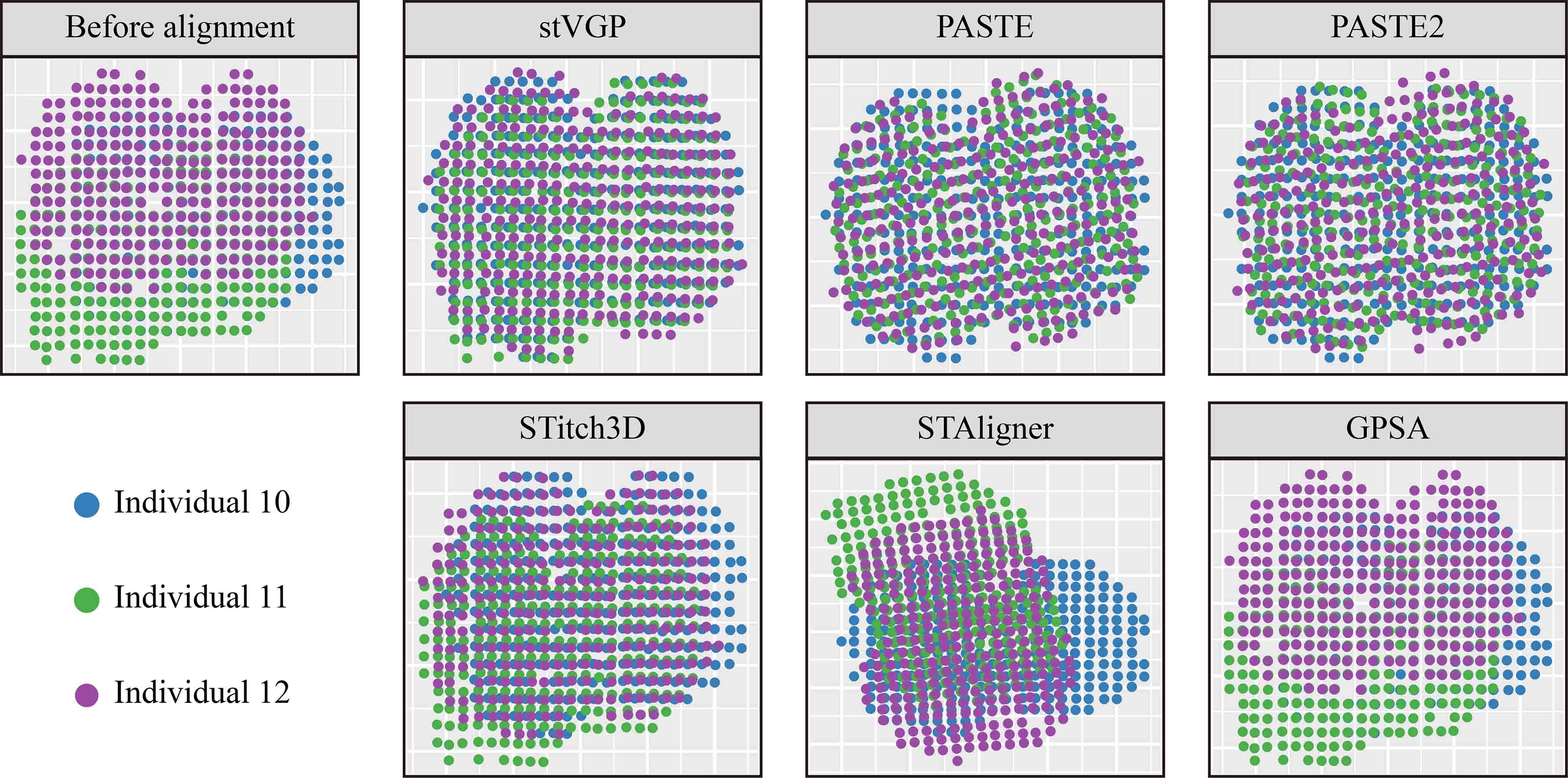


**Supplementary Figure 34**. **Quantitative comparison of alignment methods on mouse olfactory bulb (MOB) dataset.** **a.** Alignment score of all methods for pair 1 on MOB dataset. All methods, including stVGP, jointly analyzed three MOB slices. **b.** Alignment score of all methods for pair 2 on MOB dataset. **c.** Mean alignment distances of all methods computed across both adjacent slice pairs. **d.** Median alignment distances of all methods for the same slice pairs. **e.** Box plots of alignment distances for all pairs of adjacent slices derived from the three slices. Here, we compute all the spot pairs in each pairs. Each box plot ranges from the third and first quartiles with the median as the horizontal line, while whiskers represent 1.5 times the interquartile range from the lower and upper bounds of the box.


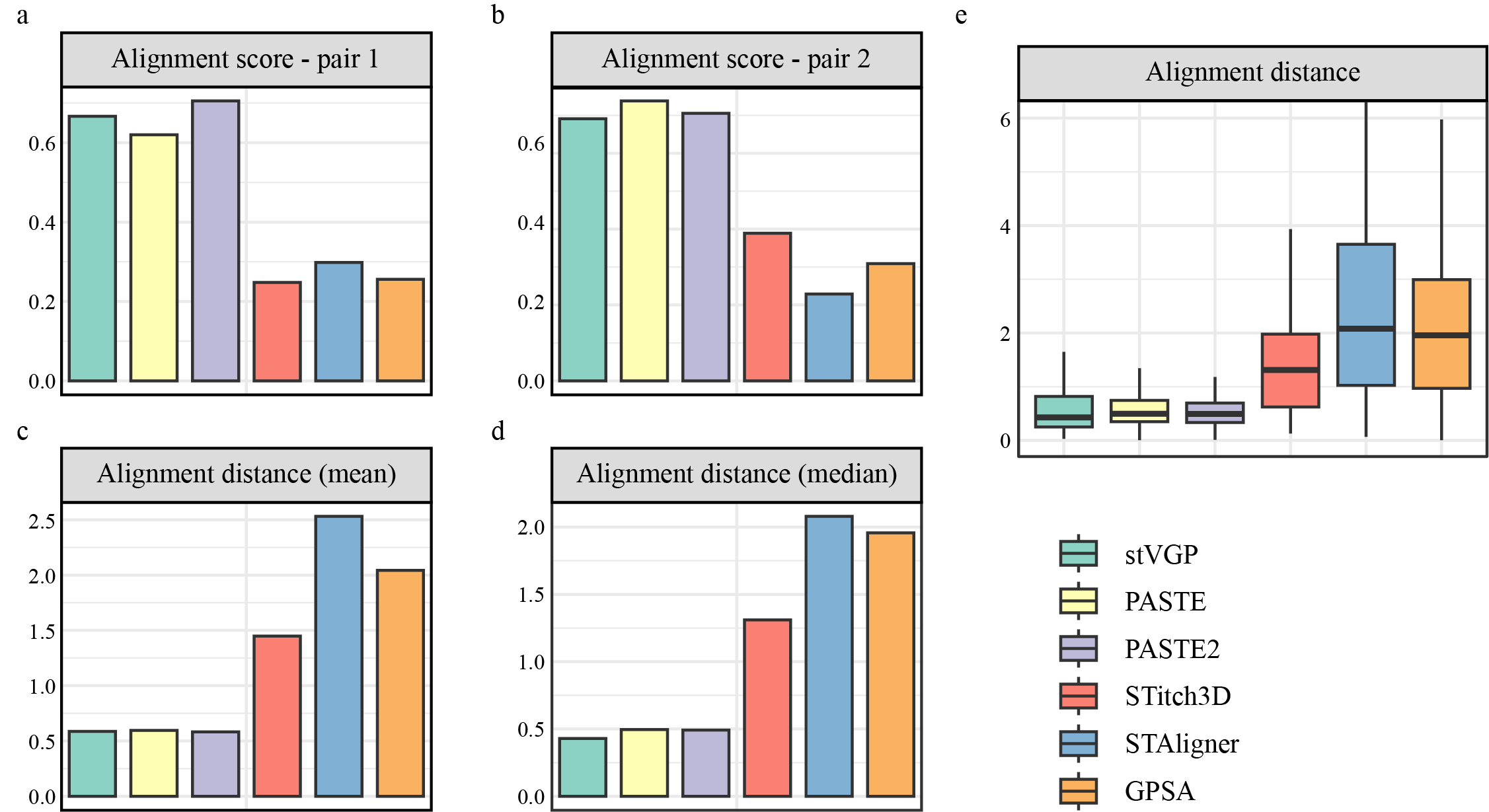


**Supplementary Figure 35**. **UMAP visualization of learned representation colored by clustering and slice identity across all methods on MOB dataset. a.** UMAP plots of low-dimensional embeddings, with colors representing clustering results obtained by integrating all slices using stVGP, Leiden, Louvain, BASS, BayesSpace, GraphST, STAligner, STitch3D, SCAN-IT, and SpaceFlow. **b.** UMAP plots of low-dimensional embeddings colored by slice identity, using the same set of methods.


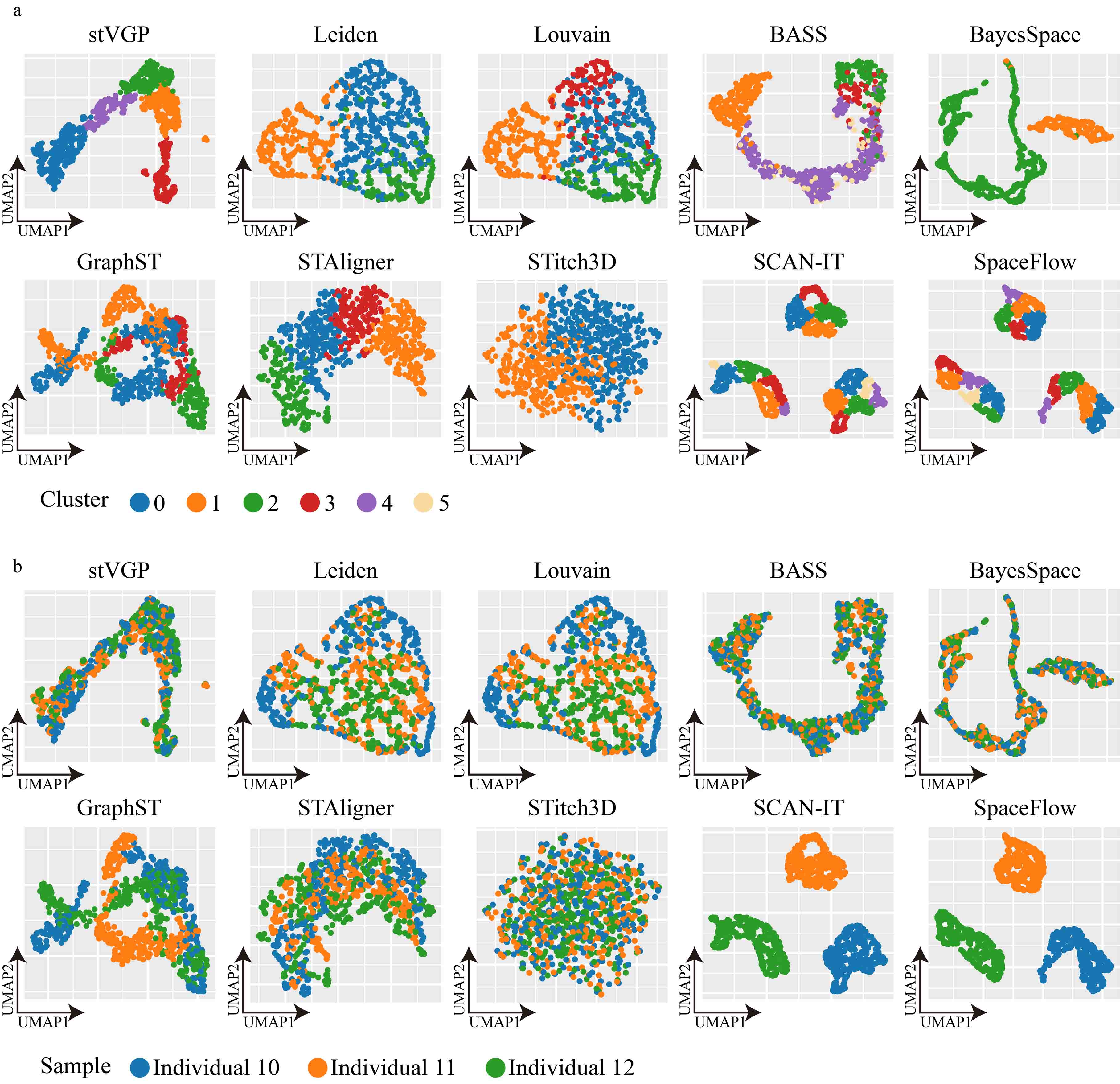


**Supplementary Figure 36**. **Quantitative comparison of alignment methods on human breast cancer dataset^30^. a, b, c.** Alignment score of all methods for pairs 1-3 on human breast cancer dataset. All methods, including stVGP, collectively analyzed four adjacent tissue slices. **d, e.** Mean and median alignment distance for all methods, computed across the three adjacent slice pairs. **f.** Box plots of alignment distances for all pairs of adjacent slices derived from the four slices. Here, we compute all the spot pairs in each pairs. Each box plot ranges from the third and first quartiles with the median as the horizontal line, while whiskers represent 1.5 times the interquartile range from the lower and upper bounds of the box.


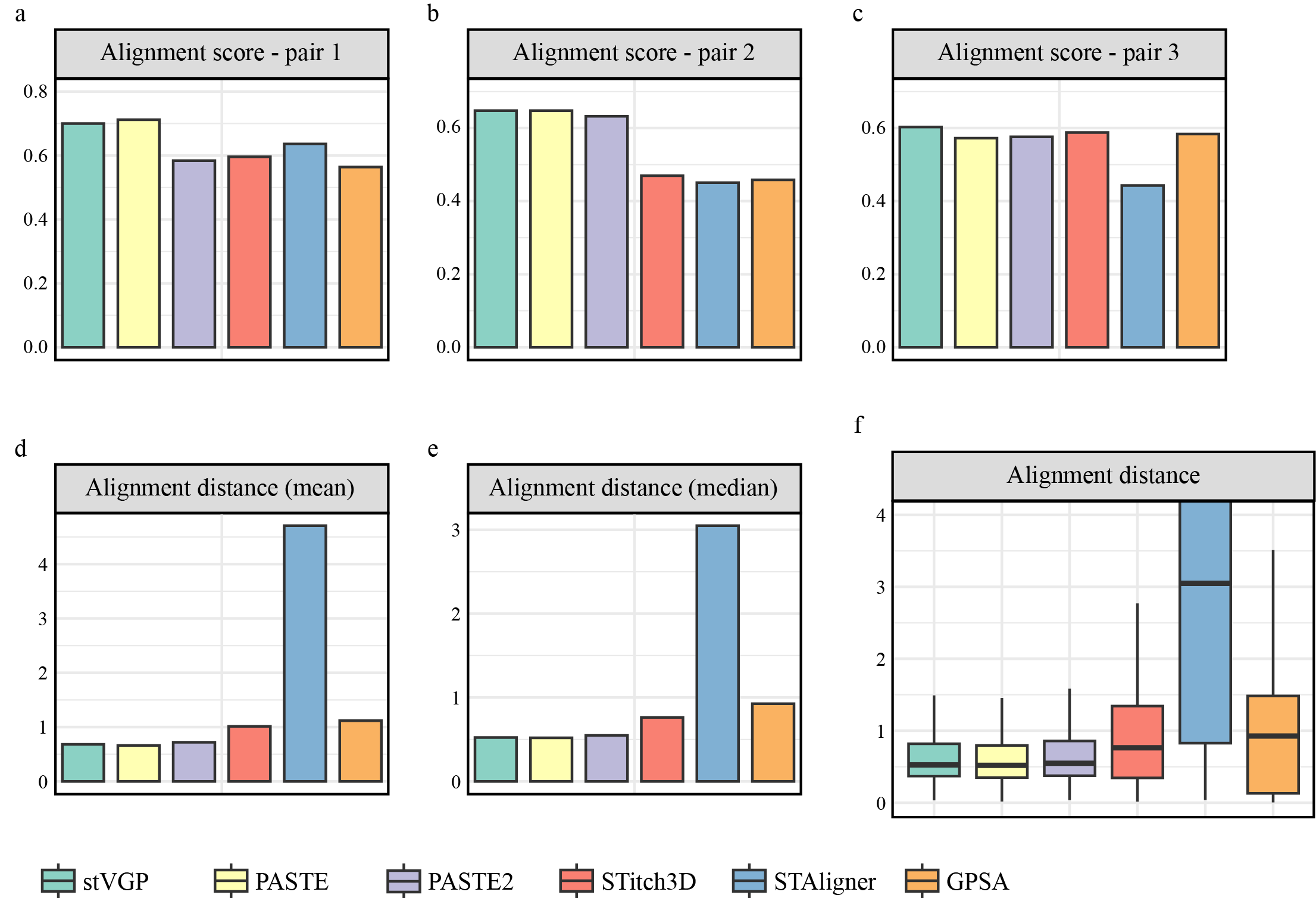


**Supplementary Figure 37**. **Comparison of spatial domain detection methods on the human breast cancer dataset.** This figure presents the spatial distributions of domains identified by various computational methods. Notably, the dataset lacks manual annotations, and only the analytical outputs from each method are shown. Among the results, stVGP, Leiden, Louvain, and STitch3D produced spatial domain patterns that best corresponded to the observable color variations in the tissue.


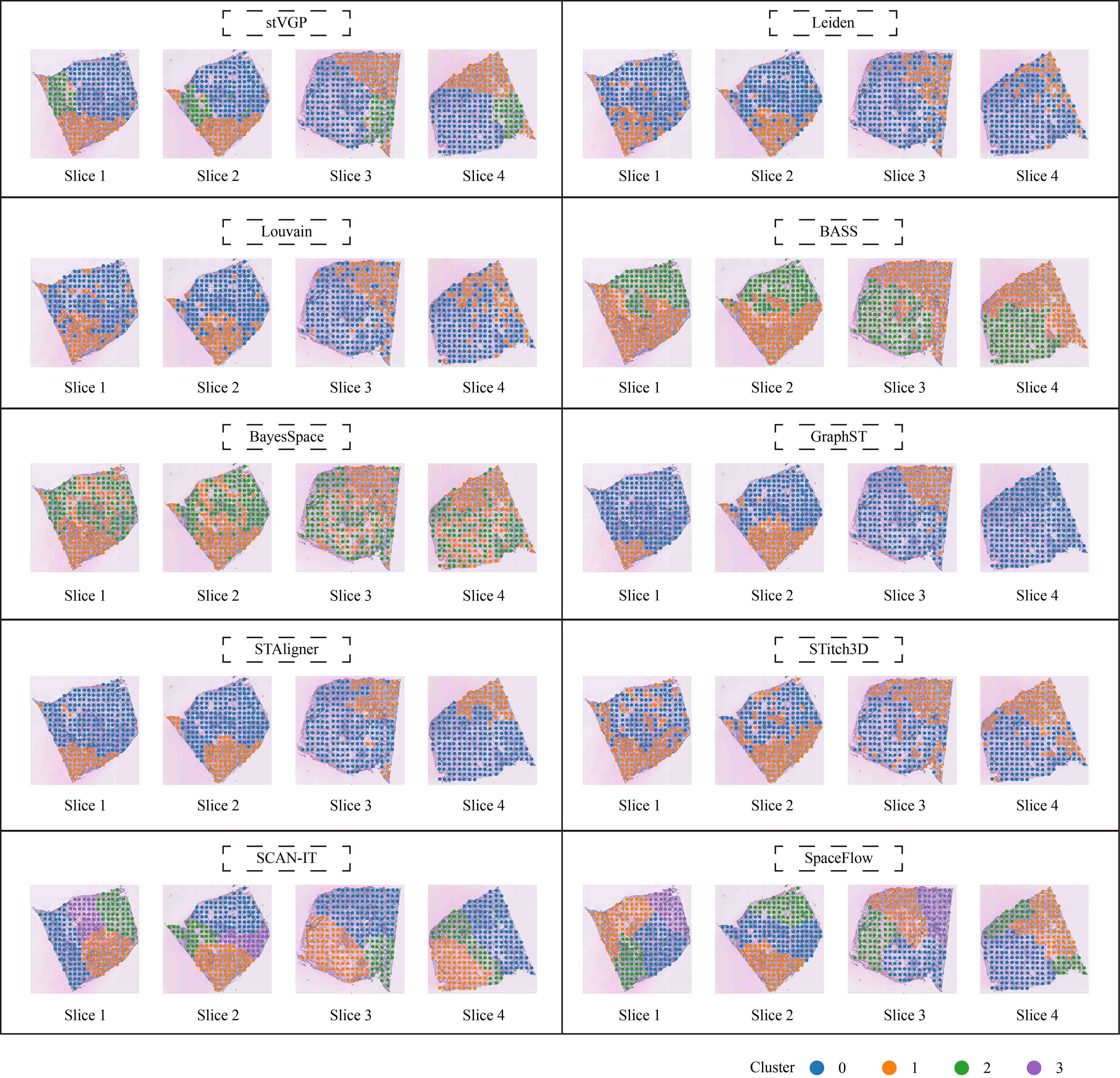


**Supplementary Figure 38**. **UMAP visualization of learned representations across spatial domain detection methods on the human breast cancer dataset.** UMAP plots visualize both clustering patterns and slice identity in the low-dimensional embeddings generated by each method. Among all methods evaluated, stVGP and STitch3D demonstrated superior performance, accurately identifying tissue layering and preserving slice-level information within the learned representations.


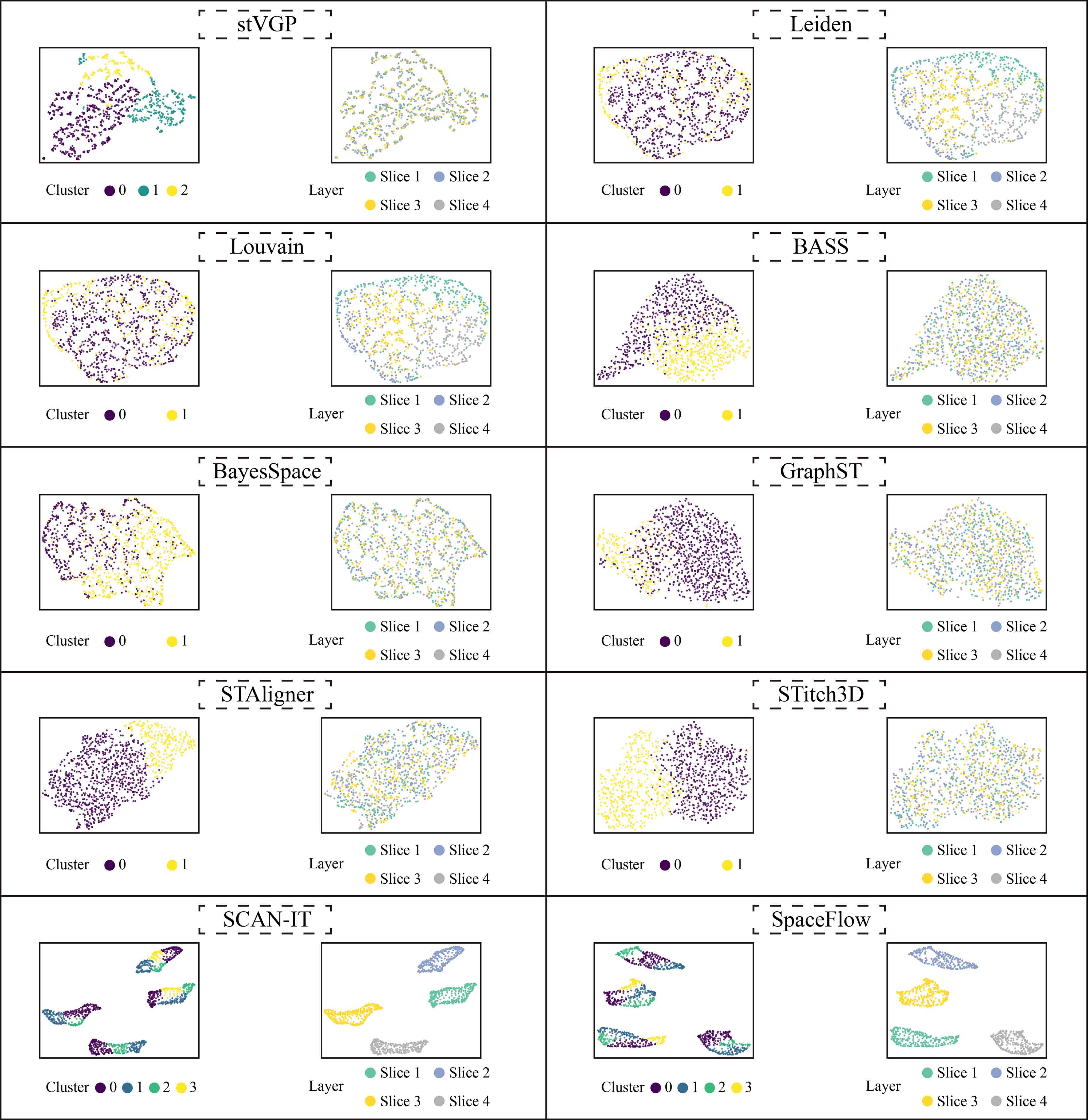


**Supplementary Figure 39**. **Quantitative evaluation of cross-slice gene expression prediction performance. a, b, c, d.** Bar plots summarizing the predictive accuracy for six representative genes (*FN1*, *COL3A1*, *LUM*, *COL1A1*, *SPARC*, *PRSS23*) across four tissue slices (Slice 1–4). Evaluation metrics include Pearson correlation coefficient (a), Spearman's rank correlation coefficient (b), Structural Similarity Index (SSIM) (c), and Root Mean Square Error (RMSE) (d). **e.** Spatial visualization comparing the ground truth expression (top row) and stVGP-predicted expression (bottom row) for two representative genes (*SPARC* and *PRSS23*) across the four analyzed slices.

**
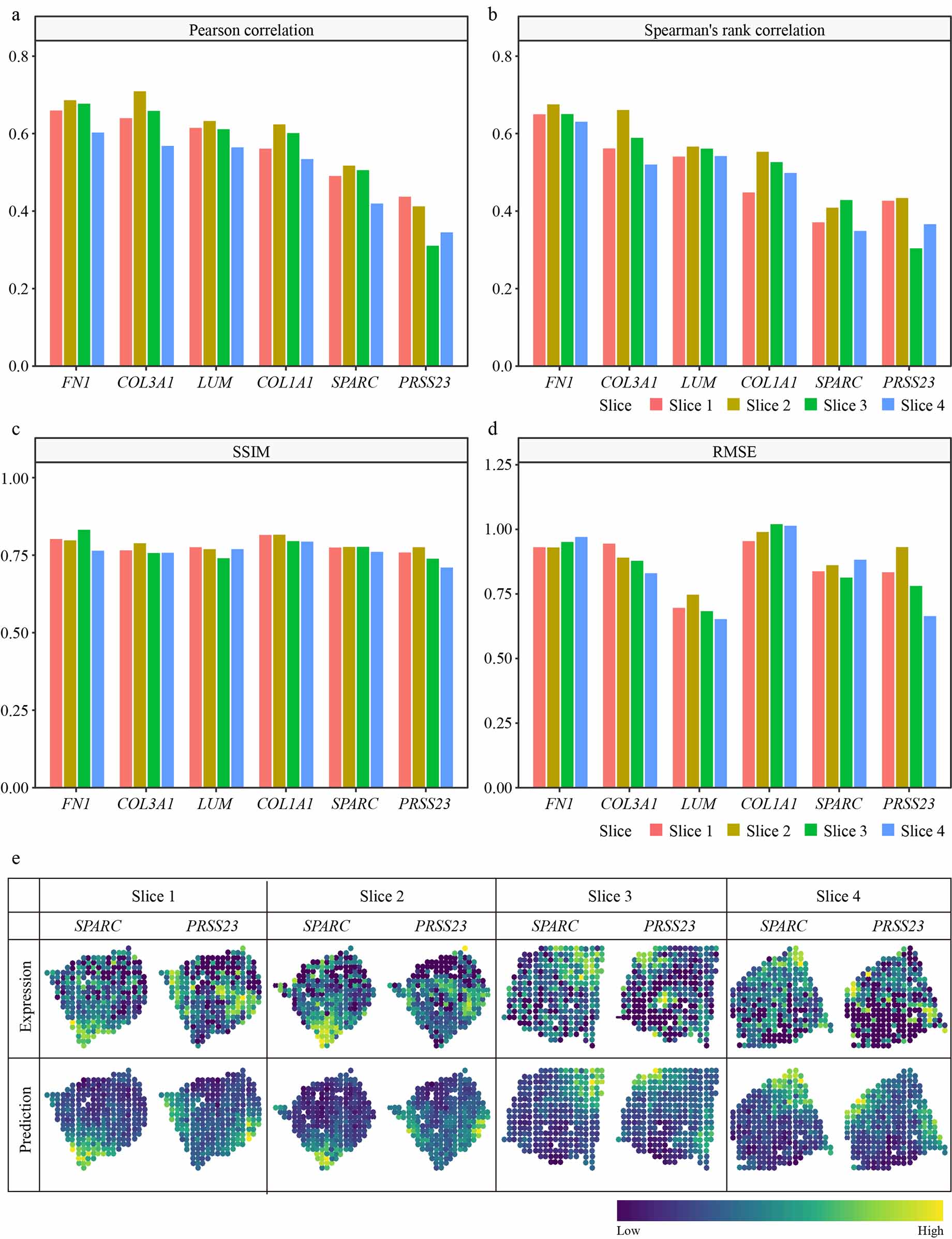
**

**Supplementary Figure 40**. **Sensitivity analysis of stVGP performance to hyperparameter variations. a, b, c, d.** Quantitative assessment of model stability under varying parameter values ranging from 0.01 to 1.0. Performance is evaluated using the Adjusted Rand Index (ARI) for domain recognition (a) and correlation metrics—Kendall's rank correlation (b), Pearson correlation (c), and Spearman's rank correlation (d)—for gene expression prediction. Here we employed the DLPFC dataset for testing, evaluating changes in stVGP domain recognition accuracy and using the *MOBP* gene to assess alterations in reconstruction capability. The bar heights represent the mean score, while error bars indicate the standard deviation derived from 10 independent runs per setting. The results demonstrate the robustness of stVGP, as performance metrics remain stable across a wide range of parameter choices.

**
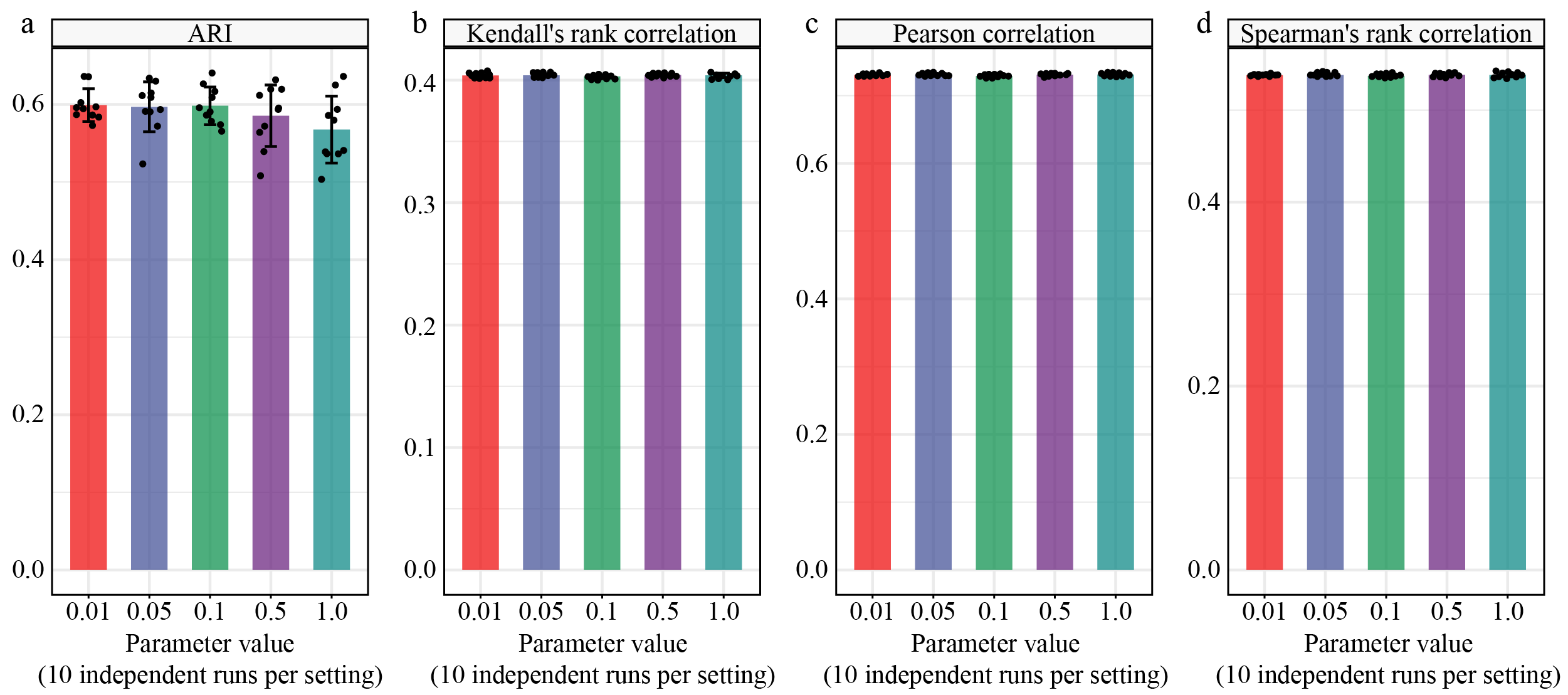
**

**Supplementary Figure 41. Evaluation of stVGP robustness under various data perturbations. a.** Impact of spot resolution on domain recognition performance. The Adjusted Rand Index (ARI) scores are compared between the original resolution and coarser resolutions formed by merging spots (Merge 2: merging two spots into one; Merge 4: merging four spots into one, respectively). **b.** Robustness analysis against missing spatial information. The bar plot displays ARI scores when specific intermediate slices are excluded from the alignment process. **c, d.** Performance degradation analysis under data quality deterioration. Line plots illustrate the trends of ARI scores with increasing data sparsity ratios (c) and elevated noise variance levels (d). The baseline performance (Original) is indicated in red.

**
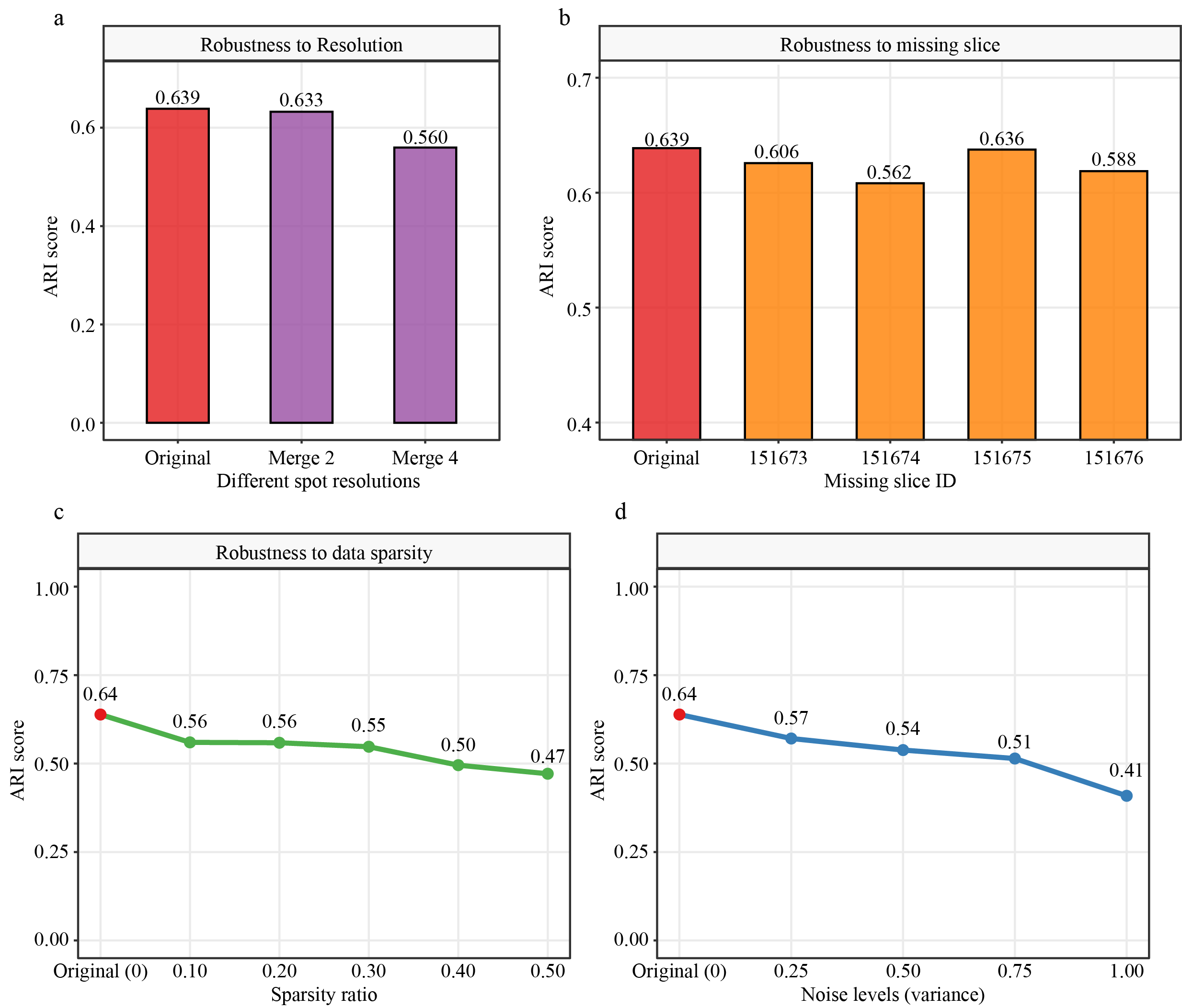
**

**Supplementary Figure 42**. **Impact of fine-tuning strategy on spatial domain identification and gene expression reconstruction. a, b.** Visual comparison of spatial domain recognition results on the human breast cancer dataset using the Fine-tuned model (a) and the Base model without fine-tuning (b) across four slices (151673–151676). The Adjusted Rand Index (ARI) and Normalized Mutual Information (NMI) scores are annotated below each slice to quantify performance. **c, d.** Bar plots summarizing the mean ARI (c) and mean NMI (d) scores, demonstrating the consistent improvement in clustering accuracy yielded by the fine-tuning process. **e.** Spatial visualization of *FN1* and *COL3A1* expression across four slices. The rows display the ground truth expression, predictions from the Fine-tuned model, and predictions from the Base model, highlighting the differences in recovery detail. **f.** Quantitative evaluation of gene prediction accuracy using Pearson correlation and Spearman's rank correlation coefficients for *FN1* and *COL3A1*.

**
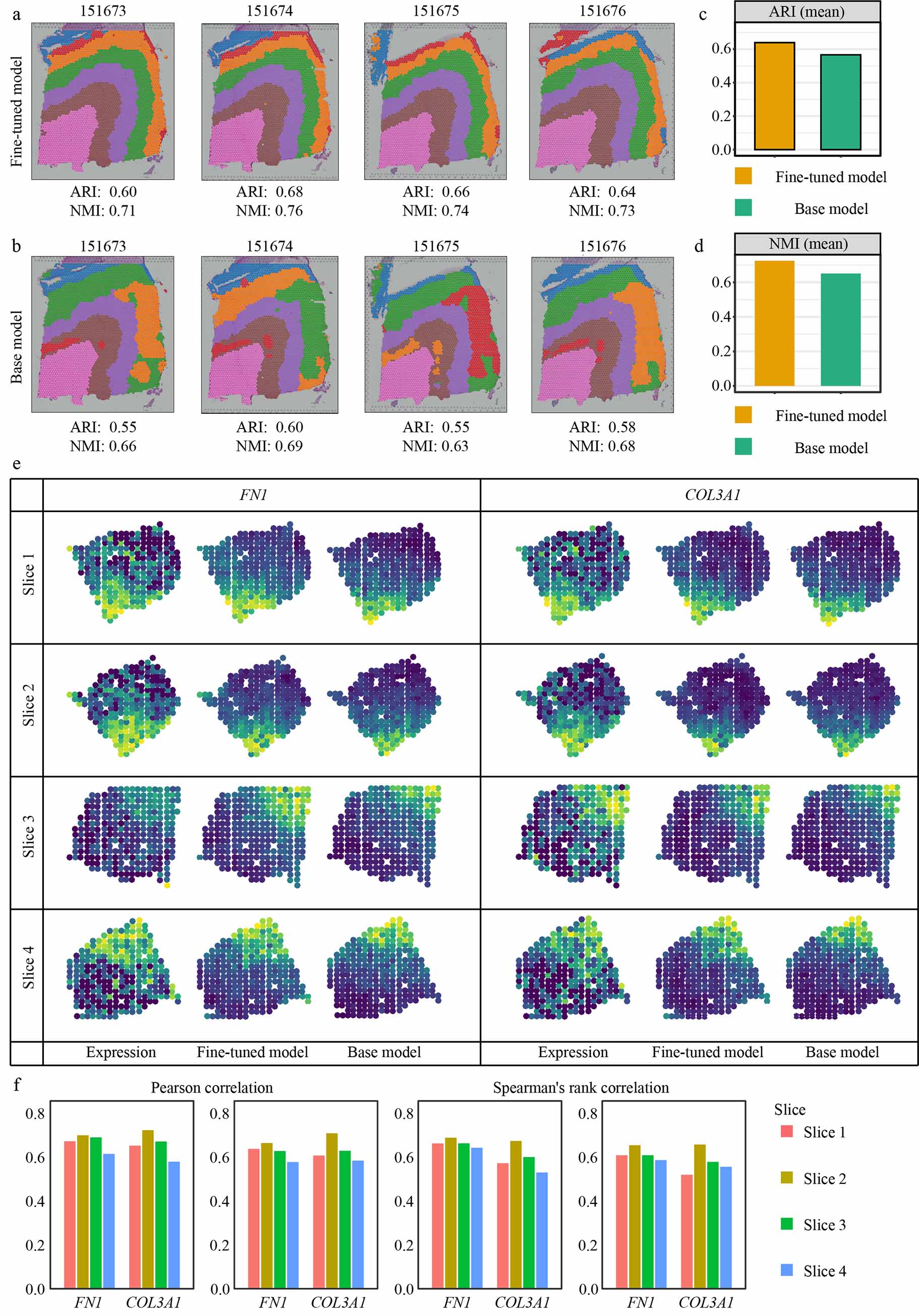
**

**Supplementary Figure 43**. **Scalability analysis of stVGP across varying dataset sizes. a, b, c.** Bar plots illustrating the computational resource consumption of stVGP as the number of spots increases from 1,000 to 17,000. Performance is evaluated in terms of total running time (a), peak CPU memory usage (b), and peak GPU memory usage (c). The specific numerical values are annotated above each bar to quantify the resource requirements at different data scales.


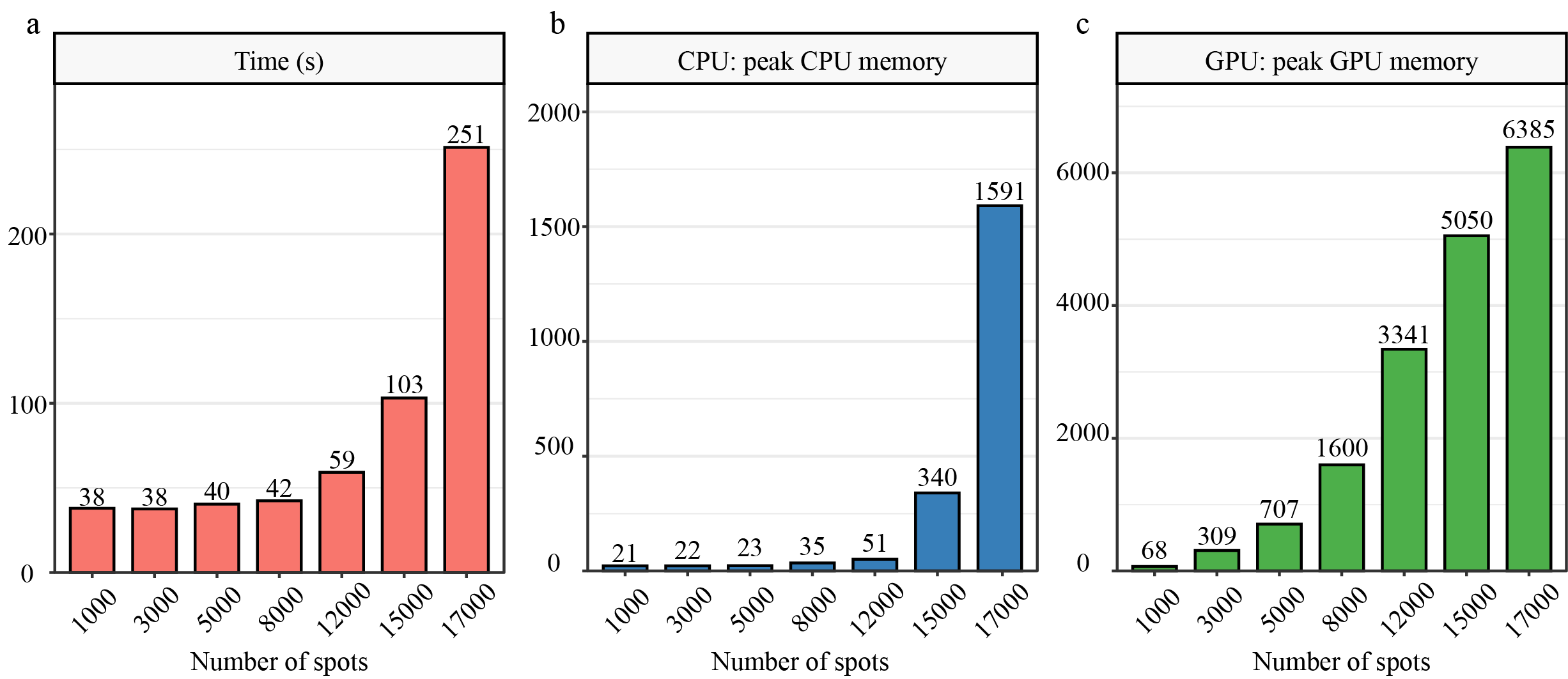


**Supplementary Figure 44**. **Ablation study of spatial alignment components on the mouse brain dataset. a.** Visual comparison of slice alignment results across 35 consecutive sections. The panels display the spatial distribution of spots before alignment and after applying the Full hybrid model, Only rigid alignment, and Only STN (Spatial Transformer Network) alignment. Colors represent different slice indices. **b, c, d.** Quantitative assessment of alignment quality. Performance is evaluated using the mean alignment distance (b), pair-wise alignment scores for adjacent slices (c), and summarized median and mean alignment scores (d). The Full hybrid approach (orange) consistently outperforms single-strategy baselines, achieving lower alignment distances and higher alignment scores.


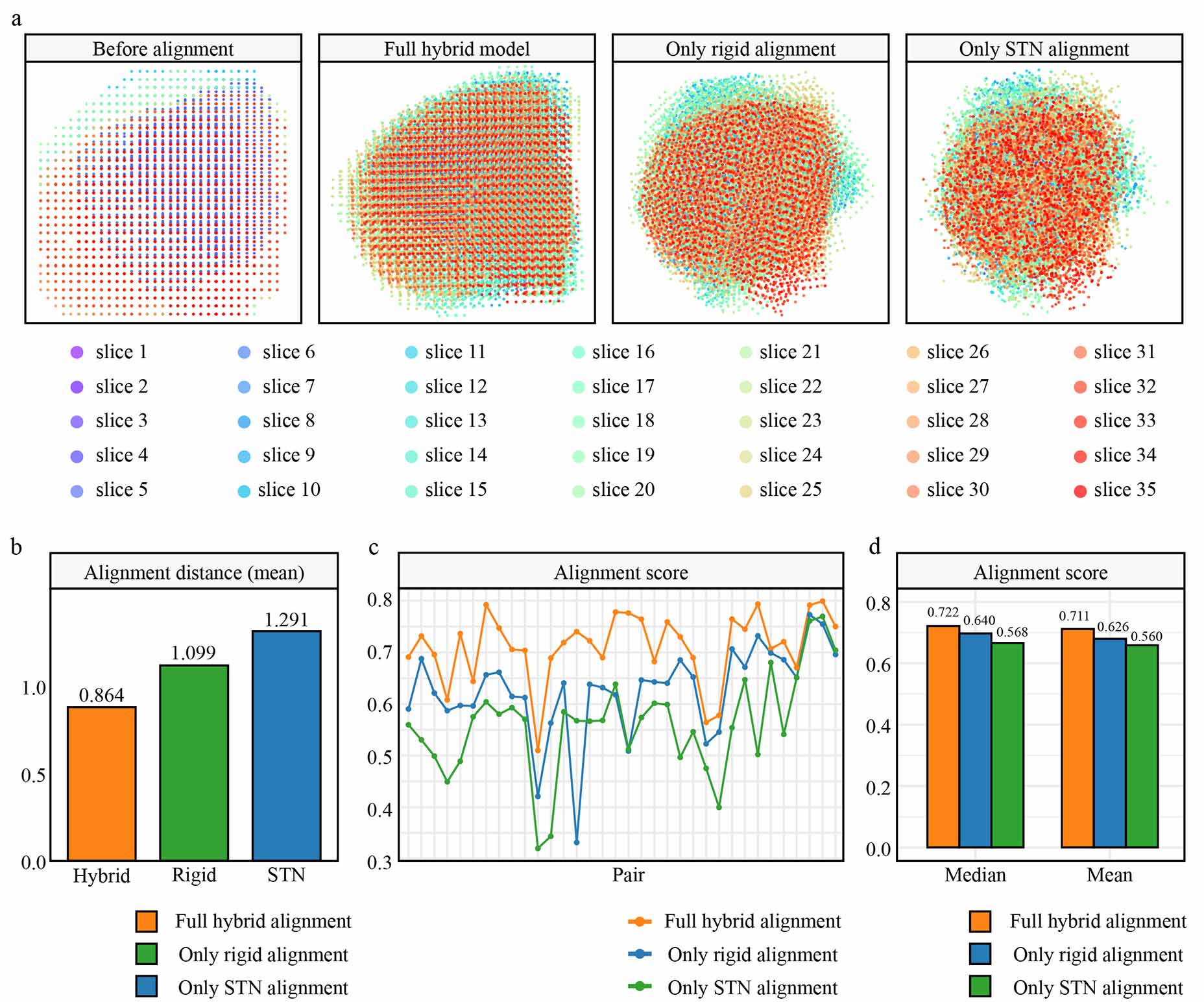


**Supplementary Tables 1. Summary of dataset characteristics: number of slices, number of spots, number of highly variable genes, number of tissue layers, and presence of manual annotations.**

| **ST dataset** | **# slices** | **# spots** | **# gene** | **# manual annotation** | **# tissue layers** |
| --- | --- | --- | --- | --- | --- |
| **Human DLPFC** | 4 | 14364 | 1530 | √ | 7 |
| **Mouse brain** | 35 | 17088 | 1237 | √ | 14 |
| **6.5 PCW human heart** | 9 | 1480 | 1142 | × | × |
| **MOB** | 3 | 825 | 1557 | × | × |
| **Human**  **breast cancer** | 4 | 1031 | 1341 | × | × |

**Supplementary Tables 2. Running time (in seconds) and computational efficiency of stVGP and other spatial alignment methods.**

| **Computation time (Seconds)** | | | | | |
| --- | --- | --- | --- | --- | --- |
| **Method** | Human DLPFC | Mouse brain | 6.5 PCW human heart | MOB | Human  breast cancer |
| **stVGP*** | 561.87 | 1026.6 | 279.81 | 78.19 | 27.62 |
| **PASTE** | 47.7 | 867.4 | 28.9 | 33.1 | 19.2 |
| **PASTE2** | 2465.8 | 3076.3 | 390.7 | 129.0 | 266.6 |
| **STitch3D*** | 38.3 | 726.5 | 41.9 | 51.6 | 36.8 |
| **STAligner*** | 140.1 | 1169.2 | 46.7 | 34.7 | 32.2 |
| **GPSA*** | 8161.5 | 29123.8 | 3213.7 | 17080.2 | 18107.7 |

*****The stVGP, STitch3D, STAligner, and GPSA require a GPU environment, so its runtime had been accelerated by GPU. And the runtime of other methods is the only runtime on CPU.

**Supplementary Tables 3. Peak memory usage (in MB) and computational efficiency of stVGP and other spatial alignment methods.**

| **Peak memory usage (MB)** | | | | | |
| --- | --- | --- | --- | --- | --- |
| **Method** | Human DLPFC | Mouse brain | 6.5 PCW human heart | MOB | Human  breast cancer |
| **stVGP*** | 2181.6 | 13448.2 | 8580.5 | 1243.1 | 1344.5 |
| **PASTE** | 2405.6 | 13855.3 | 3861.4 | 827.7 | 869.0 |
| **PASTE2** | 3230.8 | 15337.2 | 8791.1 | 1095.7 | 1271.6 |
| **STitch3D*** | 2011.0 | 16426.5 | 9100.7 | 1241.8 | 1420.6 |
| **STAligner*** | 3676.0 | 19710.7 | 8473.2 | 1056.1 | 1254.5 |
| **GPSA*** | 1641.7 | 15379.9 | 8477.8 | 1124.1 | 1248.2 |

*****The stVGP, STitch3D, STAligner, and GPSA require a GPU environment, so they not only uses general RAM but also spends a large of video memory. But in this table, we only calculated the usage of general RAM, and were not involved in the usage of video memory.

**Supplementary Tables 4. Running time (in seconds) and computational efficiency of stVGP and other spatial domain detection methods.**

| **Computation time (Seconds)** | | | | | |
| --- | --- | --- | --- | --- | --- |
| **Method** | Human DLPFC | Mouse brain | 6.5 PCW human heart | MOB | Human  breast cancer |
| **stVGP*** | 122.7 | 472.2 | 50.1 | 46.1 | 76.1 |
| **Leiden** | 31.4 | 437.4 | 27.8 | 25.3 | 21.9 |
| **Louvain** | 31.6 | 437.3 | 28.3 | 25.2 | 22.0 |
| **BASS** | 2088.8 | 8382.7 | 361.1 | 298.5 | 229.8 |
| **BayesSpace** | 838.9 | 14818.4 | 737.81 | 435.8 | 546.7 |
| **GraphST*** | 33.7 | 505.4 | 40.2 | 30.6 | 28.4 |
| **STAligner*** | 139.2 | 1156.6 | 46.3 | 34.3 | 32.6 |
| **STitch3D*** | 2614.5 | 4629.4 | 175.3 | 132.2 | 144.3 |
| **SCAN-IT*** | 49.0 | 665.5 | 90.6 | 46.9 | 49.3 |
| **SpaceFlow*** | 90.8 | 768.7 | 96.7 | 59.1 | 53.8 |

*****The stVGP, STitch3D, STAligner, GraphST, SCAN-IT, and SpaceFlow require a GPU environment, so its runtime had been accelerated by GPU. And the runtime of other methods is the only runtime on CPU.

**Supplementary Tables 5. Peak memory usage (in MB) and computational efficiency of stVGP and other spatial domain detection methods.**

| **Peak memory usage (MB)** | | | | | |
| --- | --- | --- | --- | --- | --- |
| **Method** | Human DLPFC | Mouse brain | 6.5 PCW human heart | MOB | Human  breast cancer |
| **stVGP*** | 1963.9 | 15379.8 | 10667.7 | 1124.0 | 1248.2 |
| **Leiden** | 1631.7 | 15369.3 | 6820.2 | 1001.4 | 1312.5 |
| **Louvain** | 1631.5 | 15369.4 | 6820.2 | 1001.4 | 1312.5 |
| **BASS** | 10900.5 | 12696.6 | 4032.9 | 993.1 | 925.6 |
| **BayesSpace** | 7505.1 | 17853.1 | 2850.5 | 617.9 | 683.0 |
| **GraphST*** | 3218.7 | 15372.3 | 8509.7 | 1127.0 | 1333.0 |
| **STAligner*** | 3676.0 | 19692.0 | 8473.2 | 1055.7 | 1254.5 |
| **STitch3D*** | 10282.4 | 25121.2 | 7577.3 | 16328.6 | 3317.3 |
| **SCAN-IT*** | 1773.7 | 15406.9 | 8501.6 | 1161.6 | 1275.3 |
| **SpaceFlow*** | 3799.8 | 15387.9 | 8492.4 | 1142.6 | 1256.3 |

*The stVGP, STitch3D, STAligner, GraphST, SCAN-IT, and SpaceFlow require a GPU environment, so they not only uses general RAM but also spends a large of video memory. But in this table, we only calculated the usage of general RAM, and were not involved in the usage of video memory.

During the runtime recording process (Supplementary Tables 2-5), we observed that among all tested methods, stVGP, STitch3D, and STAligner perform both alignment and spatial domain detection tasks. However, for STitch3D, the alignment task must be completed first to enable domain recognition, while STAligner requires the domain recognition task to be completed first before alignment. As a result, it is challenging to obtain precise single-task runtime measurements. Therefore, we ran the code multiple times, completing the pre-tasks for all three methods before running all tasks to obtain the total runtime for both tasks. Additionally, since STitch3D requires an additional single-cell dataset as input, its memory usage is relatively higher during domain recognition.

These methods were run on 12th Gen Intel(R) Core(TM) i9-12900KF (CPU). stVGP, STitch3D, STAligner, GPSA, GraphST, SCAN-IT, and SpaceFlow require an extra GPU, NVIDIA GeForce RTX 4080.

We can see that stVGP rank 2nd~4th in the 5 datasets for alignment speed test (Supplementary Table 2), and it rank 1st~5th in the 5 datasets for alignment memory test (Supplementary Table 3). At the same time, in domain task stVGP rank 3rd~7th in the 5 datasets for speed test (Supplementary Table 4), and it rank 3rd~10th in the 5 datasets for memory test (Supplementary Table 5).

**Supplementary Tables 6. Glossary of Terms.**

| **Terms** | **Definition** |
| --- | --- |
| **Alignment distance** | The Euclidean distance calculated for each spot in the aligned slice to its nearest neighbor sharing the identical label in the adjacent reference slice. This metric quantifies the local geometric accuracy of the alignment. |
| **Alignment score** | A quantitative metric ranging from 0 to 1 that evaluates alignment precision. It is defined as the proportion of spots in the aligned slice whose spatially nearest neighbor in the template slice shares the identical domain label. Higher scores indicate superior preservation of biological spatial patterns. |
| **Spatial domain** | A coherent tissue region characterized by distinct gene expression profiles and spatial continuity. These domains are computationally identified by clustering algorithms (e.g., stVGP) that integrate molecular data with spatial coordinates, often corresponding to anatomical structures or functional tissue units. |
| **Coherent domain** | An algorithmically identified tissue region that exhibits spatial continuity and molecular consistency not only within a single slice but also spanning multiple adjacent slices, effectively representing a continuous 3D biological structure. |
| **Tissue domain** | Spatially coherent regions initially identified by the algorithm and subsequently annotated with specific biological functions or anatomical identities (e.g., ventricular myocardium) based on marker gene expression. |
| **Virtual slice** | A computationally inferred spatial transcriptomics slice generated by stVGP at an unobserved z-coordinate. These synthetic slices interpolate biological information between physical sections to facilitate continuous 3D tissue reconstruction. |

**Reference**

1 Zeira, R., Land, M., Strzalkowski, A. & Raphael, B. J. Alignment and integration of spatial transcriptomics data. *Nature Methods* **19**, 567-575, doi:10.1038/s41592-022-01459-6 (2022).

2 Liu, X., Zeira, R. & Raphael, B. J. Partial alignment of multislice spatially resolved transcriptomics data. *Genome research* **33**, 1124-1132, doi:10.1101/gr.277670.123 (2023).

3 Wang, G. *et al.* Construction of a 3D whole organism spatial atlas by joint modelling of multiple slices with deep neural networks. *Nature Machine Intelligence* **5**, 1200-1213, doi:10.1038/s42256-023-00734-1 (2023).

4 Zhou, X., Dong, K. & Zhang, S. Integrating spatial transcriptomics data across different conditions, technologies and developmental stages. *Nature Computational Science* **3**, 894-906, doi:10.1038/s43588-023-00528-w (2023).

5 Jones, A., Townes, F. W., Li, D. & Engelhardt, B. E. Alignment of spatial genomics data using deep Gaussian processes. *Nature Methods* **20**, 1379-1387, doi:10.1038/s41592-023-01972-2 (2023).

6 Traag, V. A., Waltman, L. & van Eck, N. J. From Louvain to Leiden: guaranteeing well-connected communities. *Scientific Reports* **9**, 5233, doi:10.1038/s41598-019-41695-z (2019).

7 Wolf, F. A., Angerer, P. & Theis, F. J. SCANPY: large-scale single-cell gene expression data analysis. *Genome Biology* **19**, 15, doi:10.1186/s13059-017-1382-0 (2018).

8 Shao, X. *et al.* Knowledge-graph-based cell-cell communication inference for spatially resolved transcriptomic data with SpaTalk. *Nature Communications* **13**, 4429, doi:10.1038/s41467-022-32111-8 (2022).

9 Zhao, E. *et al.* Spatial transcriptomics at subspot resolution with BayesSpace. *Nat Biotechnol* **39**, 1375-1384, doi:10.1038/s41587-021-00935-2 (2021).

10 Long, Y. *et al.* Spatially informed clustering, integration, and deconvolution of spatial transcriptomics with GraphST. *Nature Communications* **14**, 1155, doi:10.1038/s41467-023-36796-3 (2023).

11 Cang, Z., Ning, X., Nie, A., Xu, M. & Zhang, J. SCAN-IT: Domain segmentation of spatial transcriptomics images by graph neural network. *BMVC : proceedings of the British Machine Vision Conference. British Machine Vision Conference* **32** (2021).

12 Ren, H., Walker, B. L., Cang, Z. & Nie, Q. Identifying multicellular spatiotemporal organization of cells with SpaceFlow. *Nature Communications* **13**, 4076, doi:10.1038/s41467-022-31739-w (2022).

13 Shi, X., Jiang, X. & Álvarez, M. *Neighbour-Driven Gaussian Process Variational Autoencoders for Scalable Structured Latent Modelling*. (2025).

14 Li, Z. & Zhou, X. BASS: multi-scale and multi-sample analysis enables accurate cell type clustering and spatial domain detection in spatial transcriptomic studies. *Genome Biology* **23**, 168, doi:10.1186/s13059-022-02734-7 (2022).

15 Zhao, E. *et al.* Spatial transcriptomics at subspot resolution with BayesSpace. *Nature Biotechnology* **39**, 1375-1384, doi:10.1038/s41587-021-00935-2 (2021).

16 Maynard, K. R. *et al.* Transcriptome-scale spatial gene expression in the human dorsolateral prefrontal cortex. *Nat Neurosci* **24**, 425-436, doi:10.1038/s41593-020-00787-0 (2021).

17 Ortiz, C. *et al.* Molecular atlas of the adult mouse brain. *Sci Adv* **6**, eabb3446, doi:10.1126/sciadv.abb3446 (2020).

18 Asp, M. *et al.* A Spatiotemporal Organ-Wide Gene Expression and Cell Atlas of the Developing Human Heart. *Cell* **179**, 1647-1660.e1619, doi:https://doi.org/10.1016/j.cell.2019.11.025 (2019).

19 Ståhl, P. L. *et al.* Visualization and analysis of gene expression in tissue sections by spatial transcriptomics. *Science (New York, N.Y.)* **353**, 78-82, doi:10.1126/science.aaf2403 (2016).

20 Hu, J. *et al.* SpaGCN: Integrating gene expression, spatial location and histology to identify spatial domains and spatially variable genes by graph convolutional network. *Nature Methods* **18**, 1342-1351, doi:10.1038/s41592-021-01255-8 (2021).

21 Biancalani, T. *et al.* Deep learning and alignment of spatially resolved single-cell transcriptomes with Tangram. *Nature Methods* **18**, 1352-1362, doi:10.1038/s41592-021-01264-7 (2021).

22 Wei, R. *et al.* Spatial charting of single-cell transcriptomes in tissues. *Nature Biotechnology* **40**, 1190-1199, doi:10.1038/s41587-022-01233-1 (2022).

23 Yuan, Z. MENDER: fast and scalable tissue structure identification in spatial omics data. *Nature Communications* **15**, 207, doi:10.1038/s41467-023-44367-9 (2024).

24 Korsunsky, I. *et al.* Fast, sensitive and accurate integration of single-cell data with Harmony. *Nature Methods* **16**, 1289-1296, doi:10.1038/s41592-019-0619-0 (2019).

25 Xu, C. *et al.* DeepST: identifying spatial domains in spatial transcriptomics by deep learning. *Nucleic Acids Research* **50**, e131-e131, doi:10.1093/nar/gkac901 %J Nucleic Acids Research (2022).

26 Dong, K. *et al.* Benchmarking multi-slice integration and downstream applications in spatial transcriptomics data analysis. *Genome Biol* **26**, 318, doi:10.1186/s13059-025-03796-z (2025).

27 Buijtendijk, M. F. J., Barnett, P. & van den Hoff, M. J. B. Development of the human heart. *American journal of medical genetics. Part C, Seminars in medical genetics* **184**, 7-22, doi:10.1002/ajmg.c.31778 (2020).

28 Fraley, C. & Raftery, A. MCLUST: Software for model-based cluster and discriminant analysis. *Department of Statistics, University of Washington: Technical Report* **342**, 1312 (1998).

29 de Bakker, B. S. *et al.* An interactive three-dimensional digital atlas and quantitative database of human development. *Science (New York, N.Y.)* **354**, doi:10.1126/science.aag0053 (2016).

30 Ståhl, P. L. *et al.* Visualization and analysis of gene expression in tissue sections by spatial transcriptomics. **353**, 78-82, doi:doi:10.1126/science.aaf2403 (2016).
